# RNAlien – Unsupervised RNA family model construction

**DOI:** 10.1093/nar/gkw558

**Published:** 2016-06-21

**Authors:** Florian Eggenhofer, Ivo L Hofacker, Christian Höner zu Siederdissen

**Affiliations:** 1 Institute for Theoretical Chemistry, University of Vienna, Währingerstrasse 17, A-1090 Vienna, Austria; 2 Bioinformatics Group, Department of Computer Science University of Freiburg, Georges-Köhler-Allee, 79110 Freiburg, Germany; 3 Research Group Bioinformatics and Computational Biology, Faculty of Computer Science, University of Vienna, A-1090 Vienna, Austria; 4 Bioinformatics Group, Department of Computer Science, University of Leipzig, D-04107 Leipzig, Germany; 5 Interdisciplinary Center for Bioinformatics, University of Leipzig, Härtelstraße 16-18, D-04107 Leipzig, Germany

## Abstract

Determining the function of a non-coding RNA requires costly and time-consuming wet-lab experiments. For this reason, computational methods which ascertain the homology of a sequence and thereby deduce functionality and family membership are often exploited. In this fashion, newly sequenced genomes can be annotated in a completely computational way. Covariance models are commonly used to assign novel RNA sequences to a known RNA family. However, to construct such models several examples of the family have to be already known. Moreover, model building is the work of experts who manually edit the necessary RNA alignment and consensus structure. Our method, RNAlien, starting from a single input sequence collects potential family member sequences by multiple iterations of homology search. RNA family models are fully automatically constructed for the found sequences. We have tested our method on a subset of the RfamRNA family database. RNAlien models are a starting point to construct models of comparable sensitivity and specificity to manually curated ones from the Rfam database. RNAlien Tool and web server are available at http://rna.tbi.univie.ac.at/rnalien/.

## INTRODUCTION

One of the basic aims of genome informatics is to annotate every single nucleotide of a genome for presence and type of biological function. The most well-known regions are protein-coding genes. The nature of non-coding RNAs (ncRNAs) and their genes has more recently started to play a role (1), with many new functions of these non-protein coding regions being elucidated using biological (2) and computational methodology (3). Of particular interest are ncRNAs which form well-defined structures that are needed to perform their function.

The sequence and the structural conservation of RNAs allows for clustering these ncRNAs into families of homologs. Structural RNA families are therefore conveniently characterized by a multiple alignment, as well as a consensus secondary structure. This allows one to trace patterns of structural conservation with covariance-preserving sequence mutations through the evolution of individual ncRNAs. For sequences that are not too far diverged, it has become a standard procedure to determine RNA family membership via computational means.

When newly sequenced genomes are to be annotated for putative functions, several tools exist that try to match a known structural RNA family to an area of the genome. The Infernal (4,5) suite of tools provides the standard machinery to match known structural RNA family models to genomic regions. The required family models are collected in the Rfam (6,7) database of more than 2000 families.

Novel RNA sequences, which are continuously discovered via next-generation sequencing experiments, are often the first known example of their RNA family. It is therefore of interest to search for homologous sequences in related species and ultimately construct a covariance model.

RNA homology search is a difficult problem (8), since simple sequence-based search can only detect very close homologs while structural conservation is needed to reliably detect remote homologs. The traditional approach would therefore combine sequence-based BLAST searches with manual inspection of each candidate in order to discard spurious hits without structural similarity.

Successful homology search for some families (9–13) that are highly variable in length and structure even requires context information like associated promoter regions. For some families even specialized homology search tools exist that consider their individual properties (14–17). Once a set of diverse family members has been collected, a covariance model can be constructed from the final alignment and consensus structure. From that point on it would be possible to use the Rfam pipeline for iteratively expanding the seed alignment (14). The model can then be submitted for review, in essence repeating the steps already taken for known RNA families in the Rfam database.

The above approach is, especially up to the seed alignment, quite time-consuming and individual steps like choosing the exact start and end of the potential candidate are not standardized. In short, the model construction process would greatly profit from automation and standardization.

We now describe in detail the approach we have taken for automating the construction of a set of potentially homologous sequences given a single starting sequence, including the prediction of a common consensus secondary structure.

Our approach closely mimics a strategy that could be employed when searching for homologous sequences manually. Given that our method scales to many sequences and can be off-loaded to a web service, it aims to decrease the burden of establishing initial family models for novel sequences without much local overhead for the user.

## MATERIALS AND METHODS

RNAlien is based on an iterative sequence search process. In each step new sequences from a different section of the phylogenetic tree are searched for, filtered and possibly included in the growing RNA family model. By step-wise inclusion of remote family members it is possible to increase the sensitivity for even more divergent members, without losing too much specificity.

**Figure 1.**
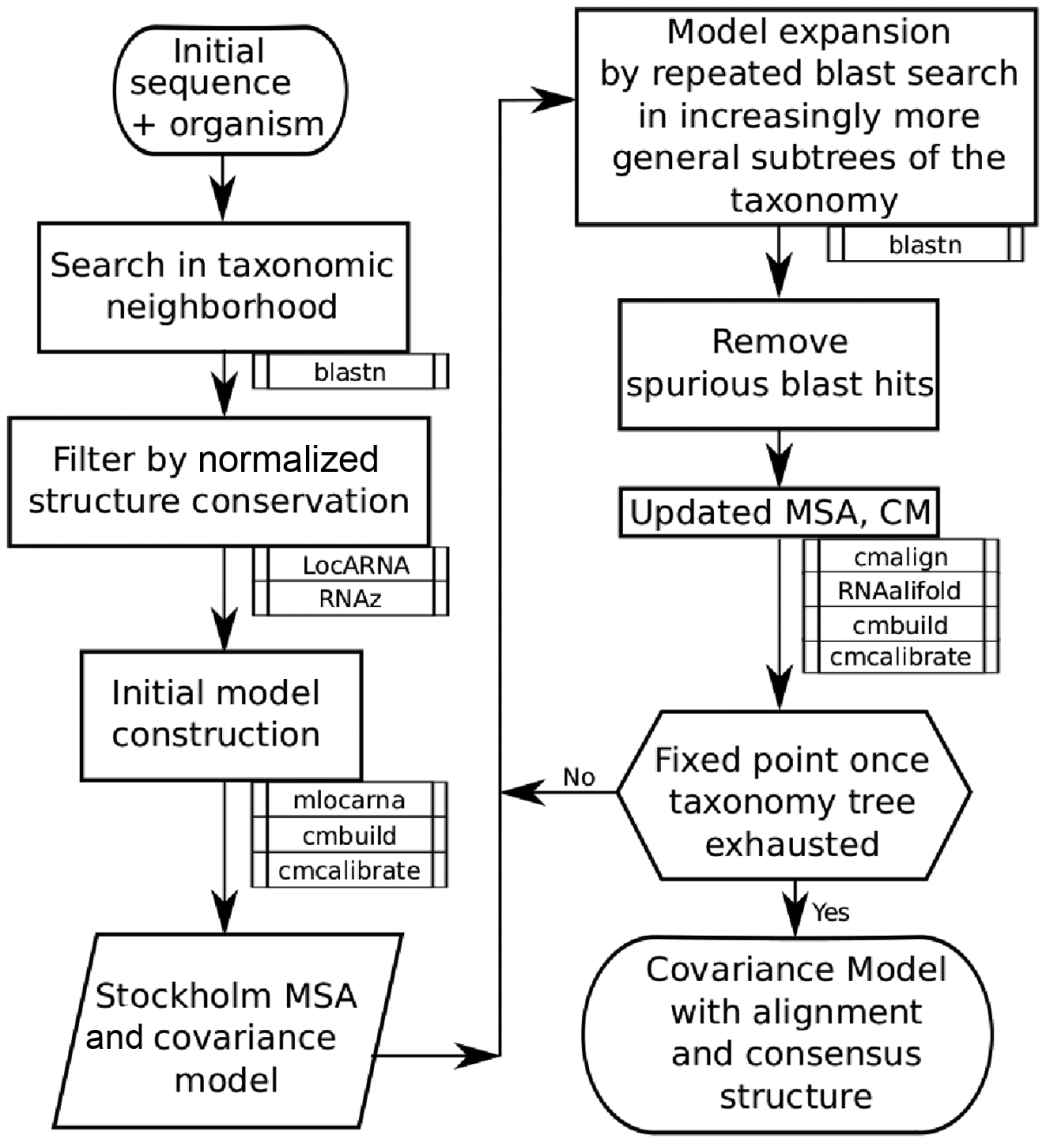
RNAlien program flow. RNAlien expects a single input sequence for which homology is to be established. Knowledge of the source organism provides an optional starting point in the taxonomic tree. Sequence-similar candidates are discovered (via BLAST) in closely related species with selection to reduce bias. Once a small set is discovered, an initial structural alignment and covariance model are constructed (as shown on the left) with mlocarna, cmbuild and cmcalibrate. In the second step (shown on the right side), BLAST searches continue to ever more divergent species. The covariance model is used to decide if these additional sequences are included and, if so, aligned to the model. When the whole taxonomic tree has been visited, a final search is performed and then the resulting covariance model, structural alignment and all collected sequences are returned.

In brief, RNAlien starts with a single sequence and optionally the organism of origin, identified by the NCBI taxonomy (18) identifier as input. An initial RNA family model is constructed from sequences found in the close taxonomic neighborhood of the input. In the second phase, the model is expanded iteratively by ascending in the taxonomic tree, and considering ever larger sub-trees, to collect family members from increasingly divergent species. When the root of the tree has been reached a final global search in each taxonomic kingdom is performed, to include sequences of interest that could not be identified before. Figure 1 shows an overview of the pipeline, a more detailed flowchart (Supplementary Figure S1) and default parameter set (Supplementary Section B – Implementation details) are available in the Supplementary Material.

### Initial model construction

The goal of the initial model construction is to collect sequences that capture the secondary structure of the RNA family and some sequence variability that allows us to find more remote homologs.

RNAlien performs a sequence search via the NCBI nucleotide Blast REST interface and restricts the search to the taxonomic parent of the input organism. Using the REST interface has the advantage that the scanned databases are always up to date and that no bulk downloads are necessary.

BLAST hits are pre-filtered by having more than 80% coverage of the query sequence to exclude short hits. Collection of redundant hits is avoided by excluding hits with 99% or more query similarity.

Since BLAST hits are usually too short, we first expand them with flanking genomic regions (see Supplementary subsection B.5). Subsequently each candidate sequence is aligned to the input sequence using the structural RNA alignment program LocARNA (19) with a semi-global alignment in order to truncate them to the input sequence length.

The sequence identity *SI* is used as a measure for sequence conservation. Given the Levenshtein distance *D* between the input and current candidate sequence, and *L*, the length of the longer of the two sequences, we calculate the *SI* as follows: *SI* = 1 − (*D*/*L*)

Since we are interested in structural RNAs, we want to accept candidates that exhibit more structure conservation than expected for their respective sequence similarity. As a measure of structure conservation we use the *SCI* value introduced in the RNA gene finder RNAz (20). The *SCI* compares the energy *E*_consensus_ of a consensus structure folding of the alignment $\mathcal {A}$ with the average energy $\overline {E_{{\rm x}}}$ obtained from folding each sequence *x* in the alignment individually, $SCI = E_{{\rm consensus}} / \overline {E_{{\rm x}}}$. Since the *SCI* depends on the sequence identity of the alignment (an alignment of identical sequences necessarily has *SCI* = 1), we normalize the *SCI* by the sequence identity *SI* of the sequences:

(1) \begin{equation*} nSCI = \frac{SCI}{SI} \end{equation*}

As a rule of thumb, alignments of structured RNA families exhibit an *SCI* larger than the sequence identity. We therefore accept candidates if their *nSCI* > 1.

In case the first round does not yield any acceptable candidates, we ascend in the NCBI taxonomic tree and repeat the initial model construction in the larger sub-tree.

All accepted sequences in the initial set are aligned with mlocarna, the multiple sequence alignment variant of LocARNA. The resulting structural alignment is then used to construct and calibrate a covariance model with cmbuild and cmcalibrate from the Infernal package. We speed up calibration as described in Supplementary Material B.1 – Model construction. In the following round of the model expansion phase this model will be used to decide candidate sequence acceptance.

### Model expansion

Model expansion is an iterative process depending on the family members collected so far and the corresponding covariance model.

The first step is to select representative queries for the upcoming BLAST search from the currently collected sequences. The current set is filtered, so that for all sequences with pairwise similarity greater than 95% only the first one is used. Per default the first five of these sequences are used as query sequences.

Optionally the current set can instead be clustered with the UPGMA algorithm (21), based on a distance matrix computed by Clustal Omega (22). RNAlien incrementally increases the cluster cutoff distance to form up to 5 clusters. The first sequence from each cluster is used as a query sequence. This method achieved slightly better recall in the benchmark but is optional due to the Clustal Omega dependency.

The target organisms are always confined to a sub-tree of the taxonomy. In each round the search space is expanded by ascending one level in the taxonomy. In order to avoid duplicates we also exclude the sub-tree of the previous round (see Supplementary Figure S2). For example, if the current taxonomic position is *Enterobacteriacea* (family) and the previous node was *Enterobacter* (genus) all organisms that belong to *Enterobacteriacea* but not *Enterobacter* are searched. Depending on the number of selected queries, multiple searches can be performed, the results are then pooled. The search is again performed via the REST interface of NCBI nucleotide BLAST using an E-value cutoff of 1.

To decide which of the BLAST hits to accept, we evaluate each hit with the current covariance model using cmsearch. To obtain E-values we set the genome size parameter of cmsearch to the database size of the BLAST search. At this step, we employ two different E-value cutoffs: Sequences that satisfy the strict cutoff (E-value < 0.001) are accepted and used to build the next iteration of the covariance model. Sequences that only satisfy a relaxed cutoff of 1, are collected in a set of ‘potential’ family members and re-evaluated at the end of the pipeline using the final model.

Candidates that have been accepted are aligned to the model by cmalign, which creates a new Stockholm alignment. The expanded alignment may yield a slightly changed consensus structure compared to the previous iteration. We therefore recompute the consensus structure using RNAalifold with the recommended parameters from (23). A new model is then constructed with cmbuild and calibrated (see Supplementary Material B.1 – Model construction) with cmcalibrate. Model expansion proceeds further up in the taxonomic tree until the root node has been reached.

### Model finalization

In order to capture the most remote homologs, a final round analogous to model expansion, but without any taxonomic restriction is performed.

Finally, the set of potential family members collected during earlier rounds is now re-evaluated with the current model using the strict cutoff. This gives rise to the final covariance model, which is once more calibrated using cmcalibrate.

### Model evaluation

The final covariance model and the corresponding structural alignment are inspected via cmstat, RNAz, RNAcode (24) and taxonomy of the included sequences. RNAz predicts whether the alignment contains a functional RNA structure. Since RNAlien is particularly geared for structural RNAs, this is an important quality indicator. cmstat provides additional information about the resulting covariance model itself, such as the total and effective number of sequences used to construct the model and the relative importance of sequence and structure information.

RNAcode predicts protein coding segments within the alignment. This allows in particular to identify RNAs that carry both functional open reading frames and RNA structure. While it is possible to use RNAlien for pure protein coding sequences, methods that consider protein specific features are more suited. For all found sequences a lookup at RNAcentral (25,26) is performed to find already existing entries. A list of RNAcentral identifiers is appended to the result.

The taxonomy information of the collected sequences can be useful for gaining information about the biological function of a newly isolated RNA. RNAlien provides a detailed log of tools and exact versions as well as intermediate results for later analysis and reproducibility of the construction process.

## RESULTS AND DISCUSSION

In order to test the quality of the automatic family construction process, two different performance tests were conducted. First, we extracted a subset of RNA families from the Rfam 12.0 database, as detailed below. We then used RNAlien to reconstruct each RNA family, given a single sequence from the seed alignment. The resulting family model and collected sequences were compared with the original Rfam model and sequences. This test reveals the ability of RNAlien to reconstruct a known family from a single sequence.

The resulting consensus secondary structures from the first test were compared against the structure annotated in the seed alignment and the run-time for RNAlien was measured.

Second, we created a set of negative control sequences and started the model construction process. We used coding sequences, ancestral repeats, untranslated regions (UTRs) from NCBI genbank (27), Ensembl Release 83 (28), RegulonDB 9.0 (29) and random sequences. According to the procedure for structured and diverse RNA families the sequences of the negative control set were used as a input sequence for RNAlien.

### Rfam families with known structure

As a test set we chose the subset of Rfam families with known structure derived from nuclear magnetic resonance or X-ray crystallography. For efficiency reasons, we discarded three families that are representing large ribosomal sub-units, each consisting of sequences exceeding 1500 nucleotides in length, leaving us with 56 families. A second test set with 192 families is contained in the Supplementary Material (see Supplementary Section D).

By arbitrary choice the first sequence of the Rfam seed-alignment was extracted and the organism of origin retrieved. This single initial sequence and the corresponding taxonomy id were used as input to RNAlien. To measure the specificity of RNAlien we tested each of the homologs predicted by RNAlien using the Rfam covariance model. RNAlien predictions that did not meet the bit score cutoff, as described below, of the Rfam model were considered false positives.

Conversely, we measured the recall of the RNAlien model by evaluating all sequences in the Rfam seed alignment and counting all sequences not recognized by the RNAlien model as false negatives.

To provide a context for the results we performed both a BLAST and a nhmmer (30) search against the full NCBI nucleotide database with each RNAlien input sequence, without iteration. The BLAST results were aligned with mlocarna and a consensus structure was computed with RNAalifold. For the nhmmer result alignment a consensus structure was computed with RNAalifold.

The Bacterial small subunit ribosomal RNA homology search nhmmer found over 2 million hits and the resulting structural alignment was too big to further process (∼600 GB) it. We therefore included it with specificity and sensitivity 1.

The resulting alignments for both tools were used to construct and calibrate a covariance model. The sequences and the model were used in the same manner as the alien result models for the benchmark.

We used two different cutoffs, one bit score based for specificity and one E-value based for the recall benchmark. The bit score cutoff uses the gathering cutoff annotated for the Rfam model to discriminate between true and false positives. However, the gathering score is quite specific for the Rfam model and is possibly not applicable to the RNAlien model.

Therefore, we used a E-value cutoff for cmsearch of 0.001 with a database size of 1000 × 10^6^ nucleotides for families with members in eukaryotic species, corresponding to typical genome sizes. For families predominantly present in viral and prokaryotic species 1 × 10^6^ nucleotides was set as database size.

Note, that there may well exist true homologs that are not recognized by the Rfam covariance model. Moreover, some classes of RNA, such as *RNaseP* or *SRP*RNA, are represented in Rfam by multiple families. The reported accuracies therefore present a pessimistic estimate. All intermediate results and models from this benchmark are available via *http://rna.tbi.univie.ac.at/rnalien/help#benchmark*.

**Figure 2.**
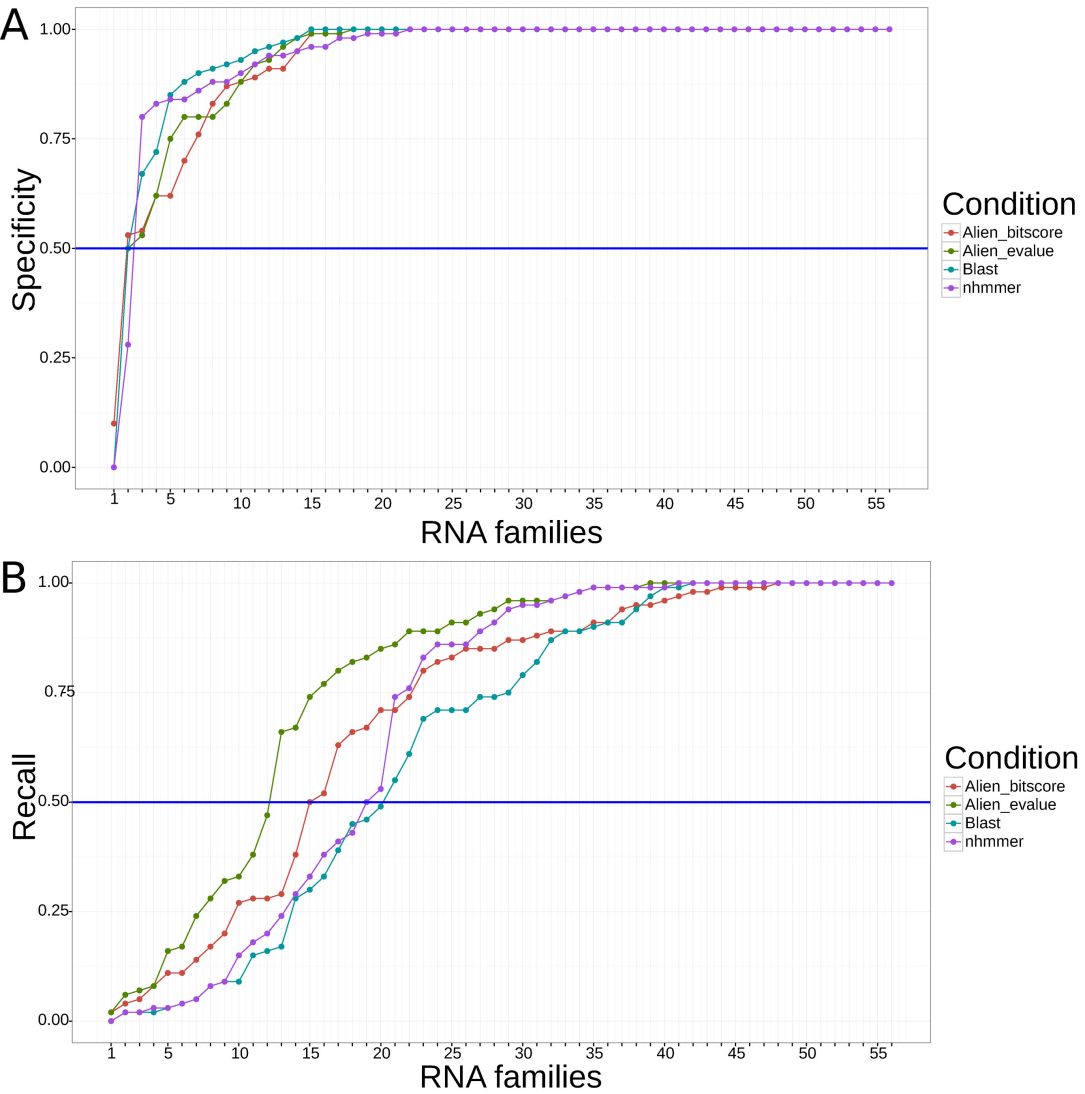
(**A**) Specificity of RNAlien homology search. The plot shows the fraction of homologs predicted by RNAlien that are recognized by the original Rfam model. In 55 of 56 cases (98%), at least half of the sequences collected by RNAlien are recognized as belonging to the Rfam model. In 35 (62%) families all sequences included by RNAlien are recognized as belonging to the Rfam model. (**B**) Recall of RNAlien models on Rfam sequences. We show the fraction of Rfam seed sequences recognized by the RNAlien model. In 44 of 56 cases (78%) at least half the sequences in the Rfam seed alignment are correctly recognized by the RNAlien model.

A total of 55 out of 56 families (∼98%) exhibit specificity >50%, meaning that more than 50% of their sequences are recognized by the original Rfam model as family member (see Figure 2). BLAST and nhmmer achieved a slightly higher specificity than RNAlien.

In 44 of 56 cases (78%), more than 50% of the Rfam seed sequences could be categorized by the RNAlien model as a family member (see Figure 2). RNAlien has higher recall than BLAST and nhmmer.

RNA families where RNAlien performs well in terms of specificity and recall are not necessarily the same. We therefore used the minimum of recall and specificity to classify successful and poor reconstructions.

**Figure 3.**
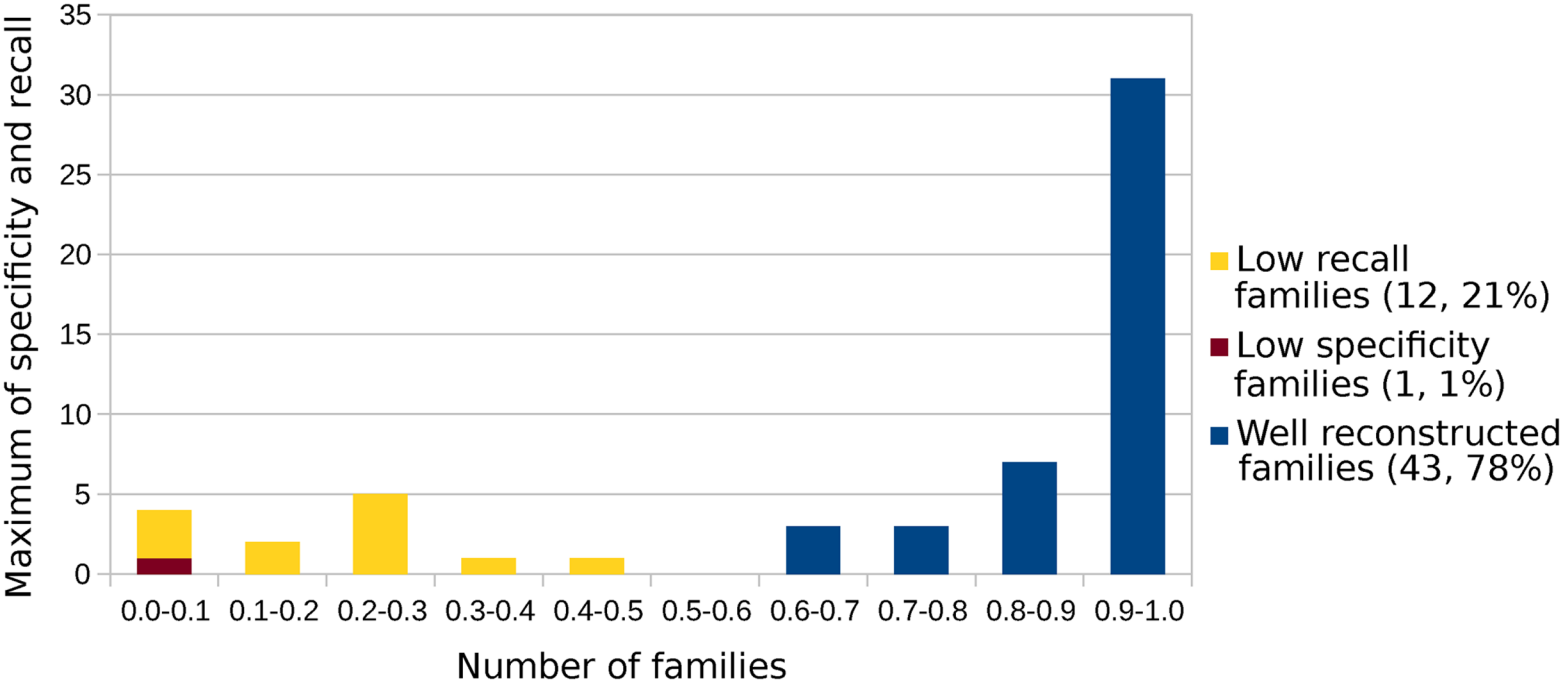
Family groups. To test our method, 56 Rfam family models with known structure were reconstructed by RNAlien from the first sequence picked from the family seed sequences. This plot shows the minimum of specificity and recall of all 56 reconstructed families. A total of 43 (∼78%) families achieve a specificity and recall ≥0.5 and are referred to as group *Well reconstructed families*. Only the *FMN* family where the Rfam model detected less than 50% of the sequences collected by RNAlien (Specificity) is in the *Low specificity – families* group. A total of 12 reconstructed families (∼21%) where the Alien model detected less than 50% of Rfam model seed sequences (Recall) are grouped in *Low recall – families*.

As shown in Figure 3, 43 reconstructions (∼78%) achieved both recall and specificity ≥50% and were categorized as *well reconstructed* families. In the *low recall* (recall < 50%) group 12 cases (∼21%) still had specificity higher than 50%, indicating that RNAlien only found a subgroup of the Rfam family. The *low specificity* (specificity < 50%) group, consisting only of the *FMN* family, had recall above 50%. This indicates that RNAlien sometimes reports false positives.

The *Low specificity* (*FMN*) and *Low recall – families* groups (*Intron_gpI, Intron_gpII, Histone3, mir-689, crcB, c-di-GMP-II, THF, tRNA-Sec, Protozoa_SRP, group-II-D1D4-1*) are of special interest to understand problems in the model construction process. The construction processes with sub-optimal results will be discussed in the following.

The *FMN* family models the flavin mononucleotide riboswitch and the reconstructed model recovers nearly all seed sequences of the Rfam model. However, during the construction process more and more divergent hits are collected until the model becomes unspecific. In this case low specificity is the result of an uninformative start sequence that is too short and exhibits only simple structure.

RNAlien does only recover about 47% of the Rfam seed alignment sequences for the *tRNA* family, with but these with high specificity. The family is too diverse for RNAlien to find all potential members.

The same applies to the Protozoa_SRP family which features related families for metazoa, as well as protozoa and to a set of other families (*Histone3, mir-689, crcB, c-di-GMP-II, THF, tRNA-Sec, group-II-D1D4-1, IRE_II* ).

The *Intron_gpI* and *Intron_gpII* represents self splicing ribozymes that can be found in eukarya, bacteria and viruses. The *Intron_gpI*RNA features nine paired regions which are grouped in two domains, of which only the second one is featured in the Rfam model. The family is characterized by frequent variable length insertions in the loop regions.

The Rfam curators overcame this problem by manually adding biologically reasonable gaps in the seed alignment, thus reducing the cost of insertions. Moreover, the initial sequence selected for RNAlien is a viral sequence that is isolated both in terms of taxonomy and similarity with regard to the bulk of the family.

As expected, we observe among the low recall families, complex RNAs, such as group I introns, that exhibit large variation in length and would present challenges even for human experts.

### Secondary structure comparison

We compared consensus secondary structures between the annotated structure for Rfam families with known 3D-structure and corresponding RNAlien alignment consensus structure. A base-pair distance, as computed by RNAdistance (31) was used for the comparison.

RNA structure distances are most meaningful when structures for sequences of equal length are compared. Both the seed alignment and the final RNAlien alignment share at least the sequence used as input for RNAlien. We processed both consensus structures before the comparison by removing all positions that map to gaps for the shared sequence. Basepairs that lose their binding position in this manner are set to be unpaired.

**Figure 4.**
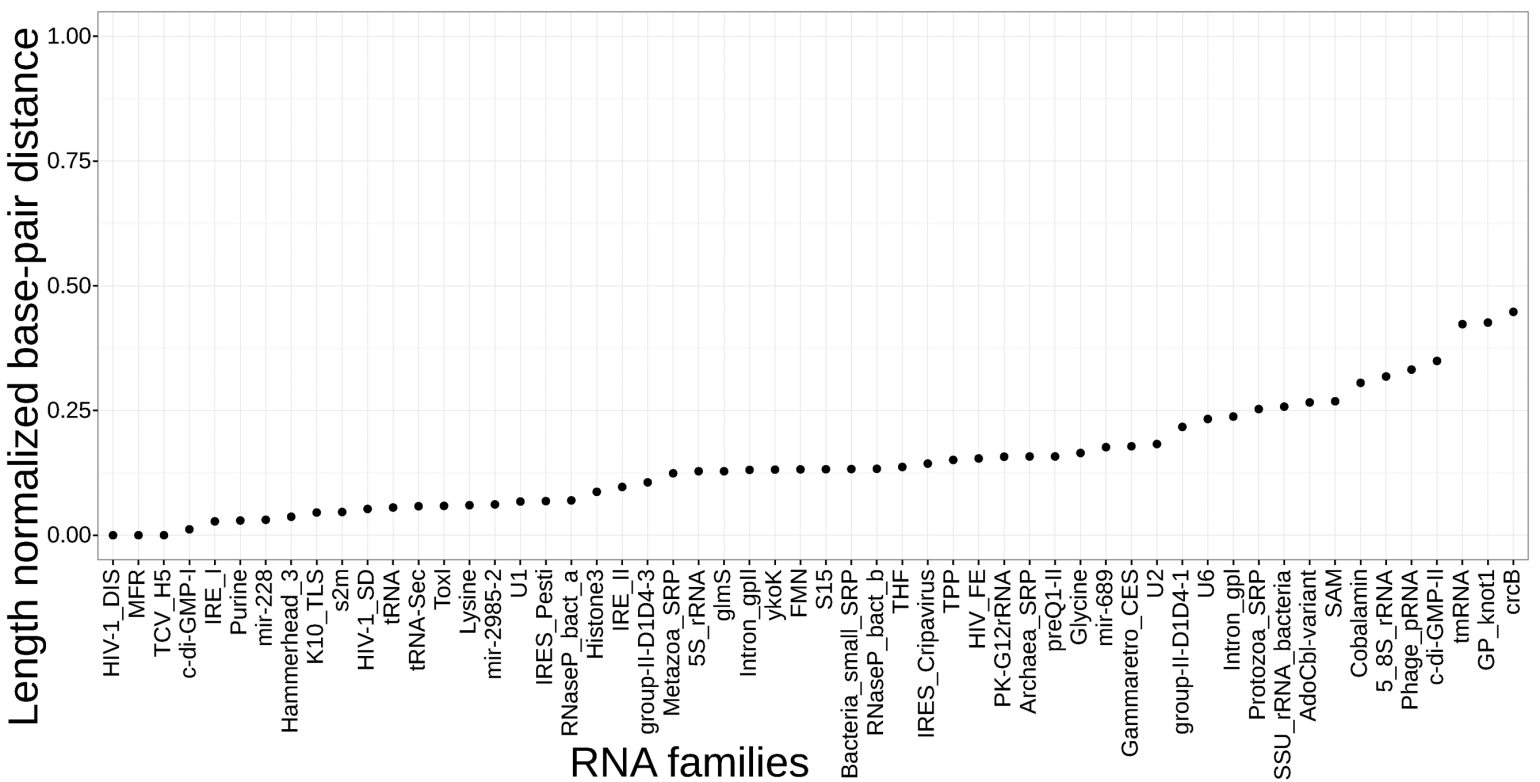
Length normalized secondary structure base-pair distances between the RNAlien consensus structure versus Rfam model consensus structure.

The resulting distances were normalized by the length of the sequence to make them comparable with each other, as shown in Figure 4. Constructions that achieved good specificity and recall in the benchmark do not necessarily have a low distance.

### Running times

The running times for constructing the 56 families in above benchmarks are shown in Supplementary Figure 7. The average running time (wall-clock time) with 20 cpu-cores was about 4 h, while the fastest construction with 40 min was the *archea_SRP* family model and the longest construction with 1 day 4 h was *Purine*.

### Negative control set

In the second test we applied RNAlien on a negative data set of 651 sequences consisting of 300 random, 34 ancestral repeat, 124 coding, 193 3΄ and 5΄-untranslated region sequences.

*Homo sapiens, Escherichia coli* and *Sulfolobus solfataricus* were used as organism of origin for 100 of the random sequences each. For none of these sequences was a second sequence search hit detected.

A total of 34 Dfam (32) families tagged as ancestral repeat a sequence was picked as input for RNAlien.The homology search for the sequences found multiple sequences but only one of the final RNAlien alignments was predicted by RNAz to be of structured RNA quality.

A total of 49 Coding sequences for *Homo sapiens*, 40 for *Escherichia coli* and 35 for *Sulfolobus solfataricus* were retrieved from Ensembl (28), RegulonDB (29) and NCBI genbank (27).

Each of the 124 sequences was used as input for RNAlien. In 24 of the cases homology search found no additional hits. The 100 remaining result alignments where evaluated using RNAcode, 75 of them were classified as protein coding with a P-value below 0.05. Of the remaining cases, 19 are neither predicted by RNAz to be RNA nor to be proteins by RNAcode, while 6 cases were identified to be structural RNA alignments.

This means that RNAlien can, in principle, provide meaningful output when given protein coding sequences as input, with the caveat that these sequences are often too long for folding algorithms to terminate in reasonable time and that the protein-specific features (e.g. reading frame) are not used.

If RNAlien received protein coding input, this is usually indicated by RNAcode in the evaluation step. Some of the constructed alignments were qualified as structured RNA by RNAz, which could be explained by conserved secondary structures that are contained in the reading frames of these alignments.

95 sequences from 5΄ and 3΄ untranslated regions from *Homo sapiens* and 98 from *Escherichia coli* were checked. *Escherichia coli* sequences are from egulonDB version 9.0 (29) *Homo sapiens* sequences are from Ensembl (28) (Release 84, GRCh38.p5), chromosome 2.

In 34 of the 193 cases no additional hits, meaning no hits that satisfied the filter criteria, were found by homology search. 30 of these cases were UTR input sequences from *Homo sapiens*. In 114 cases, the final RNAlien alignments were classified by RNAz as not structural RNA alignments, in 45 cases were classified as structured RNAs, of which 37 are in the 3΄-UTR of *E. coli*.

Finding structured RNA in UTRs is quite expected (33). One example are terminator hairpins in prokaryotic 3΄-UTRs. Possibly RNAlien could be also used to search for structural motifs in untranslated regions.

The full table of the negative data set results can be found in Supplementary Section F - Negative control set.

As can be observed from the results in Figures 2 and 3, our models do not recover all Rfam seed sequence sets with 100% sensitivity. This is, however, completely in line with our expectations. Putative homologs are collected solely via quite stringent BLAST hits, which limits the depth of a model to those homologs to be recovered using sequence-based searches only. Additional remote homologs can be discovered by running Infernal (4).

## WEB SERVER

RNA homology searches can be performed conveniently via the RNAlien web server. The server takes a fasta sequence and the organism of origin's name or NCBI taxonomy id as input. For each iteration step the server provides information on how many sequences have been collected so far and to which node of the taxonomic tree the search has progressed.

**Figure 5.**
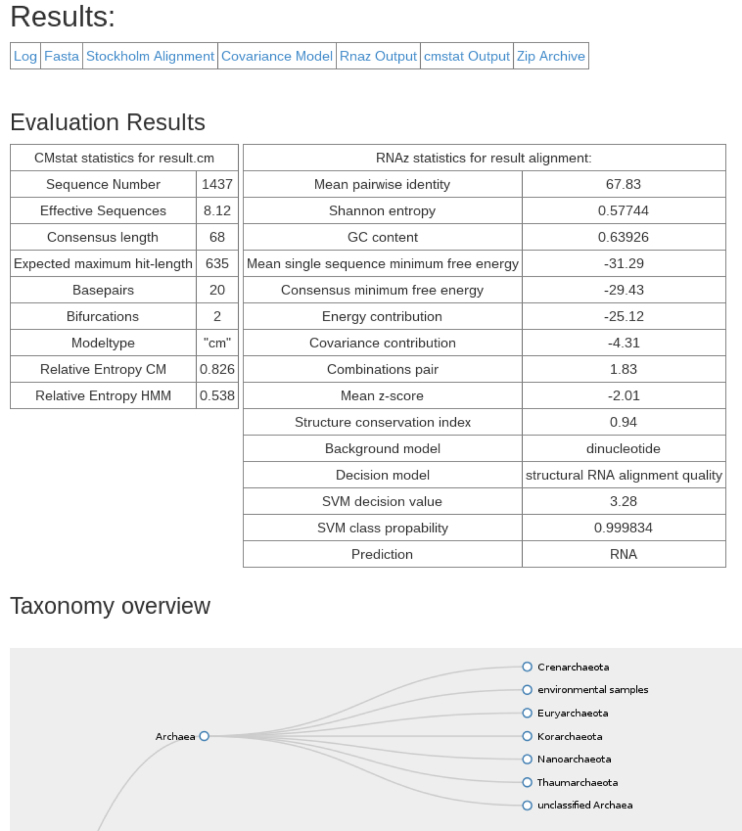
Result output of the RNAlien web service. The table at the top shows links to the final construction log, result sequences, alignment, covariance model, RNAz, cmstat and zip archive files. The zip archive contains all files of the construction for later reproducibility. The table in the center shows features computed for the result by cmstat, RNAz and RNAcode including the prediction if the result alignment is of structural RNA alignment quality. At the bottom a slice of the taxonomic tree, including all organisms that contained hits in the construction is shown. The tree is collapsible and zoom-able for better overview.

Upon completion the sequences, structural alignment and calibrated covariance model are available via download links (see Figure 5). All intermediate results are available as compressed archives for documentation and review of the results.

A key feature of the web server is a zoom-able and collapse-able taxonomic tree of the organisms where family members were found. The results of model evaluation, like the cmstat, RNAz and RNAcode output are summarized in a table.

The final covariance model can be directly passed on to the CMCompare web service (34,35) which compares it to all RNA family models in the Rfam database. This allows to find related families, or even an alternative pre-existing family model for the newly constructed model.

## CONCLUSION

With RNAlien we provide an automated pipeline for RNA homology search. Starting from a single sequence, a combined sequence-structure alignment is constructed. Sequences are collected from an ever-wider search within the phylogeny of the starting sequence, with the goal of producing a family of phylogenetically diverse members. The resulting family comes complete with a set of statistical predictors of quality, and a covariance model for further searches.

These results show that our method does indeed produce models that may serve as initial seed models for further investigation. The resulting alignment could also be used as input for iteratively expanding input seed alignments via multiple rounds (14).

However there are RNA families (10–13) for which an automated approach can only partially succeed, because the RNAs exhibit large variation in length and structure. Here, the use of contextual information, like promoter binding sites and other expert knowledge can help.

We point out that the dependency on BLAST could be easily dropped by directly using cmsearch for candidate search. While this could improve sensitivity, it would incur much higher computational cost, especially when scanning eukaryotic genomes. In the future we plan to add candidate search via nhmmer, speed up the pipeline by modifying model calibration and expand the construction process to include alternative model concepts (36).

Together with the web server, RNAlien provides a completely automated and easy to use method to construct initial structured RNA family models, based on a single initial sequence. This in turn considerably reduces the workload of an investigation into a novel sequence whose pedigree is unknown.

## Supplementary Material

Supplementary Data
