# Supplementary material for: RNAlien – Unsupervised RNA family model construction
Source: Nucleic Acids Res. 2016 Jun 21;44(17):8433–41. doi: 10.1093/nar/gkw558 (PMC5041467; doi:10.1093/nar/gkw558)
Supplement: Supplementary Data [file gkw558_supp.pdf]

RNAlien - Unsupervised RNA family model construction - Supplement

Florian Eggenhofer[1,2], Ivo L. Hofacker[1,3] and Christian Höner zu Siederdissen[4,1,5,]

[1] Institute for Theoretical Chemistry, University of Vienna, Währingerstrasse 17,A-1090 Vienna, Austria
[2] Bioinformatics Group, Department of Computer Science University of Freiburg, Georges-Köhler-Allee ,79110 Freiburg, Germany
[3] Bioinformatics and Computational Biology research group, University of Vienna, Währingerstrasse 17,A-1090 Vienna, Austria
[4] Bioinformatics Group, Department of Computer Science, University of Leipzig, D-04107 Leipzig
[5] Interdisciplinary Center for Bioinformatics, University of Leipzig, Härtelstraße 16-18, D-04107 Leipzig, Germany

Table of Contents

RNAlien - Unsupervised RNA family model construction - Supplement .. i
Florian Eggenhofer, Ivo L. Hofacker and Christian Höner zu Siederdissen
A `RNAlien` detailed flowchart ... iii
B Implementation Details ... iv
B.1 Initial model construction ... iv
Search: ... iv
Filtering hits: ... iv
Model Construction: ... vi
Select Queries: ... vi
B.2 Model expansion ... vii
Search: ... vii
Filtering hits: ... vii
Model Construction: ... viii
Select Queries: ... ix
B.3 Model finalization: ... ix
Search, Filter: ... x
Reevaluation of potential candidates: ... x
Modelconstruction: ... x
B.4 Model evaluation ... x
B.5 Blast hit extension ... xi
C Rfam RNA families with known structure ... xii
D Diverse Rfam RNA families benchmark set ... xvi
E Negative control set ... xxiii
E.1 Random sequences ... xxiii
E.2 Ancestral repeats ... xxiii
E.3 Coding sequences ... xxiii
E.4 UTR regions ... xxiii

A `RNAlien` detailed flowchart

Detailed flowchart representation of the RNAlien program flow.

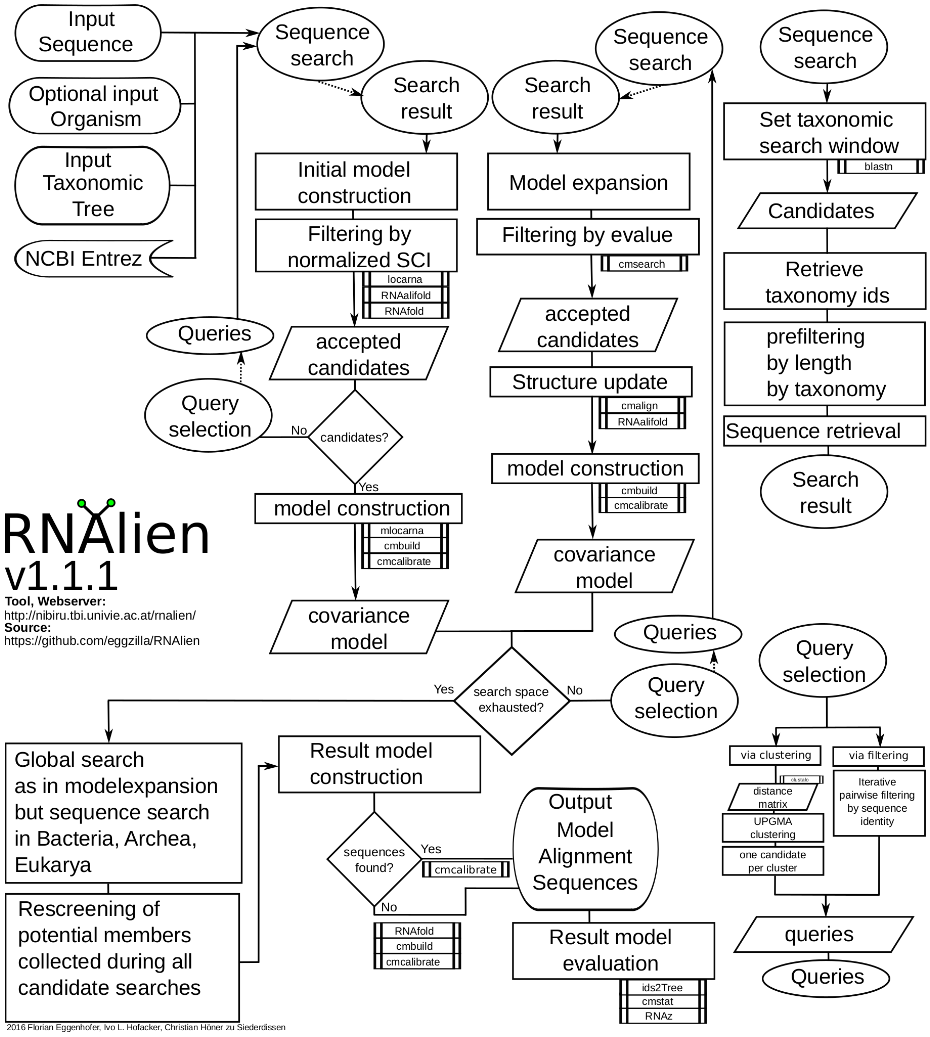

Fig. 1. Detailed program flow chart for RNAlien program.

B Implementation Details

RNAlien depends on several external tools and interfaces, which are listed in this section. System and function calls are included with their parameters and highlighted in italic. A starting point in the taxonomic tree is set, either specified by the input NCBI taxonomy id, or by running a nucleotide BLAST search via the NCBI REST interface and selecting the organism of the best hit. The model construction process starts at this organism and performs a initial model construction step. RNAlien retrieves the taxonomic lineage of starting organism from the NCBI ENTREZ REST interface. After each of these steps RNAlien proceeds to the taxnomic parent of the current taxonomic node. If a model was already constructed in a previous step then a model expansion step is performed, otherwise a initial model construction is reattemped. Once the root of the taxonomic tree has been reached model expansion stops and the model finalization step is performed.

B.1 Initial model construction

RNAlien tries to establish an inital set of sequences related to the input sequence, that serve as seed for further expansion of the model.

Search: Candidate search is performed via the nucleotide BLAST REST interface which returns a list of hits. The organisms to be searched are restricted by the current taxononomic node of the step in two ways. To avoid overenrichment of sequences similar to included ones, already visited organisms are excluded. Only organisms that are are associated with children of the current node are searched. For example if the current taxonomic position is *Enterobacteriacea* and the previous node was *Enterobacter* all other organisms that belong to *Enterobacteriacea* excluding *Enterobacter* are searched.
Summary of NCBI BLAST REST function call (one for each query, all other parameters default):

blastHTTP

parameter	*value*
program	blastn
database	nt
querySequence	currentsequence
hitlistSize	5000
e-value	0.001
uppertaxonomylimit	currenttaxonomyid
lowertaxonomylimit	previoustaxonomyid

Filtering hits: BLAST hits are filtered by consecutively by following criteria:
Hit has to achive over 80% coverage of the query sequence.

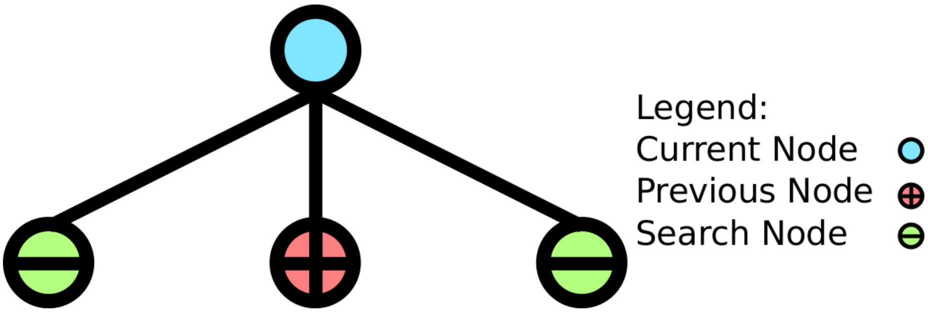

Fig. 2. Organisms used for candidate search are determined as follows. All organisms and their corresponding genomes that are associated with the currently selected position in the taxonomic tree are used for searching. Excepted from this are organisms that have already been searched in previous rounds.

The hit must not exceed query length by factor of three.
Similarity of the hit to the query must be under 99%.
The remaining hits are expanded to query length as explained in subsection B.5. The gene id contained in the `BLAST` result and the expanded coordinates are used to retrieve nucleotide sequence from the Entrez REST interface. The sequences are filtered by normalized structure conservation index (nSCI). To compute the nSCI for each candidate sequence we need the mimimum free secondary structure folding energy (MFE) of the candidate and the input sequence which is computed with RNAfold.

**RNAfold**	
parameter	*value*
−−noPS	
inputfilePath	fastaFilePath
outputFilepath	foldFilePath

Furthermore the structure conservation index and the sequence identity of the candidate and input sequence are required. The candidate sequence is pair-wise aligned with free end-gap setting (semi-globally) to the input sequence. For each of these alignments the structure conservation index SCI is computed via RNAalifold.

**locarna**	
parameter	*value*
−−write-structure	
−−free-endgaps=+ + −−	
−−clustal	clustalFormatFilePath
inputFilepath1	inputFastaFilePath
inputFilepath2	inputFastaFilePath
outputFilepath	locarnaFilePath

**RNAalifold**	
parameter	*value*
inputFilepath	clustalFormatFilePath
outputFilepath	aliFoldFilePath

The sequence identity is computed via levenstein distance with following edit costs (delete,insert,substitution,transposition)=1. Candidate sequences are accepted for model construction if their nSCI exceeds one.

Model Construction: Candidate sequences that passed the nSCI filter are then used to build the inital model together with the input sequence. The sequences are structually aligned with mlocarna.

**mlocarna**	
parameter	*value*
inputFilepath	inputFastaFilePath
outputFilepath	mlocarnaFilePath

`cmbuild` is applied to the resulting structural stockholm alignment to construct a covariance model.

**cmbuild**	
parameter	*value*
−−refine	
inputModelFilepath	cmFilePath
inputAlignmentFilepath	stockholmAlignmentFilePath
outputLogFilepath	logFilePath

The covariance model is used in the model expansion rounds to filter candidates and is therefore calibrated with cmcalibrate. This step is very time-consuming but sped up by using nonstandard (`--beta` 10^{-4}) parameter. This affects the pre-filter steps of `cmsearch`, but not the final step where the sequence is aligned to the model via the CYK algorithm. Meaning that this increase in calibration speed reduces sensitivity but not specificity.

**cmcalibrate**	
parameter	*value*
−−beta 1E-4	
inputModelFilepath	cmFilePath
outputFilepath	mlocarnaFilePath

Select Queries: At the end of the round queries for the candidate search of the next round are selected. `RNAlien` features a filtering and a clustering based method of query selection.

Filtering based method is the default method and iteratively removes all entries from the list of collected sequences, that do not have at most 95% pairwise sequence identity. This method has less specificity and sensitivity in the benchmarks (see 5, 6), but it is faster and removes the dependency on **clustalo**.

Clustering based method can alternatively be used by suppling the $-m$ commandline switch with the value *clustering* to `RNAlien`.Clustal omega is used to compute a pairwise distance matrix of all collected sequences for clustering.

**clustalo**	
parameter	*value*
−−full	
−−distmat-out	matrixFilePath
−−infile	fastaFilePath
outputFilepath	clustaloFilePath

`RNAlien` clusters the sequences via *unweighted pair group method with arithmetic mean* (UPGMA) and then incrementally increases the cutoff distance until 5 clusters can be formed. If less than 5 seqences have been collected, then each of them will be used as query.

B.2 Model expansion

After a initial model has been constructed `RNAlien` enters into model expansion phase.

Search: Searching is performed as descibed in Initial model construction but with a relaxed e-value cutoff of 1 during the `BLAST` search.

**blastHTTP**	
parameter	*value*
program	blastn
database	nt
querySequence	currentsequence
hitlistSize	5000
e-value	1
uppertaxonomylimit	currenttaxonomyid
lowertaxonomylimit	previoustaxonomyid

Filtering hits: Filtering of `BLAST` hits and hit expansion is performed as described in Initial model construction.

Sequences are also retrieved via the NCBI Entrez REST interface but then filtered with a different approach. We use the calibrated covariance model of the previous round and apply it with cmsearch to the canidate sequences. Candidates are accepted into the growing model if their e-value is below 0.001 or as specified by the inputEvalueCutoff commandline argument.

To ensure a meaningful e-value cutoff we need to consider the size of the database. We reuse the size of the blast database the hit originates from.

The value is not by itself contained in the blast XML output, but all the parameters needed to compute it. The relationship of E-value and bitscore (Equation 3 adopted from [1]):

$$e = d * q * 2^{-b} \tag{1}$$

where d = databasesize
e = e-value
b = bitscore
q = querylength

We compute the database size in Mbases that was used for the blast search as follows, by rearranging the equation above:

$$d = (e * 2^{b})/q \tag{2}$$

where d = databasesize
e = e-value
b = bitscore
q = querylength

Candidates are accepted into the growing model if their cmsearch E-value is below 0.001 or as specified by the inputEvalueCutoff commandline argument

**cmsearch**	
parameter	*value*
−−notrunc	
-Z	databaseSize
-g	covarianceModelPath
inputFilepath	sequenceFilePath
outputFilepath	cmsearchFilePath

Model Construction: Candidates that were accepted by cmsearch and already collected sequences are structurally aligned with the covariance model of the previous round.

**cmalign**	
parameter	*value*
inputModelFilepath	cmFilePath
inputSequenceFilepath	fastaFilePath
outputAlignmentFilepath	stockholmAlignmentFilePath

As the secondary structure of the resulting stockholm alignment is not updated in this process, a consensus secondary structure of the new alignment is computed via RNAalifold, with settings specifically optimized to consider covariance contributions. The old consensus secondary structure is replaced with the new one in the alignment.

**RNAalifold**	
parameter	*value*
-r	
−−cfactor	
-Z	databaseSize
-g	covarianceModelPath
inputFilepath	sequenceFilePath
outputFilepath	cmsearchFilePath

cmbuild is used to construct a updated covariance model.

**cmbuild**	
parameter	*value*
−−refine	
inputModelFilepath	cmFilePath
inputAlignmentFilepath	stockholmAlignmentFilePath
outputLogFilepath	logFilePath

The model is calibrated with cmcalibrate for the following candidate search.

**cmcalibrate**	
parameter	*value*
−−beta 1E-4	
inputModelFilepath	cmFilePath
outputFilepath	mlocarnaFilePath

Select Queries: Search candidates for the next round are selected as described in Inital model construction.

B.3 Model finalization:

Model finalization serves to collect family members that could not be included in earlier rounds, because the model was too specific at that point and make the results availble for the user. First individual candidate searches are performed in Archea, Bacteria, and Eukaria or as specified by the taxonomyRestriction commandline argument. The results are pooled and then processed as described in model expansion. The resulting model is then used to reevaluate collected potential candidates. These sequences are filtered as described in modelexpansion and if accepted included into the model. This final model is then calibrated with

default options to make it immedately useable for further homology search by the user.

Search, Filter: as in modelexpansion for 3 kingdoms (Archea - taxid 2157, Bacteria - taxid 2, Eukaria - taxid 2759)
Modelconstruction as in Modelexpansion

Reevaluation of potential candidates: Filter like in Modelexpansion

Modelconstruction: as described above

**cmbuild**	
parameter	*value*
−−refine	
inputModelFilepath	cmFilePath
inputAlignmentFilepath	stockholmAlignmentFilePath
outputLogFilepath	logFilePath

Calibration is done without speedup by −−beta 1E-4 for the final model

**cmcalibrate**	
parameter	*value*
inputModelFilepath	cmFilePath
outputFilepath	mlocarnaFilePath

B.4 Model evaluation

In this step descriptors for the result files are computed. The covariance model is used as input for `cmstat`, which computes among other features the cm and hmm content of the model. `cmalign` is used to generate a `clustalw` format result alignment which is prefiltered by rnazSelectSeqs.pl (auxiliary script packaged with `RNAz`). This filtered alignment is used as input for `RNAz` set to use the decision model for structural alignments. The most relevant output of `RNAz` in this case is if it predicts the input to be structured RNA, which is a indicator for successful model constructions.

**cmalign**	
parameter	*value*
−−outformat=Clustal	
inputModelFilepath	cmFilePath
outputFilepath	mlocarnaFilePath

**rnazSelectSeqs.pl**	
parameter	*value*
inputFilepath	clustalFilePath
outputFilepath	selectedClustalFilePath

**RNAz**	
parameter	*value*
-l	
inputFilepath	selectedClustalFilePath
outputFilepath	rnazFilePath

**cmstat**	
parameter	*value*
-l	
inputFilepath	covarianceModelPath
outputFilepath	cmstatFilePath

B.5 Blast hit extension

RNAlien expands found `BLAST` hits to the query length if possible.

Same strand `BLAST` hit are extended as follows,

$$t = h - q$$
$$T = H + (L - Q)$$

$$s(t) = \begin{cases} t, & \text{if } t \geq 0 \\ 0, & \text{otherwise} \end{cases}$$

$$E(T) = \begin{cases} b, & \text{if } T \geq b \\ T, & \text{otherwise} \end{cases}$$

where h is the start coordinate of the hit, t is the extended start coordinate, q is the start coordinate of the hit on the query, H is the end coordinate of the hit, T is the extended endcoordinate, Q is the end cooridnate of the hit on the query, L is the length of the query sequence b is the length of the sequence the hit maps to s is the start coordinate of the extended sequence checked for being within the available coordinates of the hit sequence, E is the end coordinate of the extended sequence checked for being within the available coordinates of the hit sequence

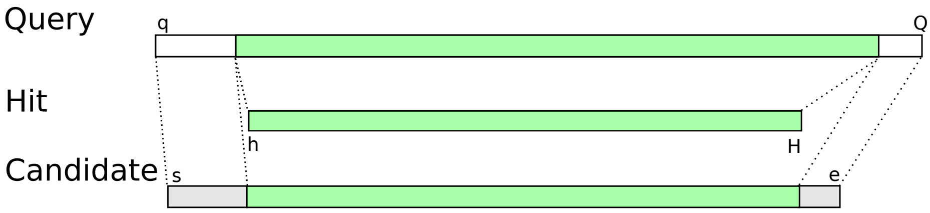

Fig. 3. Extension of `BLAST` hit and query on the same strand to query length, where h is the start coordinate of the hit, t is the extended start coordinate, q is the start coordinate of the hit on the query, H is the end coordinate of the hit, T is the extended endcoordinate, Q is the end cooridnate of the hit on the query, L is the length of the query sequence b is the length of the sequence the hit maps to s is the start coordinate of the extended sequence checked for being within the available coordinates of the hit sequence, E is the end coordinate of the extended sequence checked for being within the available coordinates of the hit sequence

Different Strand `BLAST` hit are extended as follows,

$$t = h + q$$
$$T = H - (L - Q)$$

$$s(t) = \begin{cases} b, & \text{if } t \geq b \\ t, & \text{otherwise} \end{cases}$$

$$e(T) = \begin{cases} T, & \text{if } T \geq 0 \\ 0, & \text{otherwise} \end{cases}$$

where h is the start coordinate of the hit,
t is the extended start coordinate,
q is the start coordinate of the hit on the query,
H is the end coordinate of the hit,
T is the extended endcoordinate,
Q is the end cooridnate of the hit on the query,
L is the length of the query sequence
b is the length of the sequence the hit maps to
s is the start coordinate of the extended sequence checked for being within the available coordinates of the hit sequence,
E is the end coordinate of the extended sequence checked for being within the available coordinates of the hit sequence

C Rfam RNA families with known structure

This section contains additional plots for the RNA families with known structure featured in the paper. The first 2 plots show the changes of specificity and

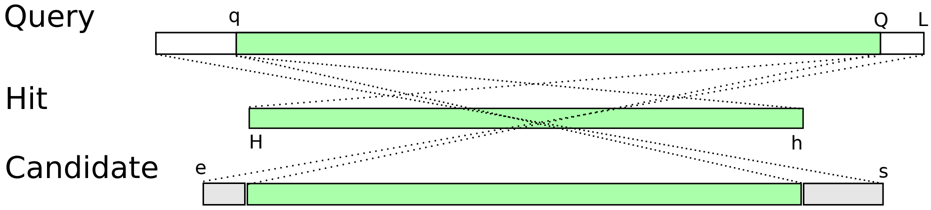

Fig. 4. Extension of BLAST hit and query on different strands to query length

sensitivity after subsequently applying the suggestions of the reviewers. The orginal version before the review was RNAlien 1.0.0, the one including all changes listed here has version 1.1.1.

Inclusion of paralogs and toggling of the refine switch for cmbuild were included first, this has improved both specificity, as well as recall. Additionally to this, we changed the method for selecting queries for searching candidates from clustering all collected sequences and picking one sequence per cluster to filtering all sequence that do not have a pairwise sequence identity of less then 95%.

While the specificity is slightly only lower, there is a decrease in specificity. Nevertheless we have selected the new query selection method as default, because it is substantially faster and it drops the dependency on clustal-omega.

Blast hits are now also checked for the hit to have at least 80% coverage of the query. This feature should have been included in RNAlien 1.0.0, but was faulty.

Query sequences submitted to blast can be softmasked with conservation information from /cmalign. This feature is not considered in the shown benchmarks, but can be activated via commandline switch.

All of the newly introduced features can be controlled via commandline switches, with exception of the cmbuild refinement.

The runtime of RNAlien for the structured RNA test set

Following is the table of sequences from the Rfam 12.0 seed alignments of families with known structure, that was used in the result section. The first sequence of the family was picked with the exception of sequences that are associated with metagenomic tax ids that could not be processed by the NCBI REST BLAST interface.

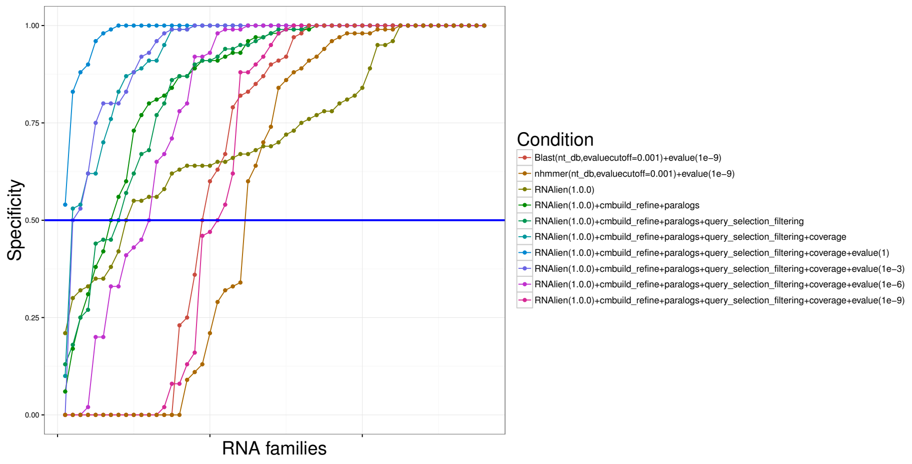

Fig. 5. Specificity for 56 RNA families with known 3D structure.

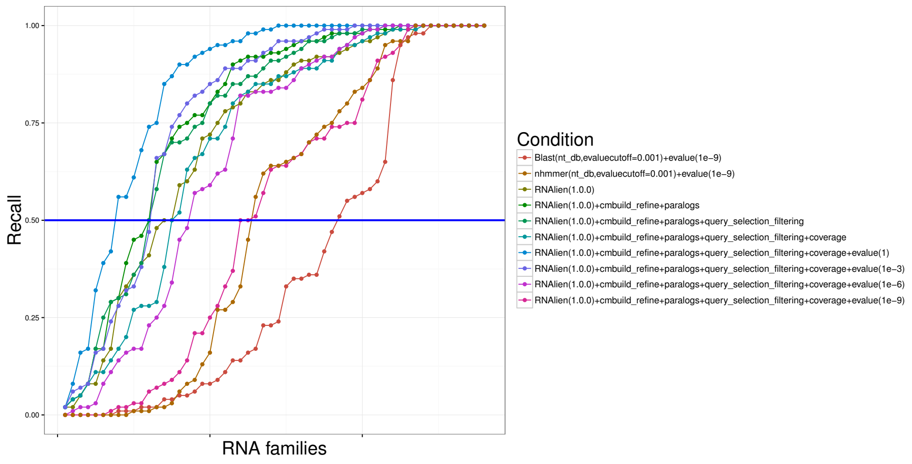

Fig. 6. Recall for 56 RNA families with known 3D structure.

Table 1: RNA families with known structure benchmark table. Column names A to N are placeholders for the following names: Specificity_Alien (=A) Sens_Alien (=B) Spec+paralogs+refine (=C) Sens+paralogs+refine (=D) Spec+filterings (=E) Sens+filtering (=F) Spec+coverage (=G) Sens+coverage (=H) Spec_evalue (=I) Sens_evalue (=J) Spec_nhmmer_evalue (=K) Sens_nhmmer_evalue (=L) Spec_blast_evalue (=M) Sens_blast_evalue (=N). The column names annotated with evalue were computed with a evalue cutoff of 1^{-3} and a databasesize of 10^9 bases per default, with the exception of families that can be found exclusively in prokaryotes and viruses.

**Rfam id**	**Rfam name**	**A**	**B**	**C**	**D**	**E**	**F**	**G**	**H**	**I**	**J**	**K**	**L**	**M**	**N**
5S_rRNA	RF00001	0.64	0.91	0.99	0.92	0.77	0.82	1	0.87	1	0.83	0.94	0.53	0.72	0.55

Continued on next page

Table 1 – continued from previous page

Rfam id	Rfam name	A	B	C	D	E	F	G	H	I	J	K	L	M	N
5_8S_rRNA	RF00002	0.95	0.9	1	0.9	1	0.85	1	0.85	1	0.89	0.96	0.95	1	0.74
U1	RF00003	0.58	0.88	0.97	1	0.99	1	1	1	1	0.99	0.86	0.99	1	0.75
U2	RF00004	0.62	0.99	0.89	0.98	0.99	0.99	0.99	0.95	0.99	0.96	0.84	0.99	0.96	0.89
tRNA	RF00005	0.77	0.48	1	0.75	1	0.7	1	0.63	0.75	0.47	0.9	0.15	1	0.04
Hammerhead_3	RF00008	1	0.63	1	0.74	1	0.74	1	0.74	1	0.74	1	0.74	1	0.74
RNaseP_bact_a	RF00010	0.56	1	0.93	0.98	0.94	0.98	1	1	1	1	0.98	1	1	1
RNaseP_bact_b	RF00011	0.55	1	0.99	1	1	1	1	1	1	1	0.99	1	0.91	1
Metazoa_SRP	RF00017	0.35	0.92	0.06	0.96	0.13	0.96	1	0.99	1	0.99	0.95	0.96	1	0.99
tmRNA	RF00023	0.65	0.92	0.98	0.93	0.98	0.92	0.99	0.88	0.99	0.91	0.98	0.95	0.98	0.61
U6	RF00026	0.64	0.83	0.98	0.83	0.99	0.82	1	0.82	1	0.8	0.88	0.89	0.93	0.71
Intron_gpI	RF00028	0.32	0.08	0.17	0.17	0.27	0.25	1	0.08	1	0.08	0.28	0.08	1	0.08
Intron_gpII	RF00029	0.75	0.41	0.31	0.65	0.92	0.58	0.89	0.2	0.92	0.16	0.84	0.2	1	0.09
Histone3	RF00032	1	0.02	1	0.02	1	0.02	1	0.02	0.5	0.02	1	0.02	1	0.02
IRE_I	RF00037	0.78	0.92	0.99	0.92	0.95	0.87	0.95	0.89	0.96	0.89	1	0.05	1	0.05
Phage_pRNA	RF00044	0.8	1	0.8	1	0.8	1	1	1	1	1	0.8	1	1	1
FMN	RF00050	0.21	0.79	0.6	1	0.18	1	0.1	0.66	1	1	1	1	0.85	1
TPP	RF00059	0.63	0.83	0.77	0.92	0.57	0.91	0.87	0.83	0.8	0.82	1	0.5	1	0.3
S15	RF00114	0.56	0.85	1	0.85	1	0.85	1	0.85	1	0.85	1	0.83	1	0.82
SAM	RF00162	0.55	0.72	0.81	0.99	0.67	0.98	0.76	0.91	0.98	1	0.99	0.99	1	0.79
s2m	RF00164	1	1	1	1	1	1	1	0.97	1	0.97	1	0.18	1	0.87
Purine	RF00167	0.66	0.71	1	1	1	1	1	0.99	1	1	1	0.99	1	0.94
Lysine	RF00168	0.82	0.94	1	0.98	1	0.89	1	0.85	1	0.94	1	0.98	1	0.45
Bacteria_small_SRP	RF00169	0.64	0.75	0.99	0.77	0.99	0.75	1	0.27	1	0.67	1	0.41	1	0.39
Cobalamin	RF00174	0.73	0.6	0.87	0.99	0.91	1	1	0.94	1	0.96	1	0.97	0.9	0.9
HIV-1_DIS	RF00175	1	0.99	1	0.99	1	0.99	1	0.99	1	1	1	0.91	1	0.91
SSU_rRNA_bacteria	RF00177	1	1	1	1	1	1	1	1	1	1	1	1	1	1
K10_TLS	RF00207	1	1	1	1	1	1	1	1	0.8	1	1	1	1	1
IRES_Pesti	RF00209	0.95	1	0.91	1	0.91	1	1	1	1	1	0.96	1	1	1
glmS	RF00234	0.69	1	0.97	1	1	1	1	0.89	1	0.89	1	1	1	0.89
Gammaretro_CES	RF00374	0.64	1	0.87	1	0.87	1	1	1	1	1	0.99	1	1	1
ykoK	RF00380	0.7	0.86	1	0.94	1	0.96	1	0.89	1	0.99	1	0.99	0.95	0.99
IRES_Cripavirus	RF00458	0.33	0.14	0.25	0.29	0.25	0.29	1	0.29	1	1	1	0.86	1	1
HIV_FE	RF00480	1	0.98	1	0.98	1	0.98	1	0.98	1	0.99	1	0.99	1	0.97
TCV_H5	RF00500	1	0.8	1	0.8	1	0.8	1	0.8	1	1	1	1	1	1
Glycine	RF00504	0.69	0.59	0.91	0.77	0.87	0.7	0.91	0.52	0.99	0.66	1	0.09	1	0.09
mir-228	RF00843	1	1	1	1	1	1	1	1	1	1	1	1	1	1
mir-689	RF00871	0.5	0.08	0.5	0.08	0.5	0.08	0.83	0.38	0.83	0.38	0.92	0.38	1	0.46
c-di-GMP-I	RF01051	0.81	0.97	1	0.97	0.94	0.94	1	0.96	1	0.98	1	0.76	1	0.69
preQ1-II	RF01054	0.67	0.93	1	0.93	1	0.93	1	0.71	1	0.93	1	0.86	1	0.71
GP_knot1	RF01073	0.96	0.86	0.96	0.71	0.96	0.71	0.91	0.71	0.93	0.86	1	0.43	0.88	0.71
PK-G12rRNA	RF01118	0.65	0.99	0.73	0.99	0.68	0.97	0.99	0.99	1	1	1	1	1	1
HIV-1_SD	RF01380	1	0.05	1	0.05	1	0.05	1	0.05	1	0.77	1	0	1	0

Continued on next page

Table 1 – continued from previous page

Rfam id	Rfam name	A	B	C	D	E	F	G	H	I	J	K	L	M	N
MFR	RF01510	1	0.33	1	0.67	1	0.67	1	0.67	1	1	1	1	0.67	1
AdoCbl-variant	RF01689	1	0.91	1	0.91	1	0.91	1	0.91	1	0.91	1	0.86	1	0.91
crcB	RF01734	0.84	0.36	0.93	0.45	1	0.36	0.88	0.28	0.88	0.32	1	0.03	1	0.03
c-di-GMP-II	RF01786	0.67	0.02	1	0.04	1	0.04	1	0.04	1	0.07	1	0.04	1	0.02
THF	RF01831	0.76	0.96	1	0.96	0.86	0.87	0.7	0.14	0.8	0.24	1	0.24	1	0.16
tRNA-Sec	RF01852	0.89	0.3	0.92	0.3	0.9	0.3	0.53	0.28	0.53	0.28	0.88	0.29	0.92	0.28
Protozoa_SRP	RF01856	0.72	0.39	0.91	0.39	0.95	0.39	1	0.11	1	0.33	1	0.33	1	0.33
Archaea_SRP	RF01857	0.35	0.96	0.82	0.96	0.45	0.96	1	0.87	1	0.96	1	1	1	0.15
group-II-D1D4-1	RF01998	0.38	0.5	0.38	0.46	0.45	0.31	0.62	0.11	0.62	0.06	0.83	0.02	0.5	0.02
group-II-D1D4-3	RF02001	0.3	0.78	1	0.98	1	0.99	1	0.98	1	0.96	0.94	0.94	0.97	0.49
mir-2985-2	RF02095	0.68	0.95	0.84	0.95	0.97	1	1	0.95	1	1	1	1	1	1
IRE_II	RF02253	0.42	0.17	0.42	0.17	0.44	0.17	0.54	0.17	0	0.17	0	0.03	0	0.17
ToxI	RF02519	0.78	0.5	0.56	0.5	0.62	0.5	0.62	0.5	1	1	1	1	1	1

D Diverse Rfam RNA families benchmark set

The Rfam database features following tags to group families: Cis-reg, frameshift_element, IRES, leader, riboswitch, thermoregulator, antisense, antitoxin, CRISPR, lncRNA, miRNA, ribozyme, rRNA, snRNA, snoRNA, CD-box, HACA-box, scaRNA, splicing, Gene, sRNA, tRNA, Intron.

To obtain a representative sample of Rfam families, for each of these tags the alphanumberically first 10 families (if avialable for that tag) were selected. As some families have multiple tags, the list was filtered to contain each family only once.

The benchmark was conducted in the same manner as for the families with known 3D structure. The plots show different combinations of e-value cutoffs and databasesizes. Without explicitly setting the database size cmsearch uses twice the sequence length (forward/backward strand).

The setting compareable to the one used for the structured dataset is diverse(ev-1e-3,db-1e-9), meaning a cmsearch e-value cutoff of 1e-3 and a databasesize of 10^9 bases in general and 10^6 bases for bacterial and viral RNA families.

The result with compareable settings to the structured dataset has 191 of 192 cases (99%) with at least half of the sequences collected by `RNAlien` are recognized as belonging to the `Rfam` model. In 170 (89%) families all sequences included by RNAlien are recognized as belonging to the `Rfam` model

In of 163 cases (85%) at least half the sequences in the `Rfam` seed alignment are correctly recognized by the `RNAlien` model. In 123 of 191 cases (64%) all sequences in the `Rfam` seed alignment are correctly recognized by the `RNAlien` model.

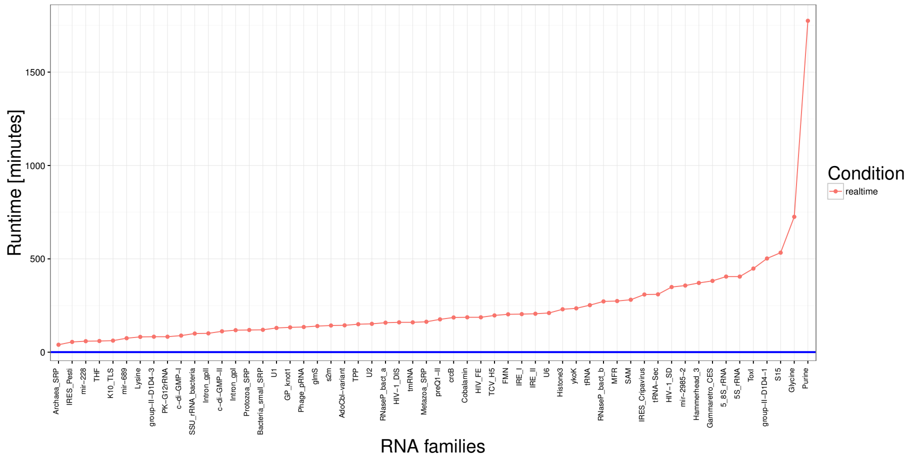

Fig. 7. Alien program runtime in minutes for structured families

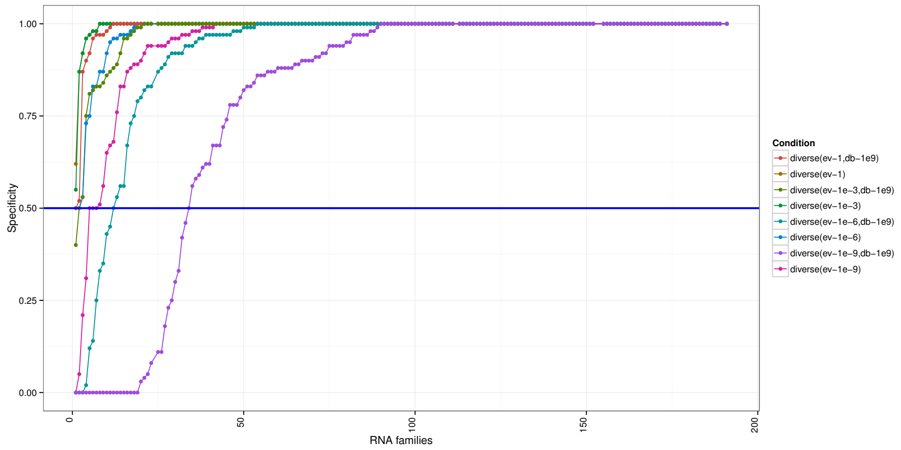

Fig. 8. Specificity of `RNAlien` homology search. The plot shows the fraction of homologs predicted by `RNAlien` that are recognized by the original `Rfam` model. The legend indicates the e-evalue cutoff (ev-) and the database size used. The e-evalue cutoffs start at 1 and are made stricter in 1e-3 steps up to 1e-9. The result with compareable settings to the structured dataset has 191 of 192 cases (99%) with at least half of the sequences collected by `RNAlien` are recognized as belonging to the `Rfam` model. In 170 (89%) families all sequences included by RNAlien are recognized as belonging to the `Rfam` model

Following is the table of familes from the Rfam 12.0 used in the as a second benchmark set.

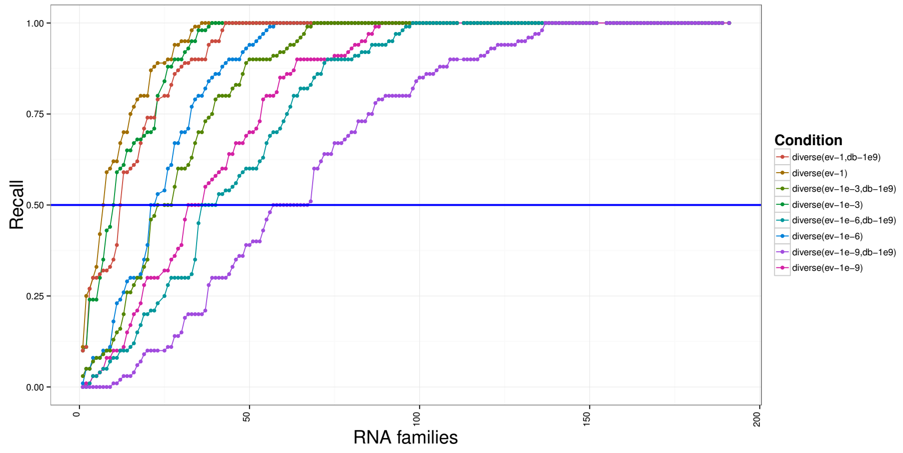

Fig. 9. Recall for 191 RNA families, selected up to 10 for each family tag. To test our method, sRNA Rfam family models were reconstructed by `RNAlien` from a random sequence picked from the family seed sequences. This plot shows how many `Rfam` seed sequences are recognized by the reconstructed `RNAlien` model using the model gathering score (used by `Rfam` to establish full models). In of 163 cases (85%) at least half the sequences in the `Rfam` seed alignment are correctly recognized by the `RNAlien` model. In 123 of 191 cases (64%) all sequences in the `Rfam` seed alignment are correctly recognized by the `RNAlien` model.

Table 2: Diverse RNA families benchmark set. Column names A to D are placeholders for following names: Specificty_evalue_1 (=A) Sensitivity_evalue_1 (=B) Specificity_evalue_1e-3 (=C) Sensitivity_evalue_1e-3 (=D) Specificity_evalue_1e-6 (=E) Sensitivity_evalue_1e-6 (=F) Specificity_evalue_1e-9 (=G) Sensitivity_evalue_1e-9 (=H)

**Rfam name**	**Rfam id**	**A**	**B**	**C**	**D**	**E**	**F**	**G**	**H**
5S_rRNA	RF00001	1	0.88	1	0.75	0.97	0.54	0.58	0.48
5_8S_rRNA	RF00002	1	0.89	1	0.82	1	0.75	1	0.69
U1	RF00003	1	1	1	1	0.98	0.98	0.94	0.94
U2	RF00004	1	1	0.99	0.96	0.92	0.83	0.88	0.75
tRNA	RF00005	1	0.61	0.75	0.47	0.02	0.25	0	0.03
RNaseP_nuc	RF00009	1	0.11	1	0.09	1	0.08	1	0.07
RNaseP_bact_b	RF00011	1	1	1	1	1	1	1	1
U4	RF00015	0.97	0.74	0.96	0.55	0.94	0.12	0.9	0.06
Y_RNA	RF00019	0.52	0.59	0.4	0.33	0.12	0.31	0.05	0.31
U5	RF00020	1	0.9	1	0.61	0.97	0.21	0.78	0.09
U6	RF00026	1	0.94	1	0.8	0.99	0.63	0.88	0.51
PrfA	RF00038	1	1	1	1	1	1	1	1
CopA	RF00042	1	1	1	1	1	1	0.97	0.97
FMN	RF00050	1	1	1	1	0.99	0.99	0.8	0.78

Continued on next page

Table 2 – continued from previous page

Rfam name	Rfam id	A	B	C	D	E	F	G	H
TPP	RF00059	1	0.95	1	0.83	1	0.53	0.67	0.39
U7	RF00066	1	0.71	0.99	0.63	0.79	0.53	0.18	0.39
SNORD29	RF00070	1	0.3	1	0.3	1	0.3	0	0.3
mir-29	RF00074	0.96	1	0.82	0.6	0.56	0.6	0.11	0.4
RNAI	RF00106	1	1	1	1	0.97	1	0.91	0.9
SIB_RNA	RF00113	1	1	1	1	1	1	1	1
snoZ159	RF00160	1	0.3	1	0.1	0.33	0.1	0	0.1
Hammerhead_1	RF00163	1	0.31	1	0.1	0	0.03	0	0.03
Purine	RF00167	1	0.89	1	0.83	1	0.55	1	0.14
SSU_rRNA_bacteria	RF00177	1	1	1	1	1	1	1	1
IRES_Bag1	RF00222	1	1	1	1	1	1	1	1
glmS	RF00234	1	1	1	0.89	1	0.89	1	0.33
ctRNA_pGA1	RF00236	1	1	1	1	1	0.6	1	0.2
RNA-OUT	RF00240	1	1	1	1	1	1	1	1
ctRNA_pT181	RF00242	1	1	1	0.94	1	0.69	1	0.62
IRES_L-myc	RF00261	1	1	1	1	0.82	1	0.23	1
SCARNA18	RF00283	1	1	1	1	1	1	0.86	0.95
SCARNA8	RF00286	1	1	1	1	1	1	1	1
snoR86	RF00303	1	1	1	1	1	1	0.88	1
snoZ157	RF00333	1	1	1	1	0.94	0.9	0.83	0.8
snoR60	RF00339	1	1	0.96	1	0.96	1	0.72	0.9
ydaO-yuaA	RF00379	1	1	1	0.99	1	0.94	0.9	0.84
Antizyme_FSE	RF00381	1	1	1	0.92	0.99	0.62	0.91	0.46
Pox_AX_element	RF00384	1	1	1	1	1	1	1	1
IBV_D-RNA	RF00385	1	1	1	1	1	1	1	0.9
SNORA30	RF00415	1	1	1	0.73	1	0.73	1	0.64
SCARNA24	RF00422	1	1	1	1	1	1	1	0.87
SCARNA15	RF00426	1	1	1	1	1	0.77	0.88	0.64
SCARNA23	RF00427	1	1	1	1	1	1	1	0.94
Hsp90_CRE	RF00433	1	1	1	1	1	1	1	1
ROSE	RF00435	1	1	1	0.46	1	0.15	0	0.15
IRES_HIF1	RF00449	1	1	1	1	1	0.94	0.87	0.88
IRES_mnt	RF00457	1	1	1	1	1	1	1	0.95
HCV_SLVII	RF00468	1	1	1	1	1	1	1	0.91
HCV_SLIV	RF00469	1	1	1	1	1	1	1	0.94
SCARNA6	RF00478	1	1	1	0.94	1	0.94	1	0.94
HIV_FE	RF00480	1	1	1	0.99	1	0.99	0.92	0.86
IRES_Cx43	RF00487	1	1	1	1	0.98	1	0.83	0.93
U1_yeast	RF00488	1	1	1	1	1	1	1	0.8
ctRNA_p42d	RF00489	0.97	1	0.81	0.9	0.53	0.6	0.03	0.2
IRES_Hsp70	RF00495	1	0.86	1	0.86	1	0.86	1	0.86
RNAIII	RF00503	1	1	1	1	0.56	1	0.56	1
Thr_leader	RF00506	1	1	1	1	1	1	0.95	0.96
snosnR64	RF00509	1	1	1	1	0.91	1	0.82	0.9
Leu_leader	RF00512	1	1	1	1	1	1	1	1
Trp_leader	RF00513	1	0.95	1	0.91	0.98	0.59	0.78	0.36
His_leader	RF00514	1	1	1	1	1	0.97	0.99	0.79
Continued on next page									

Table 2 – continued from previous page

Rfam name	Rfam id	A	B	C	D	E	F	G	H
PreQ1	RF00522	1	0.74	0.84	0.26	0.14	0.17	0	0.14
Flavivirus_DB	RF00525	1	1	1	1	1	0.92	1	0.68
snoMe28S-G3255	RF00527	1	1	1	0.5	1	0.3	0	0.1
IRES_TrkB	RF00547	1	1	1	1	1	0.94	1	0.94
IRES_c-sis	RF00549	1	1	1	1	1	1	1	1
L13_leader	RF00555	1	0.35	1	0.35	1	0.35	0.95	0.35
L19_leader	RF00556	1	1	1	0.6	0.88	0.2	0	0.2
L20_leader	RF00558	1	0.79	1	0.3	1	0.21	0.94	0.21
L21_leader	RF00559	1	0.87	1	0.74	0.96	0.45	0.46	0.11
SCARNA3	RF00565	1	1	1	1	1	1	1	0.96
SCARNA14	RF00582	1	1	1	1	1	1	1	0.86
CoTC_ribozyme	RF00621	1	1	1	1	1	1	1	0.9
CPEB3_ribozyme	RF00622	1	1	1	1	1	0.92	1	0.67
P1	RF00623	1	1	1	1	1	0.86	1	0.5
P24	RF00629	1	1	1	1	1	1	1	1
MIR169_2	RF00645	1	0.32	1	0.07	1	0.03	1	0.03
MIR168	RF00677	1	1	1	1	0.97	0.9	0.87	0.6
MIR162_2	RF00742	1	1	1	0.9	0.75	0.1	0.25	0.1
mir-342	RF00760	1	1	1	1	1	1	1	1
mir-541	RF00777	1	1	1	0.9	0.92	0.9	0.62	0.9
mir-1255	RF00994	0.99	0.9	0.86	0.9	0.45	0.9	0.11	0.1
WLE3	RF01046	1	1	1	0.7	1	0.7	1	0.6
Sacc_telomerase	RF01050	1	1	1	1	1	1	1	1
preQ1-II	RF01054	1	1	1	0.93	1	0.71	1	0.64
MOCO_RNA_motif	RF01055	1	0.33	1	0.13	1	0.07	1	0.02
RF_site2	RF01076	1	1	0.83	1	0.67	1	0.67	1
RF_site3	RF01079	1	1	1	1	1	1	1	0.5
RF_site5	RF01093	1	1	1	1	1	0.58	0.9	0.5
RF_site9	RF01098	1	1	1	1	1	1	1	1
PK-G12rRNA	RF01118	1	1	1	1	1	1	1	1
snoZ30a	RF01196	1	1	1	1	1	1	1	1
snoR103	RF01213	0.87	1	0.87	1	0.87	0.82	0.87	0.73
snoR442	RF01232	1	1	1	1	0.25	0.7	0	0.1
snR161	RF01237	1	1	1	0.9	1	0.9	1	0.5
snR36	RF01242	1	1	1	1	1	1	1	1
snR8	RF01248	1	1	1	1	1	0.91	1	0.91
snR190	RF01249	1	1	1	1	1	0.8	1	0.8
snR5	RF01252	1	1	1	1	1	0.82	1	0.82
snR35	RF01255	1	1	1	1	1	1	1	1
snR191	RF01263	1	1	1	1	1	1	1	1
SCARNA2	RF01268	1	0.95	1	0.95	1	0.95	1	0.95
snoR2	RF01292	1	1	1	1	1	1	1	1
SCARNA7	RF01295	1	1	1	1	1	0.94	1	0.94
AHBV_epsilon	RF01313	1	1	1	1	1	1	0.88	1
CRISPR-DR2	RF01315	1	0.74	1	0.05	0	0.05	0	0
CRISPR-DR3	RF01316	0.5	0.1	0.5	0.05	0	0	0	0
CRISPR-DR5	RF01318	1	1	1	0.08	1	0.08	0	0

Continued on next page

Table 2 – continued from previous page

Rfam name	Rfam id	A	B	C	D	E	F	G	H
CRISPR-DR7	RF01320	1	0.9	1	0.2	1	0.1	0	0
CRISPR-DR35	RF01345	1	1	1	1	1	1	1	0
CRISPR-DR53	RF01366	1	1	1	1	1	1	1	0
CRISPR-DR60	RF01373	1	1	1	1	1	0.5	0	0.5
CRISPR-DR61	RF01374	1	1	0.83	1	0.83	1	0	0.5
CRISPR-DR65	RF01378	1	1	1	1	1	1	0	0
isrA	RF01385	1	1	1	1	0.97	1	0.97	1
istR	RF01400	1	1	1	1	1	1	0.97	1
NrrF	RF01416	1	1	1	1	1	1	1	1
IsrR	RF01419	1	0.98	1	0.97	1	0.91	1	0.88
VrrA	RF01456	1	1	1	1	0.95	1	0.84	1
Afu_300	RF01509	1	1	1	1	1	1	0.61	0.5
MFR	RF01510	1	1	1	1	1	1	1	0.67
Afu_309	RF01512	1	1	1	1	1	1	1	1
Dictyostelium_SRP	RF01570	1	1	1	1	1	1	1	1
RNase_P	RF01577	1	1	1	1	1	1	1	1
AdoCbl-variant	RF01689	1	0.62	1	0.03	1	0.01	1	0.01
Lnt	RF01711	1	0.9	1	0.8	1	0.3	0	0.3
cspA	RF01766	1	1	1	1	1	1	1	1
SMK_box_riboswitch	RF01767	1	0.6	1	0.08	1	0.04	1	0.04
rnk_leader	RF01771	0.97	1	0.97	1	0.97	0.85	0.97	0.85
RatA	RF01776	1	1	0.88	1	0.35	0.56	0.04	0.5
blv_FSE	RF01785	1	1	1	1	1	0	0	0
FourU	RF01795	1	1	1	1	1	1	0.94	1
fstAT	RF01797	1	1	1	1	0.94	1	0.94	0.73
HSUR	RF01802	1	0.5	1	0.5	1	0.5	1	0.5
Lambda_thermo	RF01804	1	1	1	1	1	1	1	1
GIR1	RF01807	1	1	0.89	0.92	0.89	0.92	0.89	0.92
MicX	RF01808	1	1	1	1	1	1	1	1
symR	RF01809	1	1	1	1	1	1	1	1
PtaRNA1	RF01811	1	1	1	1	1	1	1	0.75
rdlD	RF01813	1	1	1	1	1	1	1	0.98
ROSE_2	RF01832	1	1	1	1	0.99	1	0.94	1
HIV_FS2	RF01835	1	1	1	1	1	1	1	0.79
ovine_lenti_FSE	RF01840	1	1	1	1	1	1	1	0.93
veev_FSE	RF01841	1	1	1	1	0.5	0.9	0.5	0.7
alpha_tmRNA	RF01849	1	1	1	1	1	1	0.98	1
tRNA-Sec	RF01852	0.9	0.32	0.53	0.28	0.43	0.28	0.08	0.28
MIAT_exon1	RF01874	1	1	1	1	1	1	0.98	0.9
MIAT_exon5_2	RF01876	1	1	1	1	1	1	1	1
HSR-omega_2	RF01886	1	1	1	1	1	1	0.86	1
mir-2241	RF01899	1	1	1	0.5	1	0.5	1	0.5
mir-284	RF01901	1	1	1	1	1	1	1	1
HEARO	RF02033	1	0.27	1	0.16	1	0.05	1	0.01
STnc630	RF02052	1	1	1	1	1	1	1	1
STnc370	RF02064	1	1	1	1	1	0.8	1	0.8
STnc180	RF02079	1	0.8	1	0.5	1	0.3	1	0.3

Continued on next page

Table 2 – continued from previous page

Rfam name	Rfam id	A	B	C	D	E	F	G	H
OrzO-P	RF02083	1	1	1	1	1	1	1	0.43
Yar_1	RF02085	1	0.9	1	0.8	1	0.5	1	0.3
tfoR	RF02100	1	1	1	1	1	1	1	1
IS009	RF02111	1	1	1	1	0.97	1	0.74	0.73
FAM13A-AS1_1	RF02114	0.92	1	0.92	0.8	0.92	0.3	0.92	0.2
FAM13A-AS1_2	RF02115	1	0.8	1	0.6	1	0.3	1	0.3
MEG8_3	RF02147	1	1	1	1	0.92	0.6	0.42	0.4
PVT1_4	RF02167	1	1	1	0.9	1	0.5	1	0.4
HPnc0260	RF02194	1	0.39	1	0.26	1	0.23	1	0.19
WT1-AS_1	RF02203	1	1	1	1	1	1	0.9	0.8
sX5	RF02224	1	1	1	1	1	1	0.59	0.8
sX11	RF02230	1	1	1	1	1	1	1	1
Six3os1_3	RF02248	1	1	1	1	1	0.9	0.89	0.8
Hammerhead_II	RF02276	1	0.83	1	0.79	0.73	0.54	0	0
Hammerhead_HH10	RF02277	1	1	1	1	1	1	1	1
hsp17	RF02358	1	0.67	1	0.67	0.83	0.67	0.33	0.67
PyrG_leader	RF02371	1	1	1	0.7	0.8	0.2	0.3	0.2
PyrD_leader	RF02373	1	0.59	1	0.15	1	0.11	1	0.11
Ms_AS-8	RF02466	1	1	1	1	1	1	0.78	0.8
Gl_RNase_MRP	RF02472	1	1	1	1	1	1	1	1
Gl_U1	RF02491	1	1	1	1	1	1	1	1
Gl_U2	RF02492	1	1	1	1	1	1	1	1
Gl_U4	RF02493	1	1	1	1	1	1	1	1
Gl_U6	RF02494	1	1	1	1	1	1	1	1
ohsC_RNA	RF02495	1	1	1	1	1	0.97	1	0.97
mir-2494	RF02518	1	1	1	0.9	1	0.9	1	0.7
ToxI	RF02519	1	1	1	1	1	1	0.62	0.5
ROSE_3	RF02523	1	1	1	1	1	1	1	0.88
NRF2_IRES	RF02531	0.98	1	0.98	1	0.97	0.95	0.86	0.9
MNV_3UTR	RF02532	1	1	1	1	1	1	1	1
ODC_IRES	RF02535	1	1	1	1	1	1	0.97	0.85
mt-tmRNA	RF02544	1	1	1	0.91	1	0.82	0.67	0.36

E Negative control set

We used coding sequences, ancestral repeats, untranslated regions (UTRs) and random sequences to perform a negative control. According to the procedure for structured and diverse RNA families the sequences of the negative control set were used as a input sequence for `RNAlien`. Taxonomic start points for the construction were set as below using taxids from NCBI taxonomy [2]. The results were summarized for each subset individually.

E.1 Random sequences

A test with 300 different 100 nucleotides long random sequences was performed. 100 Sequences each were used in Escherichia coli, Homo sapiens and Sulfolobus solfataricus. The sequences were created with a inhouse *randseq* program, source code will be provided on request by Ivo L. Hofacker (ivo@tbi.univie.ac.at).

E.2 Ancestral repeats

All 62 entries tagged with ancestral repeat from the `Dfam` [3] database were used with Homo sapiens as starting point for RNAlien, if the repeat was present there. The exceptions are the following list of pairs, with the first element containing the family name and the second the taxonomic start point: (Charlie12_Rodent,Mus musculus), (DNA9TA1_DR,Danio rerio), (L2-1_DR,Danio rerio), (Jockey2,Drosophila melanogaster), (DIVER2_I,Drosophila melanogaster)

E.3 Coding sequences

50 Protein coding sequences were checked for Escherichia coli, Sulfolobus solfataricus and Homo sapiens. Escherichia coli sequences are the first 50 annotated CDS sequences from regulonDB 9.0 [4] (`http://regulondb.ccg.unam.mx/menu/download/datasets/files/Gene_sequence.txt`) . Sulfolobus solfataricus sequences are retrieved from the reference genbank [5] assembly for Sulfolobus solfataricus $GCF\_000007005.1\_ASM700v1$. Homo sapiens sequences are from Ensemble [6] (Release 84, GRCh38.p5), chromosome2.

E.4 UTR regions

50 3-prime and 5-prime untranslated regions from E.coli and Homo sapiens were checked. Escherichia coli sequences are from regulonDB version 9.0 [4] (`http://regulondb.ccg.unam.mx/menu/download/datasets/files/UTR_5_3_sequence.txt`), Homo sapiens sequences are from Ensemble [6] (Release 84, GRCh38.p5), chromosome2. For suolfolobus we could not find a UTR dataset.

Table 3: Table for negative control set construction results. Shown are selected result fields of RNAz, RNAcode and cmstat. Column names A to Y are placeholders for following names: Name(=A), alienFastaNumber(=B), meanPairwiseIdentity(=C), shannonEntropy(=D), gcContent(=E), meanSingleSequenceMFE(=F), consensusMFE(=G), energyContribution(=H), covarianceContribution(=I), combinationsPair(=J), meanZScore(=K), SCI(=L), svmDecisionValue(=M), svmRNAClassProbability(=N), prediction(=O), RNAcodelowestp-value(=P), RNAcodeclassification(=Q), statSequenceNumber(=R), statEffectiveSequences(=S), statConsensusLength(=T), statW(=U), statBasepairs(=V), statBifurcations(=W), relativeEntropyCM(=X), relativeEntropyHMM(=Y)

A	B	C	D	E	F	G	H	I	J	K	L	M	N	O	P	Q	R	S	T	U	V	W	X	Y
hs_random1	1	-	-	-	-	-	-	-	-	-	-	-	-	-	-	-	1	0.7	100	118	28	1	0.591	0.318
hs_random2	1	-	-	-	-	-	-	-	-	-	-	-	-	-	-	-	1	0.61	100	118	34	1	0.59	0.266
hs_random3	1	-	-	-	-	-	-	-	-	-	-	-	-	-	-	-	1	0.75	100	118	24	2	0.589	0.369
hs_random4	1	-	-	-	-	-	-	-	-	-	-	-	-	-	-	-	1	0.78	100	118	26	3	0.59	0.34
hs_random5	1	-	-	-	-	-	-	-	-	-	-	-	-	-	-	-	1	0.75	100	118	27	2	0.591	0.335
hs_random6	1	-	-	-	-	-	-	-	-	-	-	-	-	-	-	-	1	0.75	100	118	27	4	0.592	0.335
hs_random7	1	-	-	-	-	-	-	-	-	-	-	-	-	-	-	-	1	0.69	100	117	27	2	0.589	0.335
hs_random8	1	-	-	-	-	-	-	-	-	-	-	-	-	-	-	-	1	0.69	100	119	28	2	0.59	0.319
hs_random9	1	-	-	-	-	-	-	-	-	-	-	-	-	-	-	-	1	0.71	100	118	29	0	0.589	0.322
hs_random10	1	-	-	-	-	-	-	-	-	-	-	-	-	-	-	-	1	0.72	100	132	26	2	0.59	0.348
hs_random11	1	-	-	-	-	-	-	-	-	-	-	-	-	-	-	-	1	0.65	100	117	33	1	0.589	0.276
hs_random12	1	-	-	-	-	-	-	-	-	-	-	-	-	-	-	-	1	0.78	100	118	28	1	0.59	0.321
hs_random13	1	-	-	-	-	-	-	-	-	-	-	-	-	-	-	-	1	0.8	100	118	20	1	0.589	0.401
hs_random14	1	-	-	-	-	-	-	-	-	-	-	-	-	-	-	-	1	0.62	100	118	33	2	0.589	0.278
hs_random15	1	-	-	-	-	-	-	-	-	-	-	-	-	-	-	-	1	0.85	100	117	19	2	0.591	0.414
hs_random16	1	-	-	-	-	-	-	-	-	-	-	-	-	-	-	-	1	0.79	100	117	23	0	0.588	0.371
hs_random17	1	-	-	-	-	-	-	-	-	-	-	-	-	-	-	-	1	0.75	100	118	27	1	0.59	0.336
hs_random18	1	-	-	-	-	-	-	-	-	-	-	-	-	-	-	-	1	0.72	100	118	26	1	0.589	0.343
hs_random19	1	-	-	-	-	-	-	-	-	-	-	-	-	-	-	-	1	0.79	100	118	26	2	0.591	0.34
hs_random20	1	-	-	-	-	-	-	-	-	-	-	-	-	-	-	-	1	0.76	100	118	29	2	0.591	0.328
hs_random21	1	-	-	-	-	-	-	-	-	-	-	-	-	-	-	-	1	0.65	100	118	33	2	0.59	0.282
hs_random22	1	-	-	-	-	-	-	-	-	-	-	-	-	-	-	-	1	0.69	100	117	31	2	0.589	0.299
hs_random23	1	-	-	-	-	-	-	-	-	-	-	-	-	-	-	-	1	0.69	100	118	27	1	0.59	0.331
hs_random24	1	-	-	-	-	-	-	-	-	-	-	-	-	-	-	-	1	0.69	100	118	31	1	0.591	0.291
hs_random25	1	-	-	-	-	-	-	-	-	-	-	-	-	-	-	-	1	0.69	100	118	30	3	0.589	0.303
hs_random26	1	-	-	-	-	-	-	-	-	-	-	-	-	-	-	-	1	0.71	100	118	31	1	0.59	0.287
hs_random27	1	-	-	-	-	-	-	-	-	-	-	-	-	-	-	-	1	0.66	100	117	30	1	0.59	0.306
hs_random28	1	-	-	-	-	-	-	-	-	-	-	-	-	-	-	-	1	0.73	100	119	28	2	0.589	0.326
hs_random29	1	-	-	-	-	-	-	-	-	-	-	-	-	-	-	-	1	0.69	100	117	30	1	0.591	0.308
hs_random30	1	-	-	-	-	-	-	-	-	-	-	-	-	-	-	-	1	0.62	100	117	35	1	0.59	0.249
hs_random31	1	-	-	-	-	-	-	-	-	-	-	-	-	-	-	-	1	0.76	100	118	27	1	0.589	0.334
hs_random32	1	-	-	-	-	-	-	-	-	-	-	-	-	-	-	-	1	0.94	100	118	18	1	0.592	0.432
hs_random33	1	-	-	-	-	-	-	-	-	-	-	-	-	-	-	-	1	0.87	100	118	22	0	0.59	0.39
hs_random34	1	-	-	-	-	-	-	-	-	-	-	-	-	-	-	-	1	0.69	100	119	32	1	0.59	0.288
hs_random35	1	-	-	-	-	-	-	-	-	-	-	-	-	-	-	-	1	0.68	100	118	28	2	0.59	0.318
hs_random36	1	-	-	-	-	-	-	-	-	-	-	-	-	-	-	-	1	0.73	100	118	26	2	0.591	0.341
hs_random37	1	-	-	-	-	-	-	-	-	-	-	-	-	-	-	-	1	0.75	100	118	25	2	0.589	0.35
hs_random38	1	-	-	-	-	-	-	-	-	-	-	-	-	-	-	-	1	0.67	100	119	32	2	0.59	0.281
hs_random39	1	-	-	-	-	-	-	-	-	-	-	-	-	-	-	-	1	0.77	100	118	24	2	0.59	0.365
hs_random40	1	-	-	-	-	-	-	-	-	-	-	-	-	-	-	-	1	0.82	100	117	24	1	0.591	0.369
hs_random41	1	-	-	-	-	-	-	-	-	-	-	-	-	-	-	-	1	0.75	100	118	26	2	0.589	0.345
hs_random42	1	-	-	-	-	-	-	-	-	-	-	-	-	-	-	-	1	0.82	100	118	25	1	0.591	0.356
hs_random43	1	-	-	-	-	-	-	-	-	-	-	-	-	-	-	-	1	0.76	100	117	25	2	0.59	0.352
hs_random44	1	-	-	-	-	-	-	-	-	-	-	-	-	-	-	-	1	0.73	100	118	27	2	0.59	0.33
hs_random45	1	-	-	-	-	-	-	-	-	-	-	-	-	-	-	-	1	0.65	100	118	30	1	0.59	0.305
hs_random46	1	-	-	-	-	-	-	-	-	-	-	-	-	-	-	-	1	0.78	100	118	23	2	0.591	0.371
hs_random47	1	-	-	-	-	-	-	-	-	-	-	-	-	-	-	-	1	0.82	100	118	22	1	0.588	0.376
hs_random48	1	-	-	-	-	-	-	-	-	-	-	-	-	-	-	-	1	0.59	100	118	34	1	0.59	0.259
hs_random49	1	-	-	-	-	-	-	-	-	-	-	-	-	-	-	-	1	0.65	100	118	30	1	0.589	0.299
hs_random50	1	-	-	-	-	-	-	-	-	-	-	-	-	-	-	-	1	0.76	100	117	26	1	0.59	0.347
hs_random51	1	-	-	-	-	-	-	-	-	-	-	-	-	-	-	-	1	0.71	100	118	29	0	0.59	0.313
hs_random52	1	-	-	-	-	-	-	-	-	-	-	-	-	-	-	-	1	0.69	100	118	31	2	0.589	0.289
hs_random53	1	-	-	-	-	-	-	-	-	-	-	-	-	-	-	-	1	0.73	100	118	26	1	0.589	0.351
hs_random54	1	-	-	-	-	-	-	-	-	-	-	-	-	-	-	-	1	0.71	100	118	28	2	0.589	0.328
hs_random55	1	-	-	-	-	-	-	-	-	-	-	-	-	-	-	-	1	0.64	100	118	31	1	0.591	0.289
hs_random56	1	-	-	-	-	-	-	-	-	-	-	-	-	-	-	-	1	0.77	100	118	25	0	0.591	0.354
hs_random57	1	-	-	-	-	-	-	-	-	-	-	-	-	-	-	-	1	0.71	100	117	31	1	0.591	0.298
hs_random58	1	-	-	-	-	-	-	-	-	-	-	-	-	-	-	-	1	0.74	100	118	25	1	0.592	0.358
hs_random59	1	-	-	-	-	-	-	-	-	-	-	-	-	-	-	-	1	0.78	100	118	26	3	0.591	0.346
hs_random60	1	-	-	-	-	-	-	-	-	-	-	-	-	-	-	-	1	0.67	100	118	29	1	0.59	0.319
hs_random61	1	-	-	-	-	-	-	-	-	-	-	-	-	-	-	-	1	0.67	100	118	31	0	0.591	0.287
hs_random62	1	-	-	-	-	-	-	-	-	-	-	-	-	-	-	-	1	0.72	100	118	28	0	0.59	0.322
hs_random63	1	-	-	-	-	-	-	-	-	-	-	-	-	-	-	-	1	0.75	100	119	26	1	0.59	0.342
hs_random64	1	-	-	-	-	-	-	-	-	-	-	-	-	-	-	-	1	0.83	100	118	25	1	0.591	0.356
hs_random65	1	-	-	-	-	-	-	-	-	-	-	-	-	-	-	-	1	0.91	100	117	21	0	0.589	0.395
hs_random66	1	-	-	-	-	-	-	-	-	-	-	-	-	-	-	-	1	0.8	100	118	25	1	0.589	0.363
hs_random67	1	-	-	-	-	-	-	-	-	-	-	-	-	-	-	-	1	0.73	100	118	29	2	0.59	0.317
hs_random68	1	-	-	-	-	-	-	-	-	-	-	-	-	-	-	-	1	0.73	100	118	30	2	0.589	0.31
hs_random69	1	-	-	-	-	-	-	-	-	-	-	-	-	-	-	-	1	0.68	100	118	32	1	0.589	0.278
hs_random70	1	-	-	-	-	-	-	-	-	-	-	-	-	-	-	-	1	0.74	100	118	28	1	0.59	0.322
hs_random71	1	-	-	-	-	-	-	-	-	-	-	-	-	-	-	-	1	0.69	100	118	30	1	0.59	0.31
hs_random72	1	-	-	-	-	-	-	-	-	-	-	-	-	-	-	-	1	0.75	100	118	26	1	0.591	0.344
hs_random73	1	-	-	-	-	-	-	-	-	-	-	-	-	-	-	-	1	0.72	100	118	29	3	0.588	0.312
hs_random74	1	-	-	-	-	-	-	-	-	-	-	-	-	-	-	-	1	0.91	100	118	18	2	0.591	0.424
hs_random75	1	-	-	-	-	-	-	-	-	-	-	-	-	-	-	-	1	0.79	100	118	29	1	0.59	0.314
hs_random76	1	-	-	-	-	-	-	-	-	-	-	-	-	-	-	-	1	0.72	100	119	26	1	0.591	0.345
hs_random77	1	-	-	-	-	-	-	-	-	-	-	-	-	-	-	-	1	0.69	100	117	29	2	0.59	0.319
hs_random78	1	-	-	-	-	-	-	-	-	-	-	-	-	-	-	-	1	0.79	100	118	26	1	0.59	0.353
hs_random79	1	-	-	-	-	-	-	-	-	-	-	-	-	-	-	-	1	0.84	100	118	24	1	0.589	0.365

Continued on next page

Table 3 – continued from previous page

A	B	C	D	E	F	G	H	I	J	K	L	M	N	O	P	Q	R	S	T	U	V	W	X	Y
hs_random80	1	-	-	-	-	-	-	-	-	-	-	-	-	-	-	-	1	0.83	100	118	23	2	0.589	0.379
hs_random81	1	-	-	-	-	-	-	-	-	-	-	-	-	-	-	-	1	0.82	100	118	24	0	0.591	0.363
hs_random82	1	-	-	-	-	-	-	-	-	-	-	-	-	-	-	-	1	0.79	100	118	23	2	0.589	0.364
hs_random83	1	-	-	-	-	-	-	-	-	-	-	-	-	-	-	-	1	0.65	100	117	32	1	0.591	0.284
hs_random84	1	-	-	-	-	-	-	-	-	-	-	-	-	-	-	-	1	0.72	100	118	27	2	0.59	0.337
hs_random85	1	-	-	-	-	-	-	-	-	-	-	-	-	-	-	-	1	0.7	100	118	31	1	0.591	0.304
hs_random86	1	-	-	-	-	-	-	-	-	-	-	-	-	-	-	-	1	0.71	100	118	30	2	0.59	0.308
hs_random87	1	-	-	-	-	-	-	-	-	-	-	-	-	-	-	-	1	0.78	100	118	24	1	0.588	0.372
hs_random88	1	-	-	-	-	-	-	-	-	-	-	-	-	-	-	-	1	0.73	100	118	28	2	0.59	0.326
hs_random89	1	-	-	-	-	-	-	-	-	-	-	-	-	-	-	-	1	0.89	100	118	21	2	0.589	0.398
hs_random90	1	-	-	-	-	-	-	-	-	-	-	-	-	-	-	-	1	0.77	100	118	26	1	0.59	0.342
hs_random91	1	-	-	-	-	-	-	-	-	-	-	-	-	-	-	-	1	0.74	100	132	25	2	0.59	0.348
hs_random92	1	-	-	-	-	-	-	-	-	-	-	-	-	-	-	-	1	0.79	100	118	27	1	0.589	0.339
hs_random93	1	-	-	-	-	-	-	-	-	-	-	-	-	-	-	-	1	0.82	100	118	23	1	0.589	0.378
hs_random94	1	-	-	-	-	-	-	-	-	-	-	-	-	-	-	-	1	0.75	100	118	25	0	0.589	0.348
hs_random95	1	-	-	-	-	-	-	-	-	-	-	-	-	-	-	-	1	0.7	100	118	31	1	0.59	0.298
hs_random96	1	-	-	-	-	-	-	-	-	-	-	-	-	-	-	-	1	0.75	100	118	24	2	0.59	0.362
hs_random97	1	-	-	-	-	-	-	-	-	-	-	-	-	-	-	-	1	0.66	100	117	33	2	0.591	0.274
hs_random98	1	-	-	-	-	-	-	-	-	-	-	-	-	-	-	-	1	0.7	100	118	28	1	0.59	0.324
hs_random99	1	-	-	-	-	-	-	-	-	-	-	-	-	-	-	-	1	0.71	100	132	28	2	0.591	0.332
hs_random100	1	-	-	-	-	-	-	-	-	-	-	-	-	-	-	-	1	0.71	100	119	29	2	0.591	0.316
ec_random101	1	-	-	-	-	-	-	-	-	-	-	-	-	-	-	-	1	0.67	100	118	30	1	0.592	0.306
ec_random102	1	-	-	-	-	-	-	-	-	-	-	-	-	-	-	-	1	0.75	100	118	23	3	0.589	0.368
ec_random103	1	-	-	-	-	-	-	-	-	-	-	-	-	-	-	-	1	0.73	100	118	27	1	0.589	0.333
ec_random104	1	-	-	-	-	-	-	-	-	-	-	-	-	-	-	-	1	0.75	100	118	27	2	0.589	0.335
ec_random105	1	-	-	-	-	-	-	-	-	-	-	-	-	-	-	-	1	0.71	100	118	30	3	0.59	0.307
ec_random106	1	-	-	-	-	-	-	-	-	-	-	-	-	-	-	-	1	0.71	100	118	27	1	0.589	0.329
ec_random107	1	-	-	-	-	-	-	-	-	-	-	-	-	-	-	-	1	0.82	100	118	21	2	0.592	0.4
ec_random108	1	-	-	-	-	-	-	-	-	-	-	-	-	-	-	-	1	0.73	100	119	27	3	0.589	0.331
ec_random109	1	-	-	-	-	-	-	-	-	-	-	-	-	-	-	-	1	0.69	100	118	27	2	0.591	0.336
ec_random110	1	-	-	-	-	-	-	-	-	-	-	-	-	-	-	-	1	0.78	100	117	27	0	0.591	0.339
ec_random111	1	-	-	-	-	-	-	-	-	-	-	-	-	-	-	-	1	0.72	100	117	29	0	0.591	0.329
ec_random112	1	-	-	-	-	-	-	-	-	-	-	-	-	-	-	-	1	0.7	100	118	29	0	0.589	0.313
ec_random113	1	-	-	-	-	-	-	-	-	-	-	-	-	-	-	-	1	0.69	100	118	32	0	0.591	0.29
ec_random114	1	-	-	-	-	-	-	-	-	-	-	-	-	-	-	-	1	0.82	100	118	25	2	0.591	0.369
ec_random115	1	-	-	-	-	-	-	-	-	-	-	-	-	-	-	-	1	0.72	100	118	31	0	0.59	0.303
ec_random116	1	-	-	-	-	-	-	-	-	-	-	-	-	-	-	-	1	0.82	100	118	24	2	0.591	0.366
ec_random117	1	-	-	-	-	-	-	-	-	-	-	-	-	-	-	-	1	0.68	100	119	28	1	0.59	0.319
ec_random118	1	-	-	-	-	-	-	-	-	-	-	-	-	-	-	-	1	0.79	100	118	27	0	0.589	0.334
ec_random119	1	-	-	-	-	-	-	-	-	-	-	-	-	-	-	-	1	0.79	100	118	26	2	0.59	0.344
ec_random120	1	-	-	-	-	-	-	-	-	-	-	-	-	-	-	-	1	0.82	100	118	23	1	0.591	0.374
ec_random121	1	-	-	-	-	-	-	-	-	-	-	-	-	-	-	-	1	0.66	100	119	30	1	0.59	0.306
ec_random122	1	-	-	-	-	-	-	-	-	-	-	-	-	-	-	-	1	0.71	100	118	28	1	0.591	0.321
ec_random123	1	-	-	-	-	-	-	-	-	-	-	-	-	-	-	-	1	0.73	100	118	29	0	0.59	0.316
ec_random124	1	-	-	-	-	-	-	-	-	-	-	-	-	-	-	-	1	0.65	100	119	29	2	0.589	0.31
ec_random125	1	-	-	-	-	-	-	-	-	-	-	-	-	-	-	-	1	0.64	100	118	31	0	0.59	0.305
ec_random126	1	-	-	-	-	-	-	-	-	-	-	-	-	-	-	-	1	0.75	100	118	29	2	0.589	0.319
ec_random127	1	-	-	-	-	-	-	-	-	-	-	-	-	-	-	-	1	0.74	100	132	28	3	0.591	0.327
ec_random128	1	-	-	-	-	-	-	-	-	-	-	-	-	-	-	-	1	0.79	100	117	26	0	0.591	0.346
ec_random129	1	-	-	-	-	-	-	-	-	-	-	-	-	-	-	-	1	0.79	100	117	28	1	0.589	0.328
ec_random130	1	-	-	-	-	-	-	-	-	-	-	-	-	-	-	-	1	0.71	100	118	27	1	0.592	0.333
ec_random131	1	-	-	-	-	-	-	-	-	-	-	-	-	-	-	-	1	0.74	100	118	27	2	0.589	0.331
ec_random132	1	-	-	-	-	-	-	-	-	-	-	-	-	-	-	-	1	0.77	100	118	24	2	0.59	0.372
ec_random133	1	-	-	-	-	-	-	-	-	-	-	-	-	-	-	-	1	0.68	100	117	34	0	0.591	0.273
ec_random134	1	-	-	-	-	-	-	-	-	-	-	-	-	-	-	-	1	0.78	100	118	29	2	0.589	0.323
ec_random135	1	-	-	-	-	-	-	-	-	-	-	-	-	-	-	-	1	0.78	100	118	25	2	0.589	0.359
ec_random136	1	-	-	-	-	-	-	-	-	-	-	-	-	-	-	-	1	0.82	100	117	24	0	0.59	0.363
ec_random137	1	-	-	-	-	-	-	-	-	-	-	-	-	-	-	-	1	0.75	100	118	25	2	0.589	0.348
ec_random138	1	-	-	-	-	-	-	-	-	-	-	-	-	-	-	-	1	0.75	100	118	27	1	0.591	0.34
ec_random139	1	-	-	-	-	-	-	-	-	-	-	-	-	-	-	-	1	0.82	100	118	25	0	0.591	0.358
ec_random140	1	-	-	-	-	-	-	-	-	-	-	-	-	-	-	-	1	0.69	100	118	30	2	0.591	0.309
ec_random141	1	-	-	-	-	-	-	-	-	-	-	-	-	-	-	-	1	0.75	100	118	25	1	0.588	0.355
ec_random142	1	-	-	-	-	-	-	-	-	-	-	-	-	-	-	-	1	0.7	100	118	28	1	0.59	0.322
ec_random143	1	-	-	-	-	-	-	-	-	-	-	-	-	-	-	-	1	0.73	100	118	27	1	0.589	0.345
ec_random144	1	-	-	-	-	-	-	-	-	-	-	-	-	-	-	-	1	0.81	100	118	25	1	0.588	0.348
ec_random145	1	-	-	-	-	-	-	-	-	-	-	-	-	-	-	-	1	0.77	100	118	25	1	0.59	0.355
ec_random146	1	-	-	-	-	-	-	-	-	-	-	-	-	-	-	-	1	0.77	100	118	24	3	0.591	0.36
ec_random147	1	-	-	-	-	-	-	-	-	-	-	-	-	-	-	-	1	0.98	100	118	18	1	0.591	0.429
ec_random148	1	-	-	-	-	-	-	-	-	-	-	-	-	-	-	-	1	0.89	100	118	18	1	0.591	0.418
ec_random149	1	-	-	-	-	-	-	-	-	-	-	-	-	-	-	-	1	0.64	100	118	32	2	0.591	0.289
ec_random150	1	-	-	-	-	-	-	-	-	-	-	-	-	-	-	-	1	0.73	100	118	29	1	0.591	0.318
ec_random151	1	-	-	-	-	-	-	-	-	-	-	-	-	-	-	-	1	0.79	100	118	30	1	0.589	0.313
ec_random152	1	-	-	-	-	-	-	-	-	-	-	-	-	-	-	-	1	0.71	100	119	29	2	0.589	0.312
ec_random153	1	-	-	-	-	-	-	-	-	-	-	-	-	-	-	-	1	0.79	100	118	24	2	0.589	0.365
ec_random154	1	-	-	-	-	-	-	-	-	-	-	-	-	-	-	-	1	0.65	100	118	32	3	0.591	0.288
ec_random155	1	-	-	-	-	-	-	-	-	-	-	-	-	-	-	-	1	0.75	100	118	28	2	0.59	0.328
ec_random156	1	-	-	-	-	-	-	-	-	-	-	-	-	-	-	-	1	0.79	100	118	26	1	0.59	0.342
ec_random157	1	-	-	-	-	-	-	-	-	-	-	-	-	-	-	-	1	0.71	100	118	28	1	0.59	0.331
ec_random158	1	-	-	-	-	-	-	-	-	-	-	-	-	-	-	-	1	0.67	100	118	34	3	0.591	0.26
ec_random159	1	-	-	-	-	-	-	-	-	-	-	-	-	-	-	-	1	0.7	100	118	28	2	0.591	0.323
ec_random160	1	-	-	-	-	-	-	-	-	-	-	-	-	-	-	-	1	0.78	100	118	24	3	0.589	0.368
ec_random161	1	-	-	-	-	-	-	-	-	-	-	-	-	-	-	-	1	0.63	100	116	35	0	0.589	0.251
ec_random162	1	-	-	-	-	-	-	-	-	-	-	-	-	-	-	-	1	0.69	100	118	28	1	0.591	0.328
ec_random163	1	-	-	-	-	-	-	-	-	-	-	-	-	-	-	-	1	0.81	100	118	26	0	0.59	0.352
ec_random164	1	-	-	-	-	-	-	-	-	-	-	-	-	-	-	-	1	0.76	100	131	25	2	0.589	0.353
ec_random165	1	-	-	-	-	-	-	-	-	-	-	-	-	-	-	-	1	0.83	100	118	23	1	0.591	0.374
ec_random166	1	-	-	-	-	-	-	-	-	-	-	-	-	-	-	-	1	0.68	100	118	32	3	0.59	0.293
ec_random167	1	-	-	-	-	-	-	-	-	-	-	-	-	-	-	-	1	0.82	100	118	22	0	0.589	0.381

Continued on next page

Table 3 – continued from previous page

A	B	C	D	E	F	G	H	I	J	K	L	M	N	O	P	Q	R	S	T	U	V	W	X	Y
ec_random168	1	-	-	-	-	-	-	-	-	-	-	-	-	-	-	-	1	0.74	100	118	29	2	0.592	0.319
ec_random169	1	-	-	-	-	-	-	-	-	-	-	-	-	-	-	-	1	0.79	100	118	23	3	0.59	0.377
ec_random170	1	-	-	-	-	-	-	-	-	-	-	-	-	-	-	-	1	0.71	100	118	30	1	0.59	0.301
ec_random171	1	-	-	-	-	-	-	-	-	-	-	-	-	-	-	-	1	0.71	100	118	30	1	0.589	0.3
ec_random172	1	-	-	-	-	-	-	-	-	-	-	-	-	-	-	-	1	0.72	100	116	27	0	0.59	0.331
ec_random173	1	-	-	-	-	-	-	-	-	-	-	-	-	-	-	-	1	0.67	100	118	32	0	0.591	0.294
ec_random174	1	-	-	-	-	-	-	-	-	-	-	-	-	-	-	-	1	0.7	100	118	29	2	0.591	0.316
ec_random175	1	-	-	-	-	-	-	-	-	-	-	-	-	-	-	-	1	0.68	100	118	30	1	0.589	0.307
ec_random176	1	-	-	-	-	-	-	-	-	-	-	-	-	-	-	-	1	0.72	100	118	27	1	0.589	0.333
ec_random177	1	-	-	-	-	-	-	-	-	-	-	-	-	-	-	-	1	0.71	100	118	29	1	0.591	0.317
ec_random178	1	-	-	-	-	-	-	-	-	-	-	-	-	-	-	-	1	0.76	100	119	26	1	0.589	0.34
ec_random179	1	-	-	-	-	-	-	-	-	-	-	-	-	-	-	-	1	0.75	100	119	26	1	0.591	0.345
ec_random180	1	-	-	-	-	-	-	-	-	-	-	-	-	-	-	-	1	0.78	100	118	25	0	0.589	0.356
ec_random181	1	-	-	-	-	-	-	-	-	-	-	-	-	-	-	-	1	0.75	100	118	29	1	0.589	0.325
ec_random182	1	-	-	-	-	-	-	-	-	-	-	-	-	-	-	-	1	0.79	100	117	26	0	0.589	0.349
ec_random183	1	-	-	-	-	-	-	-	-	-	-	-	-	-	-	-	1	0.74	100	118	23	2	0.589	0.369
ec_random184	1	-	-	-	-	-	-	-	-	-	-	-	-	-	-	-	1	0.89	100	117	24	1	0.589	0.375
ec_random185	1	-	-	-	-	-	-	-	-	-	-	-	-	-	-	-	1	0.72	100	118	27	4	0.591	0.335
ec_random186	1	-	-	-	-	-	-	-	-	-	-	-	-	-	-	-	1	0.8	100	119	21	2	0.59	0.388
ec_random187	1	-	-	-	-	-	-	-	-	-	-	-	-	-	-	-	1	0.61	100	118	33	1	0.589	0.274
ec_random188	1	-	-	-	-	-	-	-	-	-	-	-	-	-	-	-	1	0.61	100	118	33	1	0.589	0.269
ec_random189	1	-	-	-	-	-	-	-	-	-	-	-	-	-	-	-	1	0.68	100	118	32	3	0.589	0.278
ec_random190	1	-	-	-	-	-	-	-	-	-	-	-	-	-	-	-	1	0.74	100	118	28	1	0.59	0.329
ec_random191	1	-	-	-	-	-	-	-	-	-	-	-	-	-	-	-	1	0.77	100	118	24	2	0.59	0.369
ec_random192	1	-	-	-	-	-	-	-	-	-	-	-	-	-	-	-	1	0.75	100	118	29	1	0.591	0.321
ec_random193	1	-	-	-	-	-	-	-	-	-	-	-	-	-	-	-	1	0.8	100	118	25	2	0.59	0.351
ec_random194	1	-	-	-	-	-	-	-	-	-	-	-	-	-	-	-	1	0.85	100	118	22	1	0.59	0.388
ec_random195	1	-	-	-	-	-	-	-	-	-	-	-	-	-	-	-	1	0.77	100	118	26	2	0.589	0.349
ec_random196	1	-	-	-	-	-	-	-	-	-	-	-	-	-	-	-	1	0.66	100	117	30	0	0.59	0.297
ec_random197	1	-	-	-	-	-	-	-	-	-	-	-	-	-	-	-	1	0.71	100	117	28	1	0.59	0.332
ec_random198	1	-	-	-	-	-	-	-	-	-	-	-	-	-	-	-	1	0.71	100	132	27	2	0.591	0.345
ec_random199	1	-	-	-	-	-	-	-	-	-	-	-	-	-	-	-	1	0.87	100	118	23	1	0.592	0.388
ec_random200	1	-	-	-	-	-	-	-	-	-	-	-	-	-	-	-	1	0.7	100	119	27	3	0.589	0.334
ss_random201	1	-	-	-	-	-	-	-	-	-	-	-	-	-	-	-	1	0.77	100	118	32	0	0.591	0.294
ss_random202	1	-	-	-	-	-	-	-	-	-	-	-	-	-	-	-	1	0.72	100	118	30	0	0.591	0.302
ss_random203	1	-	-	-	-	-	-	-	-	-	-	-	-	-	-	-	1	0.73	100	131	28	2	0.59	0.324
ss_random204	1	-	-	-	-	-	-	-	-	-	-	-	-	-	-	-	1	0.62	100	132	33	2	0.591	0.279
ss_random205	1	-	-	-	-	-	-	-	-	-	-	-	-	-	-	-	1	0.66	100	119	32	1	0.591	0.282
ss_random206	1	-	-	-	-	-	-	-	-	-	-	-	-	-	-	-	1	0.71	100	119	27	2	0.589	0.329
ss_random207	1	-	-	-	-	-	-	-	-	-	-	-	-	-	-	-	1	0.72	100	119	28	1	0.591	0.326
ss_random208	1	-	-	-	-	-	-	-	-	-	-	-	-	-	-	-	1	0.73	100	118	27	1	0.59	0.336
ss_random209	1	-	-	-	-	-	-	-	-	-	-	-	-	-	-	-	1	0.73	100	118	27	3	0.59	0.334
ss_random210	1	-	-	-	-	-	-	-	-	-	-	-	-	-	-	-	1	0.71	100	118	30	2	0.591	0.313
ss_random211	1	-	-	-	-	-	-	-	-	-	-	-	-	-	-	-	1	0.8	100	118	25	1	0.589	0.358
ss_random212	1	-	-	-	-	-	-	-	-	-	-	-	-	-	-	-	1	0.73	100	118	27	4	0.591	0.328
ss_random213	1	-	-	-	-	-	-	-	-	-	-	-	-	-	-	-	1	0.77	100	118	25	2	0.59	0.355
ss_random214	1	-	-	-	-	-	-	-	-	-	-	-	-	-	-	-	1	0.73	100	119	27	3	0.589	0.343
ss_random215	1	-	-	-	-	-	-	-	-	-	-	-	-	-	-	-	1	0.85	100	117	23	1	0.588	0.374
ss_random216	1	-	-	-	-	-	-	-	-	-	-	-	-	-	-	-	1	0.83	100	118	25	1	0.59	0.356
ss_random217	1	-	-	-	-	-	-	-	-	-	-	-	-	-	-	-	1	0.79	100	118	24	2	0.588	0.362
ss_random218	1	-	-	-	-	-	-	-	-	-	-	-	-	-	-	-	1	0.75	100	118	27	2	0.591	0.335
ss_random219	1	-	-	-	-	-	-	-	-	-	-	-	-	-	-	-	1	0.81	100	118	21	1	0.591	0.397
ss_random220	1	-	-	-	-	-	-	-	-	-	-	-	-	-	-	-	1	0.72	100	118	27	1	0.59	0.336
ss_random221	1	-	-	-	-	-	-	-	-	-	-	-	-	-	-	-	1	0.68	100	118	29	1	0.591	0.314
ss_random222	1	-	-	-	-	-	-	-	-	-	-	-	-	-	-	-	1	0.79	100	117	23	1	0.59	0.369
ss_random223	1	-	-	-	-	-	-	-	-	-	-	-	-	-	-	-	1	0.65	100	117	34	1	0.591	0.268
ss_random224	1	-	-	-	-	-	-	-	-	-	-	-	-	-	-	-	1	0.86	100	118	24	1	0.592	0.376
ss_random225	1	-	-	-	-	-	-	-	-	-	-	-	-	-	-	-	1	0.79	100	118	24	1	0.59	0.362
ss_random226	1	-	-	-	-	-	-	-	-	-	-	-	-	-	-	-	1	0.75	100	118	27	1	0.59	0.34
ss_random227	1	-	-	-	-	-	-	-	-	-	-	-	-	-	-	-	1	0.82	100	118	21	1	0.59	0.389
ss_random228	1	-	-	-	-	-	-	-	-	-	-	-	-	-	-	-	1	0.79	100	117	26	0	0.591	0.341
ss_random229	1	-	-	-	-	-	-	-	-	-	-	-	-	-	-	-	1	0.64	100	118	32	1	0.589	0.279
ss_random230	1	-	-	-	-	-	-	-	-	-	-	-	-	-	-	-	1	0.74	100	118	27	2	0.59	0.344
ss_random231	1	-	-	-	-	-	-	-	-	-	-	-	-	-	-	-	1	0.71	100	118	26	2	0.588	0.342
ss_random232	1	-	-	-	-	-	-	-	-	-	-	-	-	-	-	-	1	0.79	100	118	26	1	0.591	0.339
ss_random233	1	-	-	-	-	-	-	-	-	-	-	-	-	-	-	-	1	0.71	100	118	33	0	0.59	0.293
ss_random234	1	-	-	-	-	-	-	-	-	-	-	-	-	-	-	-	1	0.8	100	118	25	2	0.589	0.355
ss_random235	1	-	-	-	-	-	-	-	-	-	-	-	-	-	-	-	1	0.65	100	117	29	1	0.591	0.319
ss_random236	1	-	-	-	-	-	-	-	-	-	-	-	-	-	-	-	1	0.6	100	118	34	1	0.59	0.261
ss_random237	1	-	-	-	-	-	-	-	-	-	-	-	-	-	-	-	1	0.87	100	118	18	1	0.588	0.421
ss_random238	1	-	-	-	-	-	-	-	-	-	-	-	-	-	-	-	1	0.67	100	117	31	2	0.59	0.291
ss_random239	1	-	-	-	-	-	-	-	-	-	-	-	-	-	-	-	1	0.64	100	118	32	2	0.589	0.28
ss_random240	1	-	-	-	-	-	-	-	-	-	-	-	-	-	-	-	1	0.61	100	118	32	2	0.591	0.284
ss_random241	1	-	-	-	-	-	-	-	-	-	-	-	-	-	-	-	1	0.85	100	118	22	0	0.591	0.381
ss_random242	1	-	-	-	-	-	-	-	-	-	-	-	-	-	-	-	1	0.78	100	118	25	2	0.59	0.359
ss_random243	1	-	-	-	-	-	-	-	-	-	-	-	-	-	-	-	1	0.68	100	118	31	1	0.59	0.293
ss_random244	1	-	-	-	-	-	-	-	-	-	-	-	-	-	-	-	1	0.67	100	118	29	1	0.59	0.319
ss_random245	1	-	-	-	-	-	-	-	-	-	-	-	-	-	-	-	1	0.76	100	118	26	2	0.589	0.354
ss_random246	1	-	-	-	-	-	-	-	-	-	-	-	-	-	-	-	1	0.74	100	118	29	1	0.59	0.314
ss_random247	1	-	-	-	-	-	-	-	-	-	-	-	-	-	-	-	1	0.66	100	117	33	1	0.59	0.27
ss_random248	1	-	-	-	-	-	-	-	-	-	-	-	-	-	-	-	1	0.86	100	118	18	0	0.59	0.423
ss_random249	1	-	-	-	-	-	-	-	-	-	-	-	-	-	-	-	1	0.61	100	117	31	0	0.589	0.29
ss_random250	1	-	-	-	-	-	-	-	-	-	-	-	-	-	-	-	1	0.75	100	118	28	1	0.589	0.318
ss_random251	1	-	-	-	-	-	-	-	-	-	-	-	-	-	-	-	1	0.69	100	117	30	2	0.59	0.306
ss_random252	1	-	-	-	-	-	-	-	-	-	-	-	-	-	-	-	1	0.75	100	118	24	1	0.59	0.363
ss_random253	1	-	-	-	-	-	-	-	-	-	-	-	-	-	-	-	1	0.68	100	118	25	2	0.59	0.352
ss_random254	1	-	-	-	-	-	-	-	-	-	-	-	-	-	-	-	1	0.75	100	118	26	2	0.591	0.343
ss_random255	1	-	-	-	-	-	-	-	-	-	-	-	-	-	-	-	1	0.88	100	118	17	1	0.589	0.435

Continued on next page

Table 3 – continued from previous page

A	B	C	D	E	F	G	H	I	J	K	L	M	N	O	P	Q	R	S	T	U	V	W	X	Y
ss_random256	1	-	-	-	-	-	-	-	-	-	-	-	-	-	-	-	1	0.78	100	117	29	2	0.59	0.324
ss_random257	1	-	-	-	-	-	-	-	-	-	-	-	-	-	-	-	1	0.76	100	118	24	1	0.59	0.366
ss_random258	1	-	-	-	-	-	-	-	-	-	-	-	-	-	-	-	1	0.75	100	119	25	2	0.592	0.355
ss_random259	1	-	-	-	-	-	-	-	-	-	-	-	-	-	-	-	1	0.68	100	118	30	1	0.589	0.306
ss_random260	1	-	-	-	-	-	-	-	-	-	-	-	-	-	-	-	1	0.71	100	118	32	2	0.591	0.291
ss_random261	1	-	-	-	-	-	-	-	-	-	-	-	-	-	-	-	1	0.77	100	119	25	1	0.589	0.351
ss_random262	1	-	-	-	-	-	-	-	-	-	-	-	-	-	-	-	1	0.67	100	118	30	1	0.591	0.307
ss_random263	1	-	-	-	-	-	-	-	-	-	-	-	-	-	-	-	1	0.8	100	118	27	1	0.591	0.349
ss_random264	1	-	-	-	-	-	-	-	-	-	-	-	-	-	-	-	1	0.8	100	119	23	2	0.591	0.372
ss_random265	1	-	-	-	-	-	-	-	-	-	-	-	-	-	-	-	1	0.62	100	118	32	0	0.591	0.28
ss_random266	1	-	-	-	-	-	-	-	-	-	-	-	-	-	-	-	1	0.7	100	118	30	2	0.59	0.308
ss_random267	1	-	-	-	-	-	-	-	-	-	-	-	-	-	-	-	1	0.71	100	118	27	1	0.589	0.331
ss_random268	1	-	-	-	-	-	-	-	-	-	-	-	-	-	-	-	1	0.68	100	118	29	1	0.589	0.314
ss_random269	1	-	-	-	-	-	-	-	-	-	-	-	-	-	-	-	1	0.73	100	118	29	1	0.59	0.323
ss_random270	1	-	-	-	-	-	-	-	-	-	-	-	-	-	-	-	1	0.71	100	119	24	3	0.591	0.36
ss_random271	1	-	-	-	-	-	-	-	-	-	-	-	-	-	-	-	1	0.79	100	118	23	2	0.59	0.372
ss_random272	1	-	-	-	-	-	-	-	-	-	-	-	-	-	-	-	1	0.62	100	118	32	2	0.591	0.283
ss_random273	1	-	-	-	-	-	-	-	-	-	-	-	-	-	-	-	1	0.69	100	119	28	2	0.591	0.323
ss_random274	1	-	-	-	-	-	-	-	-	-	-	-	-	-	-	-	1	0.71	100	118	26	0	0.591	0.345
ss_random275	1	-	-	-	-	-	-	-	-	-	-	-	-	-	-	-	1	0.82	100	118	22	2	0.59	0.381
ss_random276	1	-	-	-	-	-	-	-	-	-	-	-	-	-	-	-	1	0.74	100	118	28	2	0.591	0.327
ss_random277	1	-	-	-	-	-	-	-	-	-	-	-	-	-	-	-	1	0.76	100	118	26	1	0.592	0.354
ss_random278	1	-	-	-	-	-	-	-	-	-	-	-	-	-	-	-	1	0.67	100	118	29	1	0.59	0.316
ss_random279	1	-	-	-	-	-	-	-	-	-	-	-	-	-	-	-	1	0.67	100	118	31	2	0.591	0.295
ss_random280	1	-	-	-	-	-	-	-	-	-	-	-	-	-	-	-	1	0.68	100	118	31	1	0.589	0.29
ss_random281	1	-	-	-	-	-	-	-	-	-	-	-	-	-	-	-	1	0.65	100	118	28	2	0.589	0.321
ss_random282	1	-	-	-	-	-	-	-	-	-	-	-	-	-	-	-	1	0.78	100	118	25	1	0.591	0.351
ss_random283	1	-	-	-	-	-	-	-	-	-	-	-	-	-	-	-	1	0.68	100	118	31	1	0.591	0.286
ss_random284	1	-	-	-	-	-	-	-	-	-	-	-	-	-	-	-	1	0.78	100	118	25	1	0.59	0.357
ss_random285	1	-	-	-	-	-	-	-	-	-	-	-	-	-	-	-	1	0.7	100	118	29	2	0.592	0.317
ss_random286	1	-	-	-	-	-	-	-	-	-	-	-	-	-	-	-	1	0.71	100	117	33	1	0.591	0.277
ss_random287	1	-	-	-	-	-	-	-	-	-	-	-	-	-	-	-	1	0.75	100	118	28	1	0.591	0.322
ss_random288	1	-	-	-	-	-	-	-	-	-	-	-	-	-	-	-	1	0.73	100	118	29	1	0.591	0.315
ss_random289	1	-	-	-	-	-	-	-	-	-	-	-	-	-	-	-	1	0.75	100	118	25	1	0.589	0.354
ss_random290	1	-	-	-	-	-	-	-	-	-	-	-	-	-	-	-	1	0.74	100	119	28	2	0.59	0.328
ss_random291	1	-	-	-	-	-	-	-	-	-	-	-	-	-	-	-	1	0.66	100	118	31	2	0.59	0.289
ss_random292	1	-	-	-	-	-	-	-	-	-	-	-	-	-	-	-	1	0.79	100	118	25	2	0.589	0.353
ss_random293	1	-	-	-	-	-	-	-	-	-	-	-	-	-	-	-	1	0.86	100	118	21	2	0.589	0.391
ss_random294	1	-	-	-	-	-	-	-	-	-	-	-	-	-	-	-	1	0.71	100	118	28	1	0.588	0.316
ss_random295	1	-	-	-	-	-	-	-	-	-	-	-	-	-	-	-	1	0.66	100	118	33	1	0.59	0.275
ss_random296	1	-	-	-	-	-	-	-	-	-	-	-	-	-	-	-	1	0.76	100	118	23	1	0.588	0.37
ss_random297	1	-	-	-	-	-	-	-	-	-	-	-	-	-	-	-	1	0.76	100	118	25	2	0.59	0.356
ss_random298	1	-	-	-	-	-	-	-	-	-	-	-	-	-	-	-	1	0.74	100	131	25	1	0.589	0.347
ss_random299	1	-	-	-	-	-	-	-	-	-	-	-	-	-	-	-	1	0.77	100	118	27	3	0.591	0.333
ss_random300	1	-	-	-	-	-	-	-	-	-	-	-	-	-	-	-	1	0.68	100	118	29	2	0.591	0.311
ancestral301	4	70.78	0.38461	0.44033	-130.03	-74.85	-74.01	-0.84	1.24	0.11	0.58	-2.35	0.00001	OTHER	0.472	OTHER	4	0.94	429	567	125	6	0.59	0.325
ancestral302	36	83.43	0.32333	0.40133	-227.71	-159.55	-158.33	-1.22	1.26	-0.92	0.7	-0.61	0.159931	OTHER	0.00002425	PROTEIN	36	2.87	743	2198	137	17	0.59	0.466
ancestral303	1	-	-	-	-	-	-	-	-	-	-	-	-	-	-	-	1	0.72	1419	1444	363	28	0.59	0.343
ancestral304	24	79.36	0.38635	0.34247	-141.15	-79.86	-80.67	0.81	1.33	-0.86	0.57	-1.12	0.04612	OTHER	0.453	OTHER	24	3.44	560	798	122	8	0.59	0.445
ancestral305	21	66.08	0.63164	0.33584	-134.74	-21.02	-22.72	1.69	1.61	0.86	0.16	-4.4	0	OTHER	0.279	OTHER	21	3.86	613	716	99	10	0.59	0.489
ancestral306	48	74.65	0.4543	0.40367	-134.35	-64.28	-67.74	3.46	1.33	-0.46	0.48	-1.85	0.006953	OTHER	0.174	OTHER	48	3.8	504	689	89	9	0.59	0.477
ancestral307	26	66.88	0.56316	0.36765	-147.39	-54.05	-52.73	-1.32	1.5	-0.08	0.37	-2.47	0.000005	OTHER	0.88	OTHER	26	4.56	593	1112	74	6	0.59	0.514
ancestral308	27	55.36	0.72936	0.36114	-179.43	-25.97	-28.09	2.12	1.72	-0.02	0.14	-3.09	0	OTHER	0.193	OTHER	27	4.07	703	2471	113	12	0.59	0.487
ancestral309	28	81.68	0.33225	0.34702	-140.49	-71.93	-75.53	3.6	1.2	0.81	0.51	-3.87	0	OTHER	0.268	OTHER	28	4.64	619	968	86	9	0.59	0.505
ancestral310	0	-	-	-	-	-	-	-	-	-	-	-	-	-	-	-	-	-	-	-	-	-	-	-
ancestral311	3	97.1	0.03993	0.33497	-9.67	-8.9	-9.23	0.33	1	0.8	0.92	-2.97	0	OTHER	0.234	OTHER	3	1.46	69	86	17	1	0.814	0.611
ancestral312	66	76.09	0.44929	0.28298	-36.06	-7.49	-6.3	-1.19	1.44	0.75	0.21	-4.8	0	OTHER	0.198	OTHER	66	3.98	226	256	41	2	0.59	0.473
ancestral313	30	64.14	0.67586	0.35367	-126.18	-22.65	-25.78	3.13	1.55	0.65	0.18	-3.88	0	OTHER	0.825	OTHER	30	4.65	556	695	80	7	0.59	0.501
ancestral314	4	54.65	0.71539	0.35137	-159.78	-31.56	-24.5	-7.06	1.81	-0.17	0.2	-2.66	0.000002	OTHER	0.093	OTHER	4	1.81	687	882	157	13	0.589	0.409
ancestral315	38	61.23	0.71454	0.3754	-154.57	-30.28	-29.08	-1.2	1.57	-0.85	0.2	-1.84	0.007176	OTHER	0.000695	PROTEIN	38	4.65	654	1148	84	8	0.59	0.512
ancestral316	49	61.73	0.7012	0.39975	-138.36	-32.35	-35.58	3.23	1.62	-0.22	0.23	-2.49	0.000005	OTHER	0.068	OTHER	49	4.9	562	637	69	5	0.59	0.52
ancestral317	43	67.43	0.61204	0.35571	-107.73	-20.28	-18.5	-1.79	1.56	0.72	0.19	-4.18	0	OTHER	0.375	OTHER	43	5.1	480	545	77	3	0.59	0.496
ancestral318	42	57.09	0.79051	0.37492	-130.29	-24.33	-22.88	-1.45	1.73	0.45	0.19	-3.14	0	OTHER	0.889	OTHER	42	6.51	496	1324	60	7	0.59	0.519
ancestral319	44	69.88	0.55626	0.33964	-93.68	-25.06	-25.29	0.23	1.49	0.44	0.27	-3.68	0	OTHER	0.819	OTHER	44	4.68	427	519	80	7	0.59	0.475
ancestral320	22	60.07	0.73414	0.37267	-145.46	-25.14	-23.56	-1.58	1.73	0.28	0.17	-3.27	0	OTHER	0.294	OTHER	22	3.99	553	1045	102	7	0.59	0.472
ancestral321	31	64.12	0.63227	0.3663	-144.59	-33.73	-31.12	-2.61	1.7	0.4	0.23	-3.52	0	OTHER	0.249	OTHER	31	4.38	582	1327	93	6	0.59	0.49
ancestral322	35	72.84	0.48502	0.38149	-163.69	-62.83	-65.55	2.72	1.42	0.14	0.38	-3.04	0	OTHER	0.12	OTHER	35	4.32	653	980	97	7	0.59	0.495
ancestral323	32	76	0.45257	0.3777	-185.43	-75.16	-72.87	-2.3	1.53	0.11	0.41	-2.98	0	OTHER	0.622	OTHER	32	4.05	717	936	145	5	0.59	0.458
ancestral324	0	-	-	-	-	-	-	-	-	-	-	-	-	-	-	-	-	-	-	-	-	-	-	-
ancestral325	3	57.89	0.4211	0.36727	-310.1	-163.29	-162.79	-0.5	1.32	-3.32	0.53	2	0.994941	RNA	-	-	3	1.15	1097	1135	318	21	0.59	0.331
ancestral326	1	-	-	-	-	-	-	-	-	-	-	-	-	-	-	-	1	0.75	686	710	181	10	0.589	0.334
ancestral327	1	-	-	-	-	-	-	-	-	-	-	-	-	-	-	-	1	0.71	547	571	157	10	0.589	0.312
ancestral328	98	68.03	0.54084	0.35433	-86.32	-23.37	-21.17	-2.2	1.64	-0.43	0.27	-2.68	0.000002	OTHER	0.546	OTHER	98	4.83	363	528	53	4	0.59	0.501
ancestral329	22	59.5	0.71233	0.34629	-193.59	-22.79	-21.85	-0.95	1.66	0.03	0.12	-3.32	0	OTHER	0.042	PROTEIN	22	4.73	836	1513	100	8	0.59	0.518
ancestral330	19	57.62	0.78843	0.32483	-125.55	-9.05	-8.79	-0.26	1.82	-0.36	0.07	-2.83	0.000001	OTHER	0.115	OTHER	19	5.29	578	734	53	5	0.59	0.536
ancestral331	2	56.59	0.43409	0.3613	-102.01	-36.69	-33.44	-3.25	1.38	-1.05	0.36	-1.85	0.006859	OTHER	-	-	2	1.14	436	466	147	9	0.59	0.296
ancestral332	1	-	-	-	-	-	-	-	-	-	-	-	-	-	-	-	1	0.75	575	599	159	13	0.59	0.32
ancestral333	0	-	-	-	-	-	-	-	-	-	-	-	-	-	-	-	-	-	-	-	-	-	-	-
ancestral334	0	-	-	-	-	-	-	-	-	-	-	-	-	-	-	-	-	-	-	-	-	-	-	-
ancestral335	0	-	-	-	-	-	-	-	-	-	-	-	-	-	-	-	-	-	-	-	-	-	-	-
ancestral336	65	76.46	0.44218	0.43976	-76.53	-27.67	-27.49	-0.18	1.37	-0.96	0.36	-1.93	0.005543	OTHER	0.976	OTHER	65	2.1	282	351	61	6	0.59	0.426
ancestral337	0	-	-	-	-	-	-	-	-	-	-	-	-	-	-	-	-	-	-	-	-	-	-	-
ancestral338	0	-	-	-	-	-	-	-	-	-	-	-	-	-	-	-	-	-	-	-	-	-	-	-
ancestral339	0	-	-	-	-	-	-	-	-	-	-	-	-	-	-	-	-	-	-	-	-	-	-	-
ancestral340	0	-	-	-	-	-	-	-	-	-	-	-	-	-	-	-	-	-	-	-	-	-	-	-
ancestral341	0	-	-	-	-	-	-	-	-	-	-	-	-	-	-	-	-	-	-	-	-	-	-	-
ancestral342	0	-	-	-	-	-	-	-	-	-	-	-	-	-	-	-	-	-	-	-	-	-	-	-
ancestral343	0	-	-	-	-	-	-	-	-	-	-	-	-	-	-	-	-	-	-	-	-	-	-	-

Continued on next page

Table 3 – continued from previous page

A	B	C	D	E	F	G	H	I	J	K	L	M	N	O	P	Q	R	S	T	U	V	W	X	Y
ancestral344	0	-	-	-	-	-	-	-	-	-	-	-	-	-	-	-	-	-	-	-	-	-	-	-
ancestral345	0	-	-	-	-	-	-	-	-	-	-	-	-	-	-	-	-	-	-	-	-	-	-	-
ancestral346	0	-	-	-	-	-	-	-	-	-	-	-	-	-	-	-	-	-	-	-	-	-	-	-
ancestral347	0	-	-	-	-	-	-	-	-	-	-	-	-	-	-	-	-	-	-	-	-	-	-	-
ancestral348	0	-	-	-	-	-	-	-	-	-	-	-	-	-	-	-	-	-	-	-	-	-	-	-
ancestral349	0	-	-	-	-	-	-	-	-	-	-	-	-	-	-	-	-	-	-	-	-	-	-	-
ancestral350	0	-	-	-	-	-	-	-	-	-	-	-	-	-	-	-	-	-	-	-	-	-	-	-
ancestral351	0	-	-	-	-	-	-	-	-	-	-	-	-	-	-	-	-	-	-	-	-	-	-	-
ancestral352	1	-	-	-	-	-	-	-	-	-	-	-	-	-	-	-	1	0.86	132	151	33	1	0.589	0.346
ancestral353	3	86.34	0.18632	0.33011	-7.2	-1.84	-1.07	-0.77	1.4	0.34	0.26	-5.16	0	OTHER	0.639	OTHER	3	1.67	67	83	18	1	0.839	0.622
ancestral354	3	84.42	0.21686	0.26903	-9.57	-6.04	-5.27	-0.77	1.29	0.01	0.63	-2.73	0.000001	OTHER	0.778	OTHER	3	1.26	92	109	24	1	0.621	0.397
ancestral355	0	-	-	-	-	-	-	-	-	-	-	-	-	-	-	-	-	-	-	-	-	-	-	-
ancestral356	0	-	-	-	-	-	-	-	-	-	-	-	-	-	-	-	-	-	-	-	-	-	-	-
ancestral357	0	-	-	-	-	-	-	-	-	-	-	-	-	-	-	-	-	-	-	-	-	-	-	-
ancestral358	0	-	-	-	-	-	-	-	-	-	-	-	-	-	-	-	-	-	-	-	-	-	-	-
ancestral359	0	-	-	-	-	-	-	-	-	-	-	-	-	-	-	-	-	-	-	-	-	-	-	-
ancestral360	0	-	-	-	-	-	-	-	-	-	-	-	-	-	-	-	-	-	-	-	-	-	-	-
ancestral361	0	-	-	-	-	-	-	-	-	-	-	-	-	-	-	-	-	-	-	-	-	-	-	-
ancestral362	0	-	-	-	-	-	-	-	-	-	-	-	-	-	-	-	-	-	-	-	-	-	-	-
hs_cds363	1	-	-	-	-	-	-	-	-	-	-	-	-	-	-	-	1	0.6	2802	2820	916	54	0.591	0.275
hs_cds364	293	83.19	0.29658	0.52882	-170.7	-110.88	-111.09	0.21	1.29	-0.21	0.65	-1.96	0.005212	OTHER	3.286E-14	PROTEIN	293	3.11	488	732	90	8	0.59	0.474
hs_cds365	336	79.55	0.35368	0.40477	-108.24	-51.99	-50.84	-1.15	1.29	0.25	0.48	-3.23	0	OTHER	0	PROTEIN	336	2.22	425	447	94	9	0.59	0.431
hs_cds366	245	80.6	0.35557	0.46077	-39.77	-16.89	-16.87	-0.02	1.46	0.49	0.42	-3.84	0	OTHER	2.398E-14	PROTEIN	245	2.15	178	197	36	1	0.59	0.45
hs_cds367	2	98.15	0.01846	0.62158	-979.32	-964.1	-965.85	1.75	1.01	0.86	0.98	-2.82	0.000001	OTHER	-	-	2	0.55	2221	2246	756	37	0.589	0.258
hs_cds368	8	95.49	0.0871	0.55072	-454.53	-408.27	-411.37	3.1	1.09	-1.93	0.9	0.58	0.820264	RNA	3.331E-16	PROTEIN	8	0.66	1165	1194	376	19	0.59	0.282
hs_cds369	258	79.46	0.3491	0.53547	-156.74	-96.35	-98.45	2.1	1.27	-0.28	0.61	-1.84	0.007127	OTHER	4.564E-08	PROTEIN	258	2.37	437	815	82	7	0.59	0.455
hs_cds370	3	97.59	0.03324	0.71897	-516.07	-487.85	-489.53	1.69	1.03	0.59	0.95	-2.59	0.000003	OTHER	0.02	PROTEIN	3	0.52	967	992	331	18	0.589	0.258
hs_cds371	290	83.01	0.29677	0.48942	-117.74	-75.33	-74.12	-1.21	1.28	-0.4	0.64	-1.76	0.008723	OTHER	5.706E-10	PROTEIN	290	2.49	355	477	91	5	0.59	0.418
hs_cds372	1	-	-	-	-	-	-	-	-	-	-	-	-	-	-	-	1	0.6	2802	2820	916	54	0.591	0.275
hs_cds373	1	-	-	-	-	-	-	-	-	-	-	-	-	-	-	-	1	0.61	2283	2306	737	43	0.591	0.279
hs_cds374	1	-	-	-	-	-	-	-	-	-	-	-	-	-	-	-	1	0.57	2208	2231	724	33	0.591	0.273
hs_cds375	2	98.25	0.01748	0.44002	-315.8	-306.05	-311.05	5	1.01	0.26	0.97	-2.15	0.003076	OTHER	-	-	2	0.65	1030	1055	335	17	0.589	0.274
hs_cds376	11	90.74	0.17916	0.53033	-613.79	-502.23	-504.74	2.52	1.11	-0.69	0.82	-0.96	0.068659	OTHER	1.744E-12	PROTEIN	11	0.71	1619	1647	535	24	0.589	0.278
hs_cds377	1	-	-	-	-	-	-	-	-	-	-	-	-	-	-	-	-	-	-	-	-	-	-	-
hs_cds378	0	-	-	-	-	-	-	-	-	-	-	-	-	-	-	-	-	-	-	-	-	-	-	-
hs_cds379	1	-	-	-	-	-	-	-	-	-	-	-	-	-	-	-	1	0.59	2670	2689	876	49	0.59	0.272
hs_cds380	248	78.79	0.38016	0.50804	-138.16	-68.73	-67.19	-1.54	1.35	-0.64	0.5	-1.84	0.007077	OTHER	1.11E-16	PROTEIN	248	2.49	389	478	90	5	0.59	0.428
hs_cds381	337	84.03	0.28135	0.42118	-117.79	-76.14	-74.9	-1.23	1.28	0.35	0.65	-2.76	0.000001	OTHER	7.772E-16	PROTEIN	337	3.25	443	468	99	11	0.59	0.45
hs_cds382	250	86.15	0.25673	0.59612	-233.35	-148.81	-151.19	2.38	1.27	0.19	0.64	-2.72	0.000001	OTHER	0	PROTEIN	250	1.67	599	646	152	13	0.589	0.395
hs_cds383	304	81.74	0.3406	0.41272	-25.98	-10.99	-11.22	0.23	1.32	0.63	0.42	-4.08	0	OTHER	3.524E-11	PROTEIN	304	1.74	149	168	27	3	0.59	0.45
hs_cds384	66	82.07	0.34165	0.54476	-121.59	-68.96	-69.97	1.01	1.33	0.28	0.57	-2.83	0.000001	OTHER	6.505E-13	PROTEIN	66	1.16	343	365	105	6	0.59	0.334
hs_cds385	1	-	-	-	-	-	-	-	-	-	-	-	-	-	-	-	1	0.6	2631	2650	854	47	0.589	0.276
hs_cds386	323	80.65	0.33629	0.47296	-102.59	-65.05	-66.2	1.15	1.2	0.2	0.63	-2.41	0.000007	OTHER	2.897E-13	PROTEIN	323	1.84	327	421	87	6	0.59	0.395
hs_cds387	19	76.4	0.43514	0.50991	-450.99	-240.95	-236.93	-4.02	1.37	-0.66	0.53	-1.39	0.023333	OTHER	0	PROTEIN	19	1.04	1260	1320	392	26	0.591	0.32
hs_cds388	272	79.58	0.34846	0.51643	-111.64	-62.58	-64.67	2.09	1.15	0.43	0.56	-3.04	0	OTHER	3.14E-11	PROTEIN	272	1.68	332	558	96	5	0.591	0.361
hs_cds389	1	-	-	-	-	-	-	-	-	-	-	-	-	-	-	-	1	0.73	141	159	40	1	0.591	0.325
hs_cds390	224	73.86	0.44218	0.44178	-174.92	-67.47	-66.88	-0.59	1.37	0.48	0.39	-3.6	0	OTHER	0	PROTEIN	224	2.05	583	1332	112	10	0.59	0.451
hs_cds391	2	98.25	0.01748	0.44002	-315.8	-306.05	-311.05	5	1.01	0.17	0.97	-2.04	0.004121	OTHER	-	-	2	0.65	1030	1055	335	17	0.589	0.274
hs_cds392	1	-	-	-	-	-	-	-	-	-	-	-	-	-	-	-	-	-	-	-	-	-	-	-
hs_cds393	1	-	-	-	-	-	-	-	-	-	-	-	-	-	-	-	1	0.73	264	285	70	6	0.589	0.339
hs_cds394	1	-	-	-	-	-	-	-	-	-	-	-	-	-	-	-	-	-	-	-	-	-	-	-
hs_cds395	234	79.62	0.34338	0.4878	-57.85	-37.47	-37.62	0.14	1.22	-0.36	0.65	-1.53	0.015875	OTHER	0.0000209	PROTEIN	234	2.63	177	313	42	3	0.59	0.431
hs_cds396	294	80.5	0.32141	0.51559	-130.97	-85.78	-85.63	-0.15	1.27	-0.83	0.65	-1.02	0.059955	OTHER	2.257E-08	PROTEIN	294	2.73	376	578	84	5	0.59	0.444
hs_cds397	157	84.71	0.26692	0.62348	-174.7	-122.53	-125.62	3.09	1.12	0.88	0.7	-3.22	0	OTHER	0.00008376	PROTEIN	157	2.53	404	739	109	7	0.59	0.401
hs_cds398	1	-	-	-	-	-	-	-	-	-	-	-	-	-	-	-	-	-	-	-	-	-	-	-
hs_cds399	104	75.71	0.44618	0.48586	-165.49	-62.53	-61.09	-1.45	1.5	-0.28	0.38	-2.68	0.000002	OTHER	0	PROTEIN	104	1.82	540	563	90	8	0.59	0.464
hs_cds400	236	80.93	0.34395	0.4726	-41.37	-21.1	-20.47	-0.63	1.43	0.33	0.51	-3.21	0	OTHER	2.676E-14	PROTEIN	236	2.09	178	207	36	1	0.59	0.449
hs_cds401	318	78.1	0.35296	0.47069	-57.22	-34.33	-36	1.67	1.12	0.06	0.6	-2.32	0.000012	OTHER	6.916E-08	PROTEIN	318	2.03	202	223	41	4	0.59	0.441
hs_cds402	2	88	0.11997	0.53247	-484.27	-432.72	-432.97	0.25	1.06	-1.5	0.89	0.15	0.5922	RNA	-	-	2	0.68	1240	1265	416	21	0.59	0.27
hs_cds403	164	87.57	0.23668	0.45274	-89.55	-64.93	-64.61	-0.32	1.25	-0.21	0.73	-1.8	0.007883	OTHER	3.554E-07	PROTEIN	164	1.61	313	338	76	6	0.59	0.404
hs_cds404	147	81.74	0.32352	0.42757	-272.93	-161.45	-164.62	3.17	1.26	0.01	0.59	-2.45	0.000006	OTHER	0	PROTEIN	147	1.67	898	1345	196	17	0.59	0.422
hs_cds405	54	78.04	0.39949	0.52933	-177.77	-86.14	-88.13	1.99	1.37	-0.3	0.48	-2.31	0.000013	OTHER	1.029E-12	PROTEIN	54	2.14	518	605	132	8	0.59	0.403
hs_cds406	2	95.04	0.04958	0.53837	-1235.31	-1193.13	-1193.38	0.25	1	1.11	0.97	-3.03	0	OTHER	-	-	2	0.66	3561	3569	1037	66	0.588	0.307
hs_cds407	3	98.49	0.02081	0.54636	-268.67	-260.17	-260.4	0.23	1.01	-0.02	0.97	-1.8	0.007972	OTHER	0.02	PROTEIN	3	0.61	706	730	232	14	0.59	0.274
hs_cds408	3	98.98	0.01022	0.43462	-206.66	-200.66	-202.91	2.25	1.01	-0.45	0.97	-1.31	0.028235	OTHER	-	-	3	0.65	685	714	213	9	0.591	0.291
hs_cds409	277	79.4	0.38945	0.50329	-220.37	-121.21	-121.02	-0.2	1.38	1.29	0.55	-3.98	0	OTHER	0	PROTEIN	277	2.15	706	774	180	13	0.59	0.408
hs_cds410	1	-	-	-	-	-	-	-	-	-	-	-	-	-	-	-	1	0.58	354	376	117	4	0.589	0.276
hs_cds411	231	74.04	0.47736	0.4175	-36.86	-7.58	-7.5	-0.08	1.24	0.02	0.21	-3.82	0	OTHER	1.11E-16	PROTEIN	231	2.05	178	197	30	2	0.591	0.469
hs_cds412	270	88.42	0.21632	0.47512	-85.04	-61.81	-62.2	0.39	1.17	-1.76	0.73	0.14	0.580798	RNA	0.00007657	PROTEIN	270	2.96	250	273	39	3	0.59	0.491
ec_cds413	67	83.17	0.30114	0.51823	-17.25	-11.19	-11.38	0.19	1.08	-1.66	0.65	-0.02	0.479488	OTHER	0.045	PROTEIN	67	2.6	66	140	13	1	0.849	0.704
ec_cds414	0	-	-	-	-	-	-	-	-	-	-	-	-	-	-	-	-	-	-	-	-	-	-	-
ec_cds415	11	98.08	0.03611	0.56202	-405.27	-397.24	-397.5	0.26	1.05	-2.5	0.98	1.4	0.97548	RNA	0.000005102	PROTEIN	11	0.55	934	970	336	18	0.589	0.244
ec_cds416	11	98.2	0.03301	0.52918	-500.67	-479.58	-481.48	1.9	1.05	-1.57	0.96	0.15	0.588528	RNA	3.085E-09	PROTEIN	11	0.6	1288	1315	434	31	0.59	0.265
ec_cds417	0	-	-	-	-	-	-	-	-	-	-	-	-	-	-	-	-	-	-	-	-	-	-	-
ec_cds418	63	77.99	0.4146	0.55162	-578.07	-288.52	-288.61	0.09	1.39	-0.4	0.5	-2	0.004699	OTHER	0	PROTEIN	63	1.76	1432	1458	407	25	0.59	0.385
ec_cds419	134	82.69	0.32775	0.51896	-341.78	-192.73	-193.13	0.4	1.34	-1.18	0.56	-1.03	0.057644	OTHER	0	PROTEIN	134	3.63	955	1016	154	16	0.59	0.5
ec_cds420	214	80.84	0.36059	0.59438	-246.06	-147.7	-147.45	-0.25	1.34	-0.36	0.6	-1.73	0.009369	OTHER	0	PROTEIN	214	2.72	584	644	136	11	0.59	0.444
ec_cds421	277	86.94	0.24705	0.56601	-221.9	-164.23	-163.95	-0.28	1.25	-1.46	0.74	-0.05	0.458331	OTHER	1.11E-16	PROTEIN	277	4.36	563	633	111	11	0.59	0.489
ec_cds422	99	79.49	0.38368	0.51084	-252.63	-129.23	-127.4	-1.83	1.41	-0.39	0.51	-2.1	0.003601	OTHER	0	PROTEIN	99	1.42	715	744	191	17	0.589	0.379
ec_cds423	36	79.24	0.38678	0.48168	-150.86	-69.38	-64.38	-5	1.56	-0.27	0.46	-2.52	0.000004	OTHER	0	PROTEIN	36	1.35	487	510	147	8	0.589	0.345
ec_cds424	80	76.03	0.44058	0.49987	-117.81	-48.92	-51.5	2.57	1.4	-0.36	0.42	-2.38	0.000009	OTHER	0	PROTEIN	80	1.53	406	427	89	9	0.589	0.419
ec_cds425	0	-	-	-	-	-	-	-	-	-	-	-	-	-	-	-	-	-	-	-	-	-	-	-
ec_cds426	43	80.06	0.37283	0.51741	-38.83	-35.68	-35.93	0.25	1.14	-4.36	0.92	5	0.999998	RNA	0.134	OTHER	43	1.51	89	107	22	1	0.64	0.446
ec_cds427	0	-	-	-	-	-	-	-	-	-	-	-	-	-	-	-	-	-	-	-	-	-	-	-
ec_cds428	9	93.85	0.0993	0.50999	-412.74	-365.45	-365.19	-0.25	1.09	-1.95	0.89	0.62	0.834859	RNA	0.067	OTHER	9	0.64	1113	1138	369	19	0.589	0.273
ec_cds429	12	96.61	0.05496	0.5109	-83.33	-71.47	-72.47	1	1.05	0.59	0.86	-2.97	0	OTHER	0.468	OTHER	12	0.75	310	332	85	5	0.591	0.329
ec_cds430	42	80.11	0.37698	0.50509	-79.58	-64.14	-64.07	-0.07	1.32	-3.23	0.81	3.22	0.999808	RNA	0.00008636	PROTEIN	42	1.61	210	232	58	3	0.59	0.362
ec_cds431	22	82.6	0.32729	0.51621	-54.95	-48.29	-46.22	-2.08	1.3	-2.09	0.88	1.91	0.993685	RNA	0.002	PROTEIN	22	1.44	154	177	50	3	0.59	0.317

Continued on next page

Table 3 – continued from previous page

A	B	C	D	E	F	G	H	I	J	K	L	M	N	O	P	Q	R	S	T	U	V	W	X	Y
ec_cds432	33	79.27	0.37147	0.48	-22.07	-16.94	-15.78	-1.16	1.44	-4.2	0.77	4.12	0.999983	RNA	0.532	OTHER	33	3.15	55	72	17	0	1.011	0.837
ec_cds433	311	76.52	0.43771	0.5751	-513.26	-261.36	-258.82	-2.55	1.46	-1.5	0.51	-0.38	0.258292	OTHER	0	PROTEIN	311	4.21	1145	1211	279	22	0.59	0.466
ec_cds434	8	92.51	0.13426	0.47662	-303.66	-258.74	-260.34	1.6	1.16	-0.21	0.85	-1.64	0.011909	OTHER	0	PROTEIN	8	0.78	907	935	287	19	0.59	0.298
ec_cds435	70	88.91	0.19957	0.53764	-184.76	-130.97	-128.48	-2.49	1.21	0.28	0.71	-2.72	0.000001	OTHER	0.000000004	PROTEIN	70	1.11	498	522	161	7	0.59	0.315
ec_cds436	65	84.9	0.27786	0.5383	-253.32	-166.41	-169.41	2.99	1.2	0.77	0.66	-3.25	0	OTHER	6.22E-10	PROTEIN	65	0.99	698	734	223	13	0.589	0.306
ec_cds437	38	79.47	0.39331	0.51879	-83.83	-39	-41.54	2.54	1.35	0.02	0.47	-2.81	0.000001	OTHER	1.727E-08	PROTEIN	38	0.91	277	308	86	4	0.589	0.312
ec_cds438	330	85.51	0.26111	0.4896	-86.13	-55.24	-57.35	2.11	1.11	-2.13	0.64	0.35	0.70857	RNA	3.004E-07	PROTEIN	330	4.12	265	284	51	5	0.59	0.484
ec_cds439	94	79.27	0.38832	0.45795	-63.29	-29.49	-30.3	0.81	1.31	-0.1	0.47	-2.67	0.000002	OTHER	0.893	OTHER	94	2.07	219	240	51	3	0.59	0.421
ec_cds440	3	96.92	0.03075	0.53157	-378.08	-367.4	-366.65	-0.75	1.04	-1.64	0.97	0.27	0.662904	RNA	-	-	3	0.62	943	968	312	22	0.59	0.274
ec_cds441	0	-	-	-	-	-	-	-	-	-	-	-	-	-	-	-	-	-	-	-	-	-	-	-
ec_cds442	245	87.88	0.21969	0.56423	-199.94	-161.96	-163.07	1.1	1.24	-2.77	0.81	1.87	0.992958	RNA	2.665E-15	PROTEIN	245	3.07	468	489	146	9	0.59	0.398
ec_cds443	244	78.2	0.40657	0.57735	-192.55	-98.75	-98.5	-0.25	1.38	-2.58	0.51	0.88	0.910046	RNA	0	PROTEIN	244	3.73	445	470	121	7	0.59	0.435
ec_cds444	1140	-	-	-	-	-	-	-	-	-	-	-	-	-	-	-	1140	5.19	917	1018	169	17	0.59	0.506
ec_cds445	0	-	-	-	-	-	-	-	-	-	-	-	-	-	-	-	-	-	-	-	-	-	-	-
ec_cds446	381	80.52	0.36206	0.60362	-384.65	-231.35	-226.75	-4.6	1.38	-1.85	0.6	0.24	0.647011	RNA	0	PROTEIN	381	5.57	818	861	166	20	0.59	0.493
ec_cds447	157	83.13	0.31995	0.59817	-44.62	-27.91	-29.03	1.11	1.31	-1.23	0.63	-0.61	0.160158	OTHER	0.00001153	PROTEIN	157	2.82	113	130	32	2	0.59	0.414
ec_cds448	12	97.6	0.04509	0.53182	-426.64	-405.65	-405.41	-0.24	1.06	-1.71	0.95	0.34	0.702764	RNA	4.331E-11	PROTEIN	12	0.62	1150	1179	381	26	0.59	0.272
ec_cds449	0	-	-	-	-	-	-	-	-	-	-	-	-	-	-	-	-	-	-	-	-	-	-	-
ec_cds450	46	74.76	0.4839	0.46552	-124.55	-40.35	-40.44	0.09	1.39	-0.78	0.32	-2.21	0.00269	OTHER	0	PROTEIN	46	1.42	397	427	96	10	0.59	0.396
ec_cds451	110	69.48	0.57573	0.64549	-284.35	-90.4	-90.71	0.31	1.56	-0.4	0.32	-2.29	0.000014	OTHER	0	PROTEIN	110	4.22	589	615	159	14	0.59	0.444
ec_cds452	88	78.31	0.41696	0.57868	-337.77	-179.95	-183.52	3.57	1.4	-1.58	0.53	-0.26	0.327476	OTHER	0	PROTEIN	88	1.39	787	811	224	14	0.59	0.368
ec_cds453	11	98.22	0.03303	0.50917	-592.3	-567.07	-569.14	2.07	1.05	-3.71	0.96	2.71	0.999252	RNA	2.094E-07	PROTEIN	11	0.6	1555	1583	522	31	0.589	0.267
ec_cds454	0	-	-	-	-	-	-	-	-	-	-	-	-	-	-	-	-	-	-	-	-	-	-	-
ec_cds455	116	81.65	0.34362	0.55923	-469.3	-274.91	-266.84	-8.08	1.45	-0.7	0.59	-1.42	0.021229	OTHER	0	PROTEIN	116	1.92	1144	1170	363	23	0.59	0.36
ec_cds456	63	81.92	0.33901	0.54545	-588.42	-334.97	-335.9	0.93	1.35	-0.95	0.57	-1.23	0.035212	OTHER	0	PROTEIN	63	1.1	1516	1545	503	26	0.59	0.311
ec_cds457	63	85.11	0.27054	0.53513	-296.6	-185.23	-183.75	-1.48	1.27	-1.55	0.62	-0.48	0.211143	OTHER	0	PROTEIN	63	2.48	769	802	215	13	0.59	0.406
ec_cds458	2	97.03	0.02969	0.56393	-385.8	-368.02	-367.52	-0.5	1.03	-1.23	0.95	-0.34	0.278733	OTHER	-	-	2	0.6	943	973	311	16	0.591	0.275
ec_cds459	22	80.71	0.36172	0.57241	-556.92	-300.63	-296.53	-4.1	1.39	-1.19	0.54	-0.97	0.067014	OTHER	0	PROTEIN	22	1.44	1288	1325	404	16	0.59	0.349
ec_cds460	68	71.01	0.54065	0.53351	-102.12	-35.25	-33.48	-1.76	1.45	-0.68	0.35	-1.92	0.005782	OTHER	0	PROTEIN	68	3.1	285	311	61	5	0.59	0.464
ec_cds461	0	-	-	-	-	-	-	-	-	-	-	-	-	-	-	-	-	-	-	-	-	-	-	-
ec_cds462	0	-	-	-	-	-	-	-	-	-	-	-	-	-	-	-	-	-	-	-	-	-	-	-
ss_cds463	0	-	-	-	-	-	-	-	-	-	-	-	-	-	-	-	-	-	-	-	-	-	-	-
ss_cds464	0	-	-	-	-	-	-	-	-	-	-	-	-	-	-	-	-	-	-	-	-	-	-	-
ss_cds465	0	-	-	-	-	-	-	-	-	-	-	-	-	-	-	-	-	-	-	-	-	-	-	-
ss_cds466	0	-	-	-	-	-	-	-	-	-	-	-	-	-	-	-	-	-	-	-	-	-	-	-
ss_cds467	0	-	-	-	-	-	-	-	-	-	-	-	-	-	-	-	-	-	-	-	-	-	-	-
ss_cds468	0	-	-	-	-	-	-	-	-	-	-	-	-	-	-	-	-	-	-	-	-	-	-	-
ss_cds469	0	-	-	-	-	-	-	-	-	-	-	-	-	-	-	-	-	-	-	-	-	-	-	-
ss_cds470	0	-	-	-	-	-	-	-	-	-	-	-	-	-	-	-	-	-	-	-	-	-	-	-
ss_cds471	1	-	-	-	-	-	-	-	-	-	-	-	-	-	-	-	-	-	-	-	-	-	-	-
ss_cds472	3	86.43	0.1357	0.32659	-158.95	-130.35	-130.6	0.25	1.12	-2.36	0.82	0.97	0.928403	RNA	-	-	3	0.81	619	648	199	11	0.589	0.288
ss_cds473	0	-	-	-	-	-	-	-	-	-	-	-	-	-	-	-	-	-	-	-	-	-	-	-
ss_cds474	1	-	-	-	-	-	-	-	-	-	-	-	-	-	-	-	-	-	-	-	-	-	-	-
ss_cds475	4	89.61	0.1039	0.2835	-79.11	-63.4	-66.4	3	1.11	-0.09	0.8	-2.21	0.002656	OTHER	-	-	4	0.83	385	407	118	4	0.59	0.299
ss_cds476	32	76.07	0.45528	0.39904	-243.71	-117.16	-116.23	-0.92	1.45	0.28	0.48	-2.8	0.000001	OTHER	0	PROTEIN	32	2.92	843	922	160	12	0.59	0.48
ss_cds477	4	67.15	0.5502	0.40708	-384.65	-135.96	-146.84	10.87	1.47	0.61	0.35	-3.5	0	OTHER	0	PROTEIN	4	1.47	1398	1423	418	24	0.59	0.359
ss_cds478	3	87	0.13004	0.31238	-47.1	-34.1	-34.1	0	1.11	-1.02	0.72	-1.31	0.0282	OTHER	-	-	3	0.84	223	243	63	5	0.591	0.32
ss_cds479	1	-	-	-	-	-	-	-	-	-	-	-	-	-	-	-	-	-	-	-	-	-	-	-
ss_cds480	1	-	-	-	-	-	-	-	-	-	-	-	-	-	-	-	-	-	-	-	-	-	-	-
ss_cds481	12	71.39	0.53702	0.36338	-130.85	-37.37	-36.22	-1.15	1.53	-0.46	0.29	-2.55	0.000004	OTHER	0	PROTEIN	12	2.08	538	560	126	9	0.59	0.428
ss_cds482	3	81.63	0.18372	0.31709	-192	-146.5	-145.75	-0.75	1.17	-0.72	0.76	-1.23	0.035354	OTHER	-	-	3	0.86	811	835	245	16	0.589	0.309
ss_cds483	1	-	-	-	-	-	-	-	-	-	-	-	-	-	-	-	-	-	-	-	-	-	-	-
ss_cds484	3	83.26	0.16742	0.33069	-89.6	-71.1	-70.6	-0.5	1.14	1.09	0.79	-3.45	0	OTHER	-	-	3	0.86	442	465	132	8	0.59	0.309
ss_cds485	1	-	-	-	-	-	-	-	-	-	-	-	-	-	-	-	-	-	-	-	-	-	-	-
ss_cds486	8	67.5	0.57593	0.362	-168.26	-47.92	-44.64	-3.28	1.69	0.57	0.28	-3.7	0	OTHER	0	PROTEIN	8	1.68	648	672	192	6	0.589	0.363
ss_cds487	2	85.46	0.1454	0.33468	-142.76	-111.61	-114.61	3	1.12	0.27	0.78	-2.58	0.000003	OTHER	-	-	2	0.74	619	643	219	10	0.589	0.256
ss_cds488	0	-	-	-	-	-	-	-	-	-	-	-	-	-	-	-	-	-	-	-	-	-	-	-
ss_cds489	5	67.93	0.53054	0.3606	-265.66	-93.69	-104	10.31	1.45	0.8	0.35	-3.81	0	OTHER	0	PROTEIN	5	1.51	1057	1081	278	22	0.59	0.38
ss_cds490	1	-	-	-	-	-	-	-	-	-	-	-	-	-	-	-	-	-	-	-	-	-	-	-
ss_cds491	4	71.55	0.39641	0.34918	-167.34	-72.5	-70.59	-1.91	1.35	-0.14	0.43	-2.81	0.000001	OTHER	0	PROTEIN	4	1.15	718	743	197	11	0.591	0.353
ss_cds492	2	86.03	0.13965	0.30879	-178.73	-146.47	-144.22	-2.25	1.17	0.21	0.82	-2.31	0.000012	OTHER	-	-	2	0.79	802	826	271	11	0.589	0.272
ss_cds493	1	-	-	-	-	-	-	-	-	-	-	-	-	-	-	-	-	-	-	-	-	-	-	-
ss_cds494	6	68.6	0.56478	0.36569	-166.77	-40.2	-42.32	2.12	1.49	-0.78	0.24	-2.3	0.000013	OTHER	0	PROTEIN	6	1.62	662	685	174	8	0.59	0.388
ss_cds495	0	-	-	-	-	-	-	-	-	-	-	-	-	-	-	-	-	-	-	-	-	-	-	-
ss_cds496	0	-	-	-	-	-	-	-	-	-	-	-	-	-	-	-	-	-	-	-	-	-	-	-
ss_cds497	0	-	-	-	-	-	-	-	-	-	-	-	-	-	-	-	-	-	-	-	-	-	-	-
ss_cds498	2	84.15	0.15848	0.31284	-88.75	-63.78	-67.53	3.75	1.12	-0.57	0.72	-1.76	0.008701	OTHER	-	-	2	0.82	448	477	131	5	0.59	0.314
ss_cds499	5	67.65	0.5221	0.33685	-134.92	-49.24	-50.87	1.62	1.44	-0.34	0.36	-2.38	0.000009	OTHER	0	PROTEIN	5	1.48	574	603	141	11	0.591	0.389
ss_cds500	3	92.9	0.09782	0.43728	-116.3	-101.51	-100.53	-0.98	1.08	0.01	0.87	-1.99	0.00474	OTHER	0.0005335	PROTEIN	3	0.76	460	483	129	8	0.59	0.328
ss_cds501	1	-	-	-	-	-	-	-	-	-	-	-	-	-	-	-	1	0.66	594	618	182	12	0.589	0.298
ss_cds502	2	98.96	0.01045	0.58178	-270.91	-267.45	-267.45	0	1.01	1.75	0.99	-3.83	0	OTHER	-	-	2	0.57	670	694	233	14	0.591	0.253
ss_cds503	3	81.35	0.26274	0.50049	-101.81	-75.22	-76.47	1.24	1.13	0.14	0.74	-2.07	0.003806	OTHER	0.00001294	PROTEIN	3	0.89	313	334	96	4	0.59	0.315
ss_cds504	0	-	-	-	-	-	-	-	-	-	-	-	-	-	-	-	-	-	-	-	-	-	-	-
ss_cds505	4	81.04	0.18962	0.38226	-269.72	-202.07	-201.57	-0.5	1.14	1.37	0.75	-3.9	0	OTHER	-	-	4	0.84	1060	1085	341	16	0.589	0.289
ss_cds506	12	69.74	0.57394	0.36701	-42.36	-7.19	-6.22	-0.97	1.73	0.25	0.17	-3.89	0	OTHER	0	PROTEIN	12	2.15	193	212	45	4	0.59	0.432
ss_cds507	6	62.06	0.67429	0.32941	-166.69	-24.84	-25.04	0.2	1.56	-0.12	0.15	-3.14	0	OTHER	0	PROTEIN	6	2.01	741	823	174	12	0.59	0.419
ss_cds508	1	-	-	-	-	-	-	-	-	-	-	-	-	-	-	-	-	-	-	-	-	-	-	-
ss_cds509	12	77.6	0.41801	0.37772	-94.11	-39.89	-40.54	0.65	1.39	-0.11	0.42	-2.8	0.000001	OTHER	0	PROTEIN	12	1.47	388	413	89	8	0.59	0.411
ss_cds510	0	-	-	-	-	-	-	-	-	-	-	-	-	-	-	-	-	-	-	-	-	-	-	-
ss_cds511	1	-	-	-	-	-	-	-	-	-	-	-	-	-	-	-	-	-	-	-	-	-	-	-
ss_cds512	3	87.74	0.12263	0.32768	-251.81	-208.73	-209.98	1.25	1.13	-1.36	0.83	-0.32	0.290055	OTHER	-	-	3	0.81	1003	1028	313	17	0.589	0.293
hs_5putr_513	15	83.93	0.30314	0.47179	-16.35	-10.13	-9.63	-0.5	1.4	0.24	0.62	-2.68	0.000002	OTHER	0.445	OTHER	15	1.37	83	101	17	1	0.685	0.513
hs_5putr_514	20	86.33	0.24408	0.71992	-112.28	-93.58	-94.2	0.62	1.11	0.66	0.83	-2.34	0.000011	OTHER	0.387	OTHER	20	1	243	289	68	4	0.591	0.348
hs_5putr_515	14	81.29	0.3485	0.751	-49.07	-31.42	-30.9	-0.52	1.31	0.17	0.64	-2.26	0.000016	OTHER	0.653	OTHER	14	0.92	94	112	32	2	0.608	0.319
hs_5putr_516	1	-	-	-	-	-	-	-	-	-	-	-	-	-	-	-	1	1	73	90	22	1	0.702	0.433
hs_5putr_517	21	77.2	0.42204	0.70801	-87.88	-49.54	-52.73	3.19	1.23	0.83	0.56	-3.21	0	OTHER	0.267	OTHER	21	0.86	196	216	61	4	0.591	0.309
hs_5putr_518	31	75.02	0.46156	0.64282	-79.07	-37.3	-37	-0.3	1.45	0.16	0.47	-2.67	0.000002	OTHER	0.925	OTHER	31	1.06	194	227	61	5	0.59	0.325
hs_5putr_519	0	-	-	-	-	-	-	-	-	-	-	-	-	-	-	-	-	-	-	-	-	-	-	-

Continued on next page

Table 3 – continued from previous page

A	B	C	D	E	F	G	H	I	J	K	L	M	N	O	P	Q	R	S	T	U	V	W	X	Y
hs_5putr_520	1	-	-	-	-	-	-	-	-	-	-	-	-	-	-	-	-	-	-	-	-	-	-	-
hs_5putr_521	13	91.02	0.16051	0.44516	-106.82	-83.47	-82.22	-1.24	1.12	-0.13	0.78	-2	0.004676	OTHER	0.929	OTHER	13	0.84	385	407	113	5	0.589	0.315
hs_5putr_522	15	76.01	0.42775	0.47033	-16.11	-7.53	-7.62	0.09	1.44	0.32	0.47	-3.03	0	OTHER	0.974	OTHER	15	1.25	90	111	18	2	0.634	0.466
hs_5putr_523	1	-	-	-	-	-	-	-	-	-	-	-	-	-	-	-	-	-	-	-	-	-	-	-
hs_5putr_524	23	84.38	0.2728	0.70562	-75.93	-48.44	-47.3	-1.13	1.23	0.85	0.64	-3.48	0	OTHER	0.09	OTHER	23	0.82	163	183	52	2	0.591	0.304
hs_5putr_525	15	79.72	0.36867	0.82239	-38.47	-31.29	-31.58	0.29	1.09	0.81	0.81	-2.04	0.004157	OTHER	0.872	OTHER	15	0.98	81	112	29	1	0.7	0.396
hs_5putr_526	13	76.8	0.40568	0.82488	-74.23	-48.32	-49.27	0.95	1.14	0.16	0.65	-1.92	0.005743	OTHER	0.595	OTHER	13	0.77	113	208	37	1	0.589	0.299
hs_5putr_527	1	-	-	-	-	-	-	-	-	-	-	-	-	-	-	-	-	-	-	-	-	-	-	-
hs_5putr_528	11	84.3	0.28083	0.81588	-21.93	-18.55	-19.22	0.67	1.17	-0.04	0.85	-1.15	0.043045	OTHER	0.343	OTHER	11	2.47	52	67	18	0	1.066	0.859
hs_5putr_529	1	-	-	-	-	-	-	-	-	-	-	-	-	-	-	-	-	-	-	-	-	-	-	-
hs_5putr_530	21	85.6	0.26157	0.6696	-37.55	-25.22	-24.83	-0.39	1.21	0.46	0.67	-2.88	0.000001	OTHER	0.136	OTHER	21	0.85	95	112	31	2	0.602	0.314
hs_5putr_531	16	83.38	0.29886	0.74948	-48.88	-33.34	-34.17	0.84	1.21	0.13	0.68	-2.22	0.002575	OTHER	0.74	OTHER	16	0.83	97	118	31	1	0.59	0.307
hs_5putr_532	1	-	-	-	-	-	-	-	-	-	-	-	-	-	-	-	-	-	-	-	-	-	-	-
hs_5putr_533	6	85.55	0.25722	0.47941	-100.35	-68.03	-69.15	1.13	1.07	-0.09	0.68	-2.13	0.003247	OTHER	0.384	OTHER	6	0.78	341	363	106	7	0.59	0.299
hs_5putr_534	16	80.95	0.34034	0.71445	-27.13	-17.25	-18.28	1.03	1.22	0.58	0.64	-2.83	0.000001	OTHER	0.78	OTHER	16	1.85	66	81	21	0	0.851	0.624
hs_5putr_535	16	79.55	0.35714	0.45642	-11.35	-3.46	-3.47	0	1.4	-0.01	0.31	-3.78	0	OTHER	0.351	OTHER	16	1.68	71	90	17	1	0.794	0.61
hs_5putr_536	19	80.15	0.38442	0.48119	-49.67	-30.04	-32.77	2.72	1.09	-0.66	0.6	-1.23	0.035487	OTHER	0.353	OTHER	19	1.07	184	204	48	2	0.59	0.351
hs_5putr_537	35	77.21	0.42103	0.67244	-65.08	-33.68	-34.16	0.48	1.47	-0.66	0.52	-1.51	0.016917	OTHER	0.791	OTHER	35	0.96	142	162	49	2	0.59	0.295
hs_5putr_538	1	-	-	-	-	-	-	-	-	-	-	-	-	-	-	-	-	-	-	-	-	-	-	-
hs_5putr_539	10	87.46	0.21393	0.72664	-21.78	-18.81	-18.42	-0.39	1.21	-0.84	0.86	-0.38	0.258255	OTHER	0.628	OTHER	10	3.09	47	62	15	0	1.173	1.01
hs_5putr_540	0	-	-	-	-	-	-	-	-	-	-	-	-	-	-	-	-	-	-	-	-	-	-	-
hs_5putr_541	9	70.96	0.48104	0.51728	-116.28	-58.9	-61.32	2.42	1.13	0.33	0.51	-2.58	0.000003	OTHER	0.25	OTHER	9	1.3	392	488	78	6	0.59	0.416
hs_5putr_542	1	-	-	-	-	-	-	-	-	-	-	-	-	-	-	-	-	-	-	-	-	-	-	-
hs_5putr_543	33	77.22	0.41875	0.70111	-46.5	-22.95	-23.24	0.28	1.39	0.17	0.49	-2.77	0.000001	OTHER	0.667	OTHER	33	1.04	103	136	32	2	0.59	0.333
hs_5putr_544	1	-	-	-	-	-	-	-	-	-	-	-	-	-	-	-	-	-	-	-	-	-	-	-
hs_5putr_545	7	89.17	0.19252	0.73237	-14	-10.49	-10.27	-0.22	1.2	0.63	0.75	-2.98	0	OTHER	0.215	OTHER	7	4.35	40	54	12	1	1.367	1.229
hs_5putr_546	6	86.85	0.21652	0.74853	-27.96	-20.8	-21.64	0.84	1.06	0.39	0.74	-2.62	0.000002	OTHER	0.356	OTHER	6	1.77	65	81	20	1	0.863	0.647
hs_5putr_547	9	93.3	0.10644	0.77155	-206.78	-185.36	-185.55	0.19	1.09	0.06	0.9	-1.86	0.006811	OTHER	0.117	OTHER	9	0.52	342	373	122	7	0.591	0.246
hs_5putr_548	1	-	-	-	-	-	-	-	-	-	-	-	-	-	-	-	-	-	-	-	-	-	-	-
hs_5putr_549	1	-	-	-	-	-	-	-	-	-	-	-	-	-	-	-	1	0.64	356	386	103	7	0.59	0.314
hs_5putr_550	7	94.95	0.09209	0.48773	-63.33	-55.49	-58.1	2.61	1.02	-1.98	0.88	0.57	0.815994	RNA	0.164	OTHER	7	0.77	185	204	54	5	0.589	0.315
hs_5putr_551	15	80.13	0.34941	0.71912	-32.5	-23.22	-24	0.78	1.24	0.49	0.71	-2.28	0.000015	OTHER	0.898	OTHER	15	1.6	73	89	21	0	0.772	0.557
hs_5putr_552	10	94.34	0.09986	0.80338	-26.13	-26.03	-25.78	-0.25	1.15	1.04	1	-2.57	0.000003	OTHER	0.677	OTHER	10	1.59	59	75	19	1	0.946	0.714
hs_5putr_553	1	-	-	-	-	-	-	-	-	-	-	-	-	-	-	-	-	-	-	-	-	-	-	-
hs_5putr_554	10	93.55	0.1103	0.8257	-20.13	-17.05	-17.22	0.17	1	1.19	0.85	-3.5	0	OTHER	0.369	OTHER	10	2.29	49	64	15	1	1.127	0.937
hs_5putr_555	18	77.09	0.41037	0.4824	-16.04	-6.22	-5.25	-0.97	1.54	0.51	0.39	-3.78	0	OTHER	0.988	OTHER	18	1.38	86	104	14	1	0.662	0.531
hs_5putr_556	49	85.85	0.22758	0.46889	-202.35	-171.94	-173.75	1.81	1.04	0.15	0.85	-1.65	0.011565	OTHER	0.031	PROTEIN	49	2.28	617	1108	150	10	0.59	0.405
hs_5putr_557	1	-	-	-	-	-	-	-	-	-	-	-	-	-	-	-	-	-	-	-	-	-	-	-
hs_5putr_558	1	-	-	-	-	-	-	-	-	-	-	-	-	-	-	-	1	0.69	450	480	133	7	0.591	0.308
hs_5putr_559	1	-	-	-	-	-	-	-	-	-	-	-	-	-	-	-	-	-	-	-	-	-	-	-
hs_5putr_560	1	-	-	-	-	-	-	-	-	-	-	-	-	-	-	-	-	-	-	-	-	-	-	-
hs_5putr_561	7	94.4	0.10179	0.50263	-52.58	-45.21	-47.65	2.44	1.02	-1.45	0.86	-0.16	0.387846	OTHER	0.204	OTHER	7	0.78	161	180	46	4	0.59	0.325
hs_5putr_562	10	88.22	0.2011	0.72299	-23.17	-20.54	-19.9	-0.64	1.25	-0.81	0.89	-0.33	0.287113	OTHER	0.528	OTHER	10	2.65	50	65	16	0	1.106	0.926
hs_3putr_563	10	92.23	0.13973	0.41497	-60.24	-47.58	-49.45	1.86	1.06	0.43	0.79	-2.75	0.000001	OTHER	0.077	OTHER	10	0.92	259	281	66	4	0.588	0.358
hs_3putr_564	0	-	-	-	-	-	-	-	-	-	-	-	-	-	-	-	-	-	-	-	-	-	-	-
hs_3putr_565	36	84.45	0.28218	0.40395	-263.04	-179.95	-180.16	0.21	1.15	-1.11	0.68	-0.67	0.139103	OTHER	0.164	OTHER	36	1.29	954	1149	294	19	0.591	0.326
hs_3putr_566	103	78.74	0.38257	0.40738	-40.88	-22.77	-23.13	0.36	1.12	0.56	0.56	-3.05	0	OTHER	0.329	OTHER	103	2.57	194	303	33	2	0.59	0.474
hs_3putr_567	1	-	-	-	-	-	-	-	-	-	-	-	-	-	-	-	1	0.83	105	122	24	0	0.591	0.378
hs_3putr_568	28	81.04	0.30548	0.47	-120.99	-92.53	-93.32	0.79	1.18	-0.86	0.76	-0.44	0.228909	OTHER	0.352	OTHER	28	1.02	362	530	105	8	0.59	0.334
hs_3putr_569	26	86.03	0.25251	0.45694	-158.51	-91.91	-90.4	-1.51	1.22	0.09	0.58	-2.94	0	OTHER	0.433	OTHER	26	2.08	523	773	159	8	0.59	0.356
hs_3putr_570	0	-	-	-	-	-	-	-	-	-	-	-	-	-	-	-	-	-	-	-	-	-	-	-
hs_3putr_571	11	81.05	0.36083	0.48244	-547.63	-357.85	-362.48	4.63	1.22	-0.78	0.65	-0.89	0.081532	OTHER	0.888	OTHER	11	0.81	1602	1628	523	29	0.59	0.289
hs_3putr_572	12	91.04	0.15956	0.42004	-61.52	-43.24	-44.41	1.17	1.07	0.33	0.7	-3.02	0	OTHER	0.049	PROTEIN	12	0.87	258	280	72	3	0.59	0.333
hs_3putr_573	1	-	-	-	-	-	-	-	-	-	-	-	-	-	-	-	-	-	-	-	-	-	-	-
hs_3putr_574	14	81.86	0.32852	0.46058	-22.43	-8.34	-8.67	0.33	1.2	-0.25	0.37	-3.3	0	OTHER	0.686	OTHER	14	1.12	106	124	26	1	0.591	0.371
hs_3putr_575	76	76.73	0.39135	0.27577	-115.72	-42.92	-40.16	-2.76	1.32	0.72	0.37	-4.23	0	OTHER	0.074	OTHER	76	2.5	609	679	121	8	0.59	0.44
hs_3putr_576	86	85.62	0.2492	0.28484	-21.3	-13.73	-13.82	0.08	1.1	-0.48	0.64	-1.88	0.0063	OTHER	0.937	OTHER	86	2	125	151	32	1	0.59	0.392
hs_3putr_577	1	-	-	-	-	-	-	-	-	-	-	-	-	-	-	-	-	-	-	-	-	-	-	-
hs_3putr_578	1	-	-	-	-	-	-	-	-	-	-	-	-	-	-	-	1	0.65	2317	2339	754	41	0.591	0.276
hs_3putr_579	12	91.04	0.15956	0.42004	-61.52	-43.24	-44.41	1.17	1.07	0.33	0.7	-3.02	0	OTHER	0.09	OTHER	12	0.87	258	280	72	3	0.59	0.333
hs_3putr_580	0	-	-	-	-	-	-	-	-	-	-	-	-	-	-	-	-	-	-	-	-	-	-	-
hs_3putr_581	36	86.65	0.23873	0.39804	-267.77	-190.94	-190.9	-0.03	1.15	-1.38	0.71	-0.36	0.269478	OTHER	0.113	OTHER	36	1.26	978	1158	309	16	0.589	0.316
hs_3putr_582	69	81.92	0.33167	0.34621	-10.07	-2.25	-2.58	0.33	1	-0.51	0.22	-3.73	0	OTHER	0.428	OTHER	69	2.53	62	77	14	0	0.902	0.751
hs_3putr_583	1	-	-	-	-	-	-	-	-	-	-	-	-	-	-	-	-	-	-	-	-	-	-	-
hs_3putr_584	1	-	-	-	-	-	-	-	-	-	-	-	-	-	-	-	1	0.74	299	321	85	5	0.59	0.319
hs_3putr_585	10	92.23	0.13973	0.41497	-60.24	-47.58	-49.45	1.86	1.06	0.43	0.79	-2.75	0.000001	OTHER	0.069	OTHER	10	0.92	259	281	66	4	0.588	0.358
hs_3putr_586	1	-	-	-	-	-	-	-	-	-	-	-	-	-	-	-	1	0.77	2622	2641	757	46	0.589	0.314
hs_3putr_587	28	81.04	0.30548	0.47	-120.99	-92.53	-93.32	0.79	1.18	-0.84	0.76	-0.47	0.216698	OTHER	0.367	OTHER	28	1.02	362	530	105	8	0.59	0.334
hs_3putr_588	14	81.86	0.32852	0.46058	-22.43	-8.34	-8.67	0.33	1.2	-0.25	0.37	-3.3	0	OTHER	0.669	OTHER	14	1.12	106	124	26	1	0.591	0.371
hs_3putr_589	313	65.26	0.54511	0.45812	-42.12	-10.54	-10.02	-0.52	1.41	0.12	0.25	-3.45	0	OTHER	0.00005179	PROTEIN	313	3.47	162	310	23	2	0.59	0.506
hs_3putr_590	69	77.45	0.37054	0.26563	-111.33	-51.65	-48.62	-3.04	1.23	0.7	0.46	-3.82	0	OTHER	0.096	OTHER	69	2.6	583	929	137	11	0.59	0.415
hs_3putr_591	39	79.55	0.3708	0.27114	-125.55	-46.61	-47.68	1.07	1.3	0.91	0.37	-4.55	0	OTHER	0.155	OTHER	39	2.35	662	729	139	9	0.59	0.43
hs_3putr_592	1	-	-	-	-	-	-	-	-	-	-	-	-	-	-	-	-	-	-	-	-	-	-	-
hs_3putr_593	151	82.68	0.32615	0.56843	-141.15	-97.15	-101.3	4.15	1.25	-0.47	0.69	-1.25	0.033516	OTHER	3.331E-16	PROTEIN	151	1.54	371	393	102	8	0.591	0.377
hs_3putr_594	1	-	-	-	-	-	-	-	-	-	-	-	-	-	-	-	-	-	-	-	-	-	-	-
hs_3putr_595	1	-	-	-	-	-	-	-	-	-	-	-	-	-	-	-	1	0.71	897	927	272	11	0.589	0.297
hs_3putr_596	28	79.41	0.3599	0.56759	-53.27	-27.96	-27.3	-0.66	1.3	0.43	0.52	-3.21	0	OTHER	0.605	OTHER	28	1	157	176	48	3	0.59	0.322
hs_3putr_597	35	71.91	0.51098	0.35251	-138.56	-52.65	-54.82	2.17	1.3	-0.43	0.38	-2.2	0.00274	OTHER	0.849	OTHER	35	1.96	586	649	120	10	0.59	0.432
hs_3putr_598	1	-	-	-	-	-	-	-	-	-	-	-	-	-	-	-	-	-	-	-	-	-	-	-
hs_3putr_599	1	-	-	-	-	-	-	-	-	-	-	-	-	-	-	-	1	0.71	2731	2750	862	41	0.59	0.285
hs_3putr_600	76	87.07	0.2177	0.36086	-75.59	-63.24	-63.78	0.53	1.07	-0.01	0.84	-1.55	0.015217	OTHER	0.994	OTHER	76	2.14	345	473	75	8	0.59	0.429
hs_3putr_601	92	78.7	0.38776	0.4543	-104.04	-57.83	-58.51	0.68	1.29	-0.2	0.56	-2.04	0.004143	OTHER	0.0005126	PROTEIN	92	1.91	355	384	86	6	0.59	0.401
hs_3putr_602	86	85.62	0.2492	0.28484	-21.3	-13.73	-13.82	0.08	1.1	-0.48	0.64	-1.88	0.0063	OTHER	0.83	OTHER	86	2	125	151	32	1	0.59	0.392
hs_3putr_603	107	79.81	0.36456	0.41048	-48.09	-27.39	-27.15	-0.24	1.26	0.29	0.57	-2.73	0.000001	OTHER	0.47	OTHER	107	2.32	218	311	39	4	0.59	0.461
hs_3putr_604	70	78.08	0.36815	0.26265	-113.02	-50.43	-52.28	1.85	1.27	0.66	0.45	-3.84	0	OTHER	0.044	PROTEIN	70	2.53	613	679	113	8	0.59	0.452
hs_3putr_605	3	96.51	0.03486	0.47221	-162.96	-159.46	-159.46	0	1.01	-0.78	0.98	-0.73	0.120172	OTHER	-	-	3	0.72	544	567	158	6	0.589	0.31
hs_3putr_606	1	-	-	-	-	-	-	-	-	-	-	-	-	-	-	-	1	0.7	2754	2774	881	43	0.589	0.28
hs_3putr_607	14	80.17	0.35554	0.46435	-23.72	-8.34	-8.67	0.33	1.2	-0.29	0.35	-3.23	0	OTHER	0.67	OTHER	14	1.07	110	129	30	1	0.59	0.345

Continued on next page

Table 3 – continued from previous page

A	B	C	D	E	F	G	H	I	J	K	L	M	N	O	P	Q	R	S	T	U	V	W	X	Y
hs_3putr_608	77	82.44	0.32921	0.23088	-19.24	-8.18	-7.55	-0.63	1.38	0.06	0.43	-3.37	0	OTHER	0.478	OTHER	77	2.14	151	182	31	2	0.59	0.431
hs_3putr_609	1	-	-	-	-	-	-	-	-	-	-	-	-	-	-	-	1	0.59	171	191	55	1	0.589	0.277
hs_3putr_610	32	85.36	0.26017	0.43457	-82.7	-43.51	-43.27	-0.24	1.22	-0.55	0.53	-2.35	0.00001	OTHER	0.349	OTHER	32	2.19	310	353	78	5	0.59	0.401
hs_3putr_611	107	79.81	0.36456	0.41048	-48.09	-27.39	-27.15	-0.24	1.26	0.26	0.57	-2.69	0.000002	OTHER	0.413	OTHER	107	2.32	218	311	39	4	0.59	0.461
hs_3putr_612	1	-	-	-	-	-	-	-	-	-	-	-	-	-	-	-	1	0.75	298	319	85	3	0.59	0.322
ec_5putr_613	8	90.8	0.14638	0.42611	-4.9	-4.2	-4.83	0.63	1.11	-0.6	0.86	-1.03	0.05832	OTHER	0.342	OTHER	8	8	29	41	9	0	1.59	1.518
ec_5putr_614	17	83.66	0.28902	0.4513	-45.55	-37.42	-36.35	-1.07	1.31	-1.78	0.82	1.01	0.934612	RNA	0.31	OTHER	17	0.98	148	166	38	1	0.589	0.363
ec_5putr_615	26	79.11	0.40182	0.31789	-14.37	-7.29	-7.65	0.36	1.29	-0.58	0.51	-1.76	0.008719	OTHER	0.578	OTHER	26	1.77	84	100	20	0	0.676	0.483
ec_5putr_616	14	87.31	0.21928	0.29231	-16.16	-12.68	-12.23	-0.44	1.12	-1.12	0.78	-0.42	0.240778	OTHER	0.069	OTHER	14	0.82	108	142	29	1	0.591	0.335
ec_5putr_617	31	86.79	0.2297	0.35517	-4.47	-4.14	-3.78	-0.36	1.25	-0.78	0.93	-0.02	0.480396	OTHER	0.66	OTHER	31	11.87	34	47	9	0	1.595	1.542
ec_5putr_618	2	81.82	0.18182	0.4482	-2.9	-1.35	-1.35	0	1	0.92	0.47	-4.87	0	OTHER	-	-	2	2	44	60	7	1	0.929	0.811
ec_5putr_619	110	78.96	0.40428	0.35141	-25.15	-15.13	-16.05	0.92	1.28	-1.94	0.6	0.56	0.812661	RNA	0.998	OTHER	110	1.61	102	132	27	2	0.59	0.381
ec_5putr_620	47	81.24	0.36057	0.36245	-15.32	-9.68	-10.15	0.47	1.23	-0.32	0.63	-1.62	0.012752	OTHER	0.381	OTHER	47	2.52	83	99	15	0	0.684	0.562
ec_5putr_621	10	93.94	0.09834	0.32292	-0.79	0	0	0	0	0.78	0	-6.9	0	OTHER	0.337	OTHER	10	6.74	33	47	6	0	1.641	1.592
ec_5putr_622	57	80.45	0.37093	0.42294	-21.53	-16	-15.53	-0.47	1.53	-0.74	0.74	-0.39	0.253651	OTHER	0.308	OTHER	57	1.97	89	106	17	0	0.64	0.501
ec_5putr_623	72	77.68	0.42447	0.425	-25.42	-17.27	-17.47	0.2	1.32	-0.57	0.68	-0.7	0.130656	OTHER	0.723	OTHER	72	1.24	117	135	25	0	0.589	0.405
ec_5putr_624	25	86.9	0.23966	0.3902	-4.35	-3.57	-3.57	0	1	-0.26	0.82	-1.23	0.035291	OTHER	0.863	OTHER	25	4.23	42	56	4	0	1.305	1.266
ec_5putr_625	0	-	-	-	-	-	-	-	-	-	-	-	-	-	-	-	-	-	-	-	-	-	-	-
ec_5putr_626	24	85.21	0.26689	0.40124	-11.92	-6.57	-6.98	0.42	1.17	0.9	0.55	-4.06	0	OTHER	0.792	OTHER	24	1.41	80	95	26	0	0.708	0.434
ec_5putr_627	4	96.15	0.03846	0.25462	-2.1	-2.1	-2.1	0	1	-0.93	1	-0.43	0.235121	OTHER	-	-	4	4	26	40	3	0	1.426	1.385
ec_5putr_628	62	74.28	0.45444	0.49573	-88.68	-42.32	-40.92	-1.4	1.58	-1.22	0.48	-0.85	0.091017	OTHER	4.063E-14	PROTEIN	62	1.57	293	500	70	7	0.59	0.399
ec_5putr_629	32	76.98	0.4352	0.44981	-19.53	-11.74	-11.69	-0.05	1.35	-0.33	0.6	-1.42	0.021381	OTHER	0.15	OTHER	32	1.61	96	116	18	2	0.595	0.437
ec_5putr_630	60	79.96	0.37524	0.61403	-71.2	-36.84	-37.43	0.59	1.37	0.3	0.52	-2.97	0	OTHER	7.772E-16	PROTEIN	60	1.34	188	207	47	2	0.591	0.393
ec_5putr_631	60	80.07	0.37325	0.61078	-71.42	-36.84	-37.43	0.59	1.37	0.26	0.52	-2.93	0	OTHER	1.118E-13	PROTEIN	60	1.34	189	208	48	2	0.59	0.388
ec_5putr_632	22	92.52	0.14395	0.37402	-54.54	-45.84	-45.04	-0.8	1.2	-2.42	0.84	1.19	0.957459	RNA	0.837	OTHER	22	0.81	212	232	65	4	0.589	0.301
ec_5putr_633	24	92.22	0.15037	0.38806	-45.56	-40.1	-39.91	-0.19	1.18	-3.33	0.88	2.54	0.998812	RNA	0.997	OTHER	24	0.88	179	199	48	2	0.591	0.34
ec_5putr_634	6	89.06	0.20076	0.45302	-2.36	-2	-2	0	1	0.79	0.85	-2.6	0.000003	OTHER	0.656	OTHER	6	6	32	46	5	0	1.44	1.377
ec_5putr_635	19	92.54	0.14579	0.4364	-28.88	-25	-25.25	0.25	1.12	-2.57	0.87	1.54	0.983026	RNA	0.926	OTHER	19	0.78	92	110	30	1	0.621	0.317
ec_5putr_636	27	87.8	0.23299	0.3675	-73.86	-52.41	-53.59	1.18	1.24	-0.93	0.71	-0.98	0.06556	OTHER	0.316	OTHER	27	0.99	317	339	84	5	0.59	0.35
ec_5putr_637	10	92.41	0.13306	0.36798	-2.34	-1.62	-1.22	-0.4	1.25	0.18	0.69	-3.01	0	OTHER	0.486	OTHER	10	10	29	42	8	0	1.689	1.636
ec_5putr_638	36	74.72	0.47338	0.34504	-53	-17.33	-16.7	-0.63	1.49	-1.94	0.33	-0.7	0.128393	OTHER	0.873	OTHER	36	1.36	216	235	59	2	0.59	0.358
ec_5putr_639	16	86.65	0.25482	0.33821	-44.69	-28.44	-26.78	-1.66	1.33	-1.97	0.64	0.11	0.561664	RNA	0.104	OTHER	16	0.94	182	213	60	1	0.59	0.288
ec_5putr_640	24	84.03	0.30045	0.39891	-12.47	-6.79	-6.07	-0.72	1.31	-0.32	0.54	-2.41	0.000007	OTHER	0.855	OTHER	24	2.44	66	82	17	0	0.85	0.685
ec_5putr_641	49	80.42	0.31033	0.50952	-28.92	-15.54	-14.9	-0.64	1.17	-0.22	0.54	-2.49	0.000005	OTHER	0.647	OTHER	49	2.38	106	123	25	1	0.59	0.422
ec_5putr_642	297	75.77	0.39456	0.5435	-136.65	-79.97	-80.96	0.99	1.29	-1.06	0.59	-0.7	0.128695	OTHER	2.217E-10	PROTEIN	297	3.13	358	378	92	7	0.59	0.433
ec_5putr_643	50	83.39	0.29579	0.46154	-62.74	-34.45	-34.51	0.06	1.24	-1	0.55	-1.48	0.018273	OTHER	0.091	OTHER	50	1.62	220	267	48	2	0.59	0.419
ec_5putr_644	189	80.12	0.37893	0.53397	-56.38	-23.75	-25.95	2.2	1.23	-1.23	0.42	-1.53	0.016195	OTHER	1.008E-13	PROTEIN	189	2.43	159	177	42	3	0.59	0.424
ec_5putr_645	11	85.33	0.26568	0.44012	-4.53	-1.21	-0.68	-0.53	1.75	1.63	0.27	-6.27	0	OTHER	0.302	OTHER	11	3.09	49	63	11	0	1.128	1.005
ec_5putr_646	30	83.9	0.30365	0.49786	-1.84	0	0	0	0	0.75	0	-6.21	0	OTHER	0.745	OTHER	30	8.89	39	53	4	0	1.4	1.379
ec_5putr_647	183	85.71	0.26716	0.54021	-150.88	-106.17	-104.8	-1.37	1.33	-0.8	0.7	-1.04	0.056479	OTHER	5.945E-13	PROTEIN	183	2.76	415	490	108	6	0.59	0.426
ec_5putr_648	62	83.57	0.32608	0.50481	-24.35	-14.37	-14.77	0.39	1.33	-0.75	0.59	-1.44	0.020275	OTHER	0.065	OTHER	62	1.72	86	104	22	0	0.662	0.465
ec_5putr_649	200	81.1	0.36185	0.54199	-111.07	-61.9	-62.63	0.73	1.29	-0.77	0.56	-1.41	0.021718	OTHER	4.774E-15	PROTEIN	200	2.68	288	308	72	5	0.59	0.426
ec_5putr_650	4	93.94	0.08348	0.47691	-1.33	-0.9	-1.23	0.33	1	-0.17	0.67	-2.9	0.000001	OTHER	0.306	OTHER	4	4	22	36	5	0	1.461	1.361
ec_5putr_651	250	77.15	0.40556	0.51936	-139.82	-76.79	-77.12	0.33	1.35	-1.46	0.55	-0.35	0.273931	OTHER	0	PROTEIN	250	4.82	404	494	95	7	0.59	0.47
ec_5putr_652	250	79.81	0.38331	0.60376	-151.04	-93.74	-92.2	-1.54	1.39	-1.03	0.62	-0.63	0.154103	OTHER	0	PROTEIN	250	2.58	343	369	83	9	0.59	0.436
ec_5putr_653	254	77.57	0.41643	0.53313	-251.91	-143.74	-145.68	1.94	1.38	-1.36	0.57	-0.32	0.292691	OTHER	0	PROTEIN	254	2.11	648	782	179	14	0.59	0.397
ec_5putr_654	6	93.94	0.09834	0.30019	-2.08	0	0	0	0	-0.71	0	-5.3	0	OTHER	0.445	OTHER	6	6	33	47	6	0	1.612	1.542
ec_5putr_655	26	85.24	0.2895	0.38316	-34.47	-15.37	-16.41	1.03	1.27	-0.71	0.45	-2.45	0.000006	OTHER	0.993	OTHER	26	1.08	174	193	40	1	0.59	0.387
ec_5putr_656	2	96	0.04	0.3875	-0.65	-0.5	-0.5	0	1	0.57	0.77	-3.49	0	OTHER	-	-	2	2	25	40	4	0	0.977	0.864
ec_5putr_657	93	81.57	0.3507	0.57414	-78.98	-47.83	-47.08	-0.74	1.39	-1.6	0.61	-0.08	0.43613	OTHER	7.435E-09	PROTEIN	93	1.69	195	214	57	3	0.59	0.365
ec_5putr_658	2	80.77	0.19231	0.37308	-1.45	-0.95	-0.95	0	1	0.06	0.66	-2.75	0.000001	OTHER	-	-	2	2	26	41	4	0	0.857	0.744
ec_5putr_659	89	82.44	0.33156	0.51235	-125.93	-78.93	-78.05	-0.88	1.32	-0.98	0.63	-0.88	0.083724	OTHER	2.998E-15	PROTEIN	89	1.8	359	384	84	8	0.59	0.417
ec_5putr_660	254	83.1	0.32268	0.54018	-213.25	-139.42	-139.72	0.3	1.33	-0.78	0.65	-1.08	0.051542	OTHER	0	PROTEIN	254	3.32	548	633	139	11	0.59	0.439
ec_5putr_661	106	79.97	0.37735	0.48516	-48.67	-30.41	-30.38	-0.02	1.19	-1.42	0.62	-0.14	0.401024	OTHER	0.000009461	PROTEIN	106	1.87	165	187	29	4	0.59	0.456
ec_5putr_662	111	79.85	0.38579	0.45233	-36.07	-30.97	-29.75	-1.22	1.45	-0.96	0.86	0.63	0.837292	RNA	0.943	OTHER	111	1.17	134	163	33	1	0.591	0.377
ec_3putr_663	30	80.18	0.33733	0.51289	-20.85	-15.14	-14.53	-0.61	1.38	-1.45	0.73	0.33	0.699332	RNA	0.032	PROTEIN	30	1.75	66	83	18	3	0.85	0.635
ec_3putr_664	58	81.67	0.34781	0.47024	-13.45	-11.57	-11.43	-0.14	1.14	-2.33	0.86	2.22	0.997207	RNA	0.938	OTHER	58	3.92	56	71	10	1	0.994	0.914
ec_3putr_665	1	-	-	-	-	-	-	-	-	-	-	-	-	-	-	-	1	0.58	137	156	46	3	0.591	0.263
ec_3putr_666	10	93.48	0.11491	0.62262	-18.98	-17	-17.33	0.33	1	-4.59	0.9	3.85	0.999964	RNA	0.085	OTHER	10	4.84	36	50	10	0	1.511	1.393
ec_3putr_667	70	85.62	0.26619	0.45934	-24.93	-19.61	-20.08	0.47	1.15	-2.09	0.79	1.14	0.950854	RNA	0.757	OTHER	70	2.15	79	107	18	1	0.717	0.55
ec_3putr_668	50	80.91	0.35279	0.3618	-14.92	-14.2	-13.7	-0.5	1.2	-2.42	0.95	2.8	0.999401	RNA	0.674	OTHER	50	3.69	56	73	10	0	0.993	0.889
ec_3putr_669	3	84.71	0.20036	0.61825	-51.3	-43.87	-43.43	-0.44	1.06	-2.63	0.86	1.85	0.992531	RNA	0.673	OTHER	3	0.62	94	133	33	1	0.608	0.273
ec_3putr_670	47	70.4	0.56663	0.61376	-138.25	-61	-61.38	0.38	1.57	-0.75	0.44	-1.19	0.038462	OTHER	0	PROTEIN	47	2.09	321	350	100	4	0.59	0.37
ec_3putr_671	2	98.91	0.01087	0.54097	-32.05	-30.9	-30.9	0	1	-0.72	0.96	-1.02	0.059017	OTHER	-	-	2	0.7	92	110	30	1	0.62	0.308
ec_3putr_672	23	88.92	0.19679	0.25749	-13.38	-11.96	-10.8	-1.16	1.39	-1.06	0.89	-0.03	0.47461	OTHER	0.107	OTHER	23	1.73	79	96	17	1	0.716	0.535
ec_3putr_673	16	88.57	0.19233	0.54523	-13.41	-12.28	-12.39	0.11	1.11	-2.18	0.92	1.54	0.982947	RNA	0.52	OTHER	16	3.66	40	68	10	0	1.367	1.243
ec_3putr_674	25	88.24	0.21148	0.46242	-29.22	-25.17	-26.43	1.25	1.09	-3.28	0.86	2.7	0.999232	RNA	0.519	OTHER	25	1	84	102	24	1	0.675	0.416
ec_3putr_675	21	77	0.43171	0.49769	-28.57	-26.35	-24.8	-1.55	1.31	-3.65	0.92	4.5	0.999994	RNA	0.532	OTHER	21	2.88	71	87	18	0	0.794	0.617
ec_3putr_676	104	75.18	0.47548	0.63834	-94.82	-42.52	-45.09	2.56	1.38	-0.68	0.45	-1.64	0.012184	OTHER	9.567E-13	PROTEIN	104	2.54	218	237	54	4	0.59	0.431
ec_3putr_677	0	-	-	-	-	-	-	-	-	-	-	-	-	-	-	-	-	-	-	-	-	-	-	-
ec_3putr_678	133	83.9	0.2816	0.47086	-34.5	-28	-27.83	-0.16	1.19	-1.4	0.81	0.42	0.749167	RNA	0.000000053	PROTEIN	133	2.08	139	165	35	2	0.59	0.404
ec_3putr_679	290	76.86	0.42772	0.60735	-78.32	-29.49	-29.41	-0.08	1.36	-0.97	0.38	-1.87	0.00652	OTHER	2.665E-15	PROTEIN	290	4.05	212	267	35	2	0.59	0.505
ec_3putr_680	517	81.35	0.34765	0.62319	-313.85	-178.25	-178.75	0.5	1.31	-0.3	0.57	-2.04	0.004127	OTHER	0	PROTEIN	517	4.82	721	779	143	14	0.59	0.494
ec_3putr_681	17	83.33	0.29851	0.44986	-13.23	-12.32	-12.48	0.17	1	-4.11	0.93	4.42	0.999992	RNA	0.67	OTHER	17	6.1	37	50	9	0	1.472	1.4
ec_3putr_682	6	82.41	0.26729	0.46885	-5.8	-4.68	-4.43	-0.25	1.17	0.09	0.81	-1.6	0.013277	OTHER	0.45	OTHER	6	6	36	50	6	0	1.387	1.31
ec_3putr_683	9	96.34	0.06899	0.44229	-18.95	-17.42	-17.58	0.17	1	-4.92	0.92	4.03	0.999978	RNA	0.356	OTHER	9	2.04	51	66	18	1	1.085	0.823
ec_3putr_684	1	-	-	-	-	-	-	-	-	-	-	-	-	-	-	-	1	0.64	222	243	69	3	0.589	0.287
ec_3putr_685	39	77.29	0.44319	0.52114	-20.58	-21.11	-20.67	-0.44	1.27	-4.8	1.03	6.18	1	RNA	0.892	OTHER	39	15.42	41	55	11	0	1.335	1.272
ec_3putr_686	15	93.69	0.12597	0.52928	-21.43	-21.31	-21.17	-0.14	1.08	-5.48	0.99	5.13	0.999999	RNA	0.72	OTHER	15	4.87	37	51	13	0	1.472	1.341
ec_3putr_687	2	95.24	0.04762	0.63452	-6.3	-6.3	-6.3	0	1	-1.1	1	-0.17	0.377476	OTHER	-	-	2	2	21	35	5	0	1.077	0.901
ec_3putr_688	43	80.56	0.3698	0.53827	-21.72	-15.13	-15.38	0.25	1.08	-2.85	0.7	2.14	0.996528	RNA	0.199	OTHER	43	3.93	57	72	17	1	0.977	0.815
ec_3putr_689	18	78.79	0.40575	0.47818	-10.52	-10.49	-10.68	0.19	1.1	-1.71	1	2.4	0.998263	RNA	0.794	OTHER	18	11.13	39	53	10	0	1.4	1.353
ec_3putr_690	1	-	-	-	-	-	-	-	-	-	-	-	-	-	-	-	1	0.74	110	129	27	2	0.592	0.356
ec_3putr_691	42	81.51	0.33458	0.49639	-19.18	-18.5	-18.62	0.11	1.08	-4.3	0.96	4.92	0.999998	RNA	0.875	OTHER	42	4.86	50	65	12	0	1.106	1.002
ec_3putr_692	104	77.98	0.41359	0.55763	-60.58	-23.33	-23.17	-0.16	1.42	-0.27	0.39	-2.78	0.000001	OTHER	3.163E-12	PROTEIN	104	2.87	178	196	39	3	0.59	0.455
ec_3putr_693	108	83.01	0.32115	0.54646	-80.05	-40.1	-41.58	1.48	1.29	-2.38	0.5	0.17	0.600042	RNA	6.397E-11	PROTEIN	108	2.77	223	242	56	4	0.59	0.428
ec_3putr_694	7	92.94	0.12352	0.59916	-17.13	-17.13	-17.13	0	1	-4.22	1	3.92	0.99997	RNA	0.328	OTHER	7	5.5	35	49	8	0	1.551	1.474
ec_3putr_695	39	80.2	0.36957	0.50156	-27.48	-22.41	-23.18	0.78	1.12	-4.94	0.82	5.14	0.999999	RNA	0.853	OTHER	39	1.7	70	88	22	1	0.803	0.557

Continued on next page

Table 3 – continued from previous page

A	B	C	D	E	F	G	H	I	J	K	L	M	N	O	P	Q	R	S	T	U	V	W	X	Y
ec_3putr_696	5	91.77	0.13538	0.5147	-35.72	-35.7	-35.2	-0.5	1.16	-5.73	1	5.44	0.999999	RNA	0.992	OTHER	5	1.12	77	93	26	1	0.733	0.435
ec_3putr_697	70	83.08	0.33097	0.42827	-21.95	-18.78	-20.67	1.89	1.07	-5.52	0.86	5.69	1	RNA	0.083	OTHER	70	3.7	46	68	17	1	1.198	0.995
ec_3putr_698	209	86.65	0.22301	0.57768	-19.67	-16.6	-16.6	0	1	-4.67	0.84	4.24	0.999987	RNA	0.118	OTHER	209	209	28	85	11	0	1.53	1.225
ec_3putr_699	14	93.44	0.12758	0.45678	-25.38	-25.33	-25.22	-0.11	1.11	-4.97	1	4.7	0.999996	RNA	0.384	OTHER	14	1.7	60	76	19	1	0.931	0.678
ec_3putr_700	18	83.8	0.28603	0.4945	-27.3	-24.13	-25.43	1.31	1.1	-4.75	0.88	4.82	0.999997	RNA	0.831	OTHER	18	1.31	76	93	21	2	0.743	0.501
ec_3putr_701	13	91.58	0.15036	0.4783	-18.98	-19.12	-18.98	-0.14	1.15	-3.78	1.01	3.63	0.999935	RNA	0.311	OTHER	13	4.28	39	52	14	0	1.4	1.252
ec_3putr_702	8	97.22	0.04507	0.42794	-16.38	-16.75	-16.38	-0.38	1.14	-2.76	1.02	1.94	0.994105	RNA	0.375	OTHER	8	1.9	54	69	16	1	1.027	0.805
ec_3putr_703	24	79.63	0.3742	0.51288	-14.57	-10.68	-11.02	0.33	1	-2.6	0.73	2.01	0.995079	RNA	0.204	OTHER	24	4.72	46	77	11	1	1.197	1.09
ec_3putr_704	42	80.14	0.38538	0.56406	-23.02	-20.48	-20.37	-0.11	1.08	-4.79	0.89	5.37	0.999999	RNA	0.797	OTHER	42	8.04	47	62	12	0	1.173	1.062
ec_3putr_705	5	62.53	0.60602	0.54072	-111.92	-82.64	-81.45	-1.19	1.19	-6.08	0.74	6.75	1	RNA	0.268	OTHER	5	1.13	243	279	76	5	0.59	0.315
ec_3putr_706	21	89.47	0.18397	0.37186	-21.18	-20.71	-21.1	0.39	1.07	-7.12	0.98	6.66	1	RNA	0.858	OTHER	21	2.79	47	61	14	0	1.173	0.99
ec_3putr_707	10	92.97	0.1238	0.50556	-20.56	-20.14	-20.22	0.08	1.08	-6.22	0.98	5.71	1	RNA	0.76	OTHER	10	4.71	37	51	12	0	1.472	1.349
ec_3putr_708	24	83.68	0.3139	0.55579	-13.98	-13.98	-13.98	0	1	-2.67	1	3.14	0.999759	RNA	0.906	OTHER	24	24	32	44	7	0	1.617	1.591
ec_3putr_709	191	81.28	0.3428	0.49671	-59.19	-42.89	-42.95	0.06	1.16	-2.89	0.72	2.17	0.996782	RNA	9.228E-07	PROTEIN	191	2.06	165	193	45	2	0.59	0.392
ec_3putr_710	46	76.97	0.41504	0.45547	-22.67	-18.6	-18.55	-0.05	1.08	-4.94	0.82	5.35	0.999999	RNA	0.191	OTHER	46	2.78	57	75	16	0	0.977	0.796
ec_3putr_711	5	87.62	0.17647	0.4123	-17.63	-18.32	-18.1	-0.22	1.09	-4.58	1.04	4.72	0.999996	RNA	0.525	OTHER	5	5	35	48	11	0	1.456	1.344
ec_3putr_712	15	84.81	0.26892	0.70876	-20.07	-18.3	-18.3	0	1	-3.61	0.91	3.62	0.999933	RNA	0.809	OTHER	15	9.39	34	47	12	0	1.595	1.483

References

1. Statistics of of sequence similarity scores. `http://www.ncbi.nlm.nih.gov/BLAST/tutorial/`. Accessed: 2016-03-19.
2. David L Wheeler, Tanya Barrett, Dennis A Benson, Stephen H Bryant, Kathi Canese, Vyacheslav Chetvernin, Deanna M Church, Michael DiCuccio, Ron Edgar, Scott Federhen, et al. Database resources of the national center for biotechnology information. *Nucleic acids research*, 35(suppl 1):D5–D12, 2007.
3. Travis J Wheeler, Jody Clements, Sean R Eddy, Robert Hubley, Thomas A Jones, Jerzy Jurka, Arian FA Smit, and Robert D Finn. Dfam: a database of repetitive dna based on profile hidden markov models. *Nucleic acids research*, 41(D1):D70–D82, 2013.
4. Heladia Salgado, Martin Peralta-Gil, Socorro Gama-Castro, Alberto Santos-Zavaleta, Luis Muñiz-Rascado, Jair S García-Sotelo, Verena Weiss, Hilda Solano-Lira, Irma Martínez-Flores, Alejandra Medina-Rivera, et al. Regulondb v8. 0: omics data sets, evolutionary conservation, regulatory phrases, cross-validated gold standards and more. *Nucleic acids research*, 41(D1):D203–D213, 2013.
5. Dennis A Benson, Mark Cavanaugh, Karen Clark, Ilene Karsch-Mizrachi, David J Lipman, James Ostell, and Eric W Sayers. Genbank. *Nucleic acids research*, page gks1195, 2012.
6. Andrew Yates, Wasiu Akanni, M Ridwan Amode, Daniel Barrell, Konstantinos Billis, Denise Carvalho-Silva, Carla Cummins, Peter Clapham, Stephen Fitzgerald, Laurent Gil, et al. Ensembl 2016. *Nucleic acids research*, 44(D1):D710–D716, 2016.
